# A Membrane‐Targeting Aggregation‐Induced Emission Probe for Monitoring Lipid Droplet Dynamics in Ischemia/Reperfusion‐Induced Cardiomyocyte Ferroptosis

**DOI:** 10.1002/advs.202309907

**Published:** 2024-05-02

**Authors:** Yihui Wang, Yuan Song, Lingling Xu, Wuqi Zhou, Wenyuan Wang, Qiaofeng Jin, Yuji Xie, Junmin Zhang, Jing Liu, Wenqian Wu, He Li, Le Liang, Jing Wang, Yali Yang, Xiongwen Chen, Shuping Ge, Tang Gao, Li Zhang, Mingxing Xie

**Affiliations:** ^1^ Department of Ultrasound Medicine Union Hospital Tongji Medical College Huazhong University of Science and Technology Wuhan 430022 China; ^2^ Clinical Research Center for Medical Imaging No. 1277 Jiefang Avenue Wuhan Hubei 430022 China; ^3^ Hubei Province Key Laboratory of Molecular Imaging Wuhan 430022 China; ^4^ The Institute of Advanced Studies Wuhan University Wuhan 430072 China; ^5^ Department of Biopharmaceuticals & Tianjin Key Laboratory on Technologies Enabling Development of Clinical Therapeutics and Diagnostics School of Pharmacy Tianjin Medical University Tianjin 300052 China; ^6^ Department of Cardiology Tianjin Medical University General Hospital Tianjin 300052 China; ^7^ Drexel University College of Medicine and St. Christopher's Hospital for Children 3601 A Street Philadelphia PA 19134 USA; ^8^ Shenzhen Huazhong University of Science and Technology Research Institute Shenzhen 518029 China

**Keywords:** aggregation‐induced emissions, ferroptosis, lipid droplet imaging, lipophagy, myocardial ischemia/reperfusion injury

## Abstract

Myocardial ischemia/reperfusion injury (MIRI) is the leading cause of irreversible myocardial damage. A pivotal pathogenic factor is ischemia/reperfusion (I/R)‐induced cardiomyocyte ferroptosis, marked by iron overload and lipid peroxidation. However, the impact of lipid droplet (LD) changes on I/R‐induced cardiomyocyte ferroptosis is unclear. In this study, an aggregation‐induced emission probe, **TPABTBP** is developed that is used for imaging dynamic changes in LD during myocardial I/R‐induced ferroptosis. **TPABTBP** exhibits excellent LD‐specificity, superior capability for monitoring lipophagy, and remarkable photostability. Molecular dynamics (MD) simulation and super‐resolution fluorescence imaging demonstrate that the **TPABTBP** is specifically localized to the phospholipid monolayer membrane of LDs. Imaging LDs in cardiomyocytes and myocardial tissue in model mice with MIRI reveals that the LD accumulation level increase in the early reperfusion stage (0–9 h) but decrease in the late reperfusion stage (>24 h) via lipophagy. The inhibition of LD breakdown significantly reduces the lipid peroxidation level in cardiomyocytes. Furthermore, it is demonstrated that chloroquine (CQ), an FDA‐approved autophagy modulator, can inhibit ferroptosis, thereby attenuating MIRI in mice. This study describes the dynamic changes in LD during myocardial ischemia injury and suggests a potential therapeutic target for early MIRI intervention.

## Graphical abstract

A a membrane‐targeting aggregation‐induced emission probe is devised to monitor lipid droplet (LD) dynamics during ischemia/reperfusion‐induced cardiomyocyte ferroptosis. These results reveals an increase in LDs during the early reperfusion stage (0–9 h), followed by degradation through lipophagy during the late reperfusion stage (>24 h). Furthermore, inhibiting LD degradation markedly mitigates myocardial ischemia/reperfusion injury in vivo by suppressing ferroptosis.

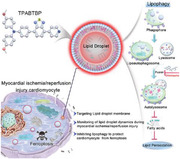

## 1 Introduction

Acute myocardial infarction remains a predominant cause of global mortality and morbidity.^[^ ^1^ ^]^ The principal approach to limit infarct size and subsequent ventricular remodeling involves reperfusion therapy, typically achieved through primary percutaneous coronary intervention (PCI) or thrombolysis.^[^ ^2^ ^]^ However, reperfusion can provoke irreversible damage to cardiomyocytes, a phenomenon recognized as myocardial ischemia/reperfusion injury (MIRI).^[^ ^3^ ^]^ Despite advancements in PCI techniques and the availability of antiplatelet and antithrombotic medications, there are currently no established treatments that conclusively mitigate MIRI. There is an urgent need for the development of novel cardioprotective strategies aimed at mitigating MIRI and improving clinical outcomes in AMI patients.

Ferroptosis, characterized by iron‐dependent lipid peroxidation, has emerged as a novel form of regulated cell death.^[^ ^4^ ^]^ Accumulating evidence indicates that ferroptosis is the primary modality of cardiomyocyte death in MIRI.^[^ ^5^ ^]^ Fang et al. demonstrated for the first time that during the ischemia/reperfusion (I/R) period, the degradation of ferritin releases iron, triggering the iron‐mediated Fenton reaction in mitochondria, which leads to lipid peroxidation and subsequent cardiac injury.^[^ ^6^ ^]^ Subsequent studies by Hiroyuki Tsutsui et al. reveal that hypoxia/reoxygenation augments Heme oxygenase 1 level, inducing iron overload in the endoplasmic reticulum, thereby fostering excessive lipid peroxide generation and subsequent ferroptosis in cardiomyocytes.^[^ ^7^ ^]^ The inhibition of lipid peroxidation represents a promising strategy for mitigating ferroptosis and enhancing cardiac function in MIRI.^[^ ^8^ ^]^ Lipid droplets (LDs) act as modulators of lipid peroxidation, and their degradation increases free fatty acid (FFA) production and promotes ferroptosis.^[^ ^9^ ^]^ Nevertheless, the precise alterations in LD metabolism associated with ferroptosis following MIRI remain incompletely understood. Hence, visualizing LD dynamics assumes critical importance not only in elucidating the interplay between LDs and ferroptosis progression but also in fostering the development of novel ferroptosis inhibitors for MIRI treatment.

Fluorescence imaging (FI) using small‐molecule probes has emerged as a valuable technique for visualizing biomolecules at subcellular levels, particularly in vivo, owing to its non‐invasiveness, real‐time, excellent spatial resolution, and high signal‐to‐noise ratio (SNR). Although commercial probes like Nile Red and BODIPY493/503 are widely utilized for LD staining, they often exhibit non‐specific staining of other hydrophobic cellular structures, thereby compromising the signal‐to‐noise ratio. Recently, numerous fluorescent probes tailored specifically for LD imaging have been developed (Table S1, Supporting Information). However, these probes have certain limitations in their photophysical properties, which impede their potential applications as sensitive and specific fluorescent probes. Most of these fluorescent probes consist of highly hydrophobic conjugated aromatic groups, which tend to aggregate in an intracellular milieu, leading to an aggregation‐induced quenching (ACQ) effect on the fluorescence signal.^[^ ^10^ ^]^ Consequently, they are typically used at low concentrations due to the ACQ effect, resulting in low photo‐bleaching resistance.^[^ ^11^ ^]^ Furthermore, the majority of LD‐specific probes exhibit a small Stokes shift (<100 nm), which may compromise image quality in confocal microscopy measurements.^[^ ^12^ ^]^ Given their remarkable brightness and high photostability, fluorescent probes featuring aggregation‐induced emission (AIE) characteristics have attracted considerable attention for bioimaging applications.^[^ ^13^ ^]^ Recently, several AIE‐based fluorescent probes have been developed for LD imaging at both cellular and tissue levels. These probes not only enable LD imaging but also facilitate the differentiation of cancer cells and the visualization of LDs in tumor tissues, liver tissues, or atherosclerotic plaque.^[^ ^14^ ^]^ However, there remains a significant gap in understanding the in vivo dynamics and accumulation of LDs during disease progression and treatment. Specifically, there is a lack of in vivo investigation into dynamic LD changes during MIRI. Therefore, it is imperative to employ AIE probes for specific LD recognition and integrate this approach with MIRI models to elucidate the correlation between ischemia/reperfusion‐induced cardiomyocyte ferroptosis and LD abnormalities.

**Scheme 1:**
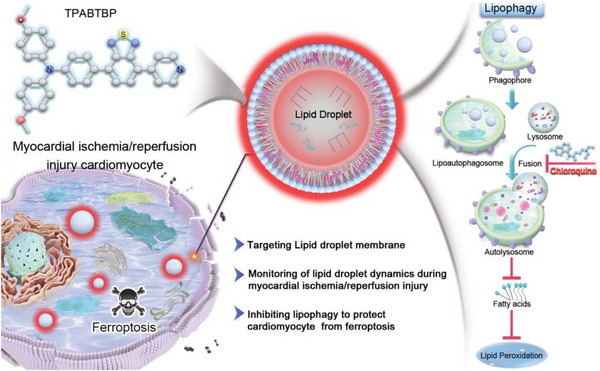
Schematic diagram of membrane‐targeting aggregation‐induced emission probe for monitoring lipid droplet (LD) dynamics in ischemia/reperfusion‐induced cardiomyocyte ferroptosis. The LD‐targeting molecular probe **TPABTBP** was trapped in the phospholipid monolayer of LD. **TPABTBP** was employed to monitor the dynamics of LD during MIRI, indicating the involvement of LD degradation through lipophagy. Meanwhile, inhibiting lipophagy has been shown to protect cardiomyocytes from ferroptosis.

Herein, we have developed LD‐targeting molecular probes (**TPABTBP**, **PMBTDP**, and **BTDPP**) by combining intramolecular charge transfer (ICT) with AIE. Among these probes, **TPABTBP** showed polarity‐sensitive fluorescence emission and excellent performance for specifically imaging LDs in live cells. We reveal for the first time that the probe **TPABTBP** is trapped in the phospholipid LD monolayer. Facilitated by **TPABTBP**, we revealed that the LD accumulation level increased in the early reperfusion stage but decreased in the late reperfusion stage via lipophagy. The degradation of LDs promoted lipid peroxidation, exacerbating the myocardial injury. Importantly, we demonstrate that chloroquine (CQ) inhibits LD breakdown and ameliorates MIRI in murine models (**Scheme** **1**).

## 2 Results and Discussion

### 2.1 Design and Synthesis of AIE‐based Probes for Imaging LDs

**Figure 1:**
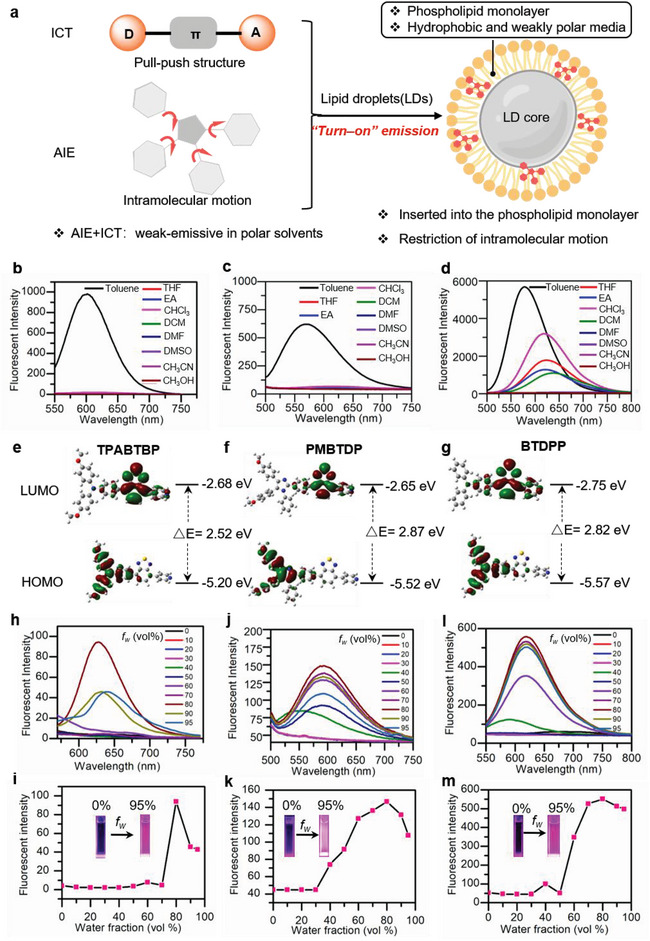
The design strategy of the proposed LD probes and their photophysical properties. a) Polarity responsiveness and intramolecular motion restriction were leveraged to design probes with aggregation‐induced emission for use in LD imaging. The fluorescence emission spectra of b) **TPABTBP**, c) **PMBTDP**, and d) **BTDPP** in different solvents. Amplitude plots of highest occupied molecular orbital (HOMO) and lowest occupied molecular orbital (LUMO) energy levels of e) **TPABTBP**, f) **PMBTDP**, and g) **BTDPP** as calculated by using the PBE0/Def2‐svp basis set. Photoluminescence (PL) spectra of h) **TPABTBP**, j) **PMBTDP**, and l) **BTDPP** in DMSO/water mixtures with different water fractions (*f*_w_). Plots showing maximum PL intensity of i) **TPABTBP**, k) **PMBTDP**, and m) **BTDPP** at various DMSO/water mixtures. Inset: photographs of **TPABTBP**, **PMBTDP**, or **BTDPP** fluorescence in DMSO solution (left) and DMSO/water mixtures (right) with a 95% water content. A hand‐held UV lamp was used to obtain these photographs. Concentration: 10 µM; *λ*_ex_ = 480 nm.

LDs consist of a neutral lipid core containing triacylglycerols (TAGs) and steryl esters, encased within a phospholipid monolayer adorned with specific proteins on its surface. This inherent high hydrophobicity makes LDs an ideal target for imaging.^[^ ^15^ ^]^ Traditionally, fluorescent probes designed for LD imaging utilize highly lipophilic molecular scaffolds with intramolecular charge transfer (ICT) characteristics. However, such probes often exhibit fluorescence in polar media, resulting in significant background emission. To address this challenge, we developed LD‐targeted probes with both ICT and AIE properties based on the following considerations. ICT‐based probes demonstrate a blue shift in emission wavelength and increased fluorescence intensity in nonpolar environments, making them ideal for LD imaging with minimal background interference. Additionally, ICT‐based probes offer substantial Stokes shifts, contrasting with the limited Stokes shifts observed in commercial LD probes like Nile Red and BODIPY 493/503, which enhances LD image quality by reducing incident light interference. Conventional dyes are susceptible to aggregation‐caused quenching (ACQ), leading to poor resistance to photo‐bleaching. In contrast, AIEgens aggregate within cellular LDs, allowing them to emit fluorescence and exhibit excellent photostability (**Figure** **1**a). The molecular structure of the fluorescent probe, illustrated in Figure S1 (Supporting Information), comprises tetra‐aryl imidazolyl or triphenylamine as the electron donor (D) and benzothiadiazole and pyridine as the acceptor (A), forming a typical D–A molecular structure. The rotation of the electron donor (D) tetra‐aryl imidazolyl and acceptor (A) triphenylamine imparts the molecule with both AIE and ICT properties.

**TPABTBP**, **PMBTDP**, and **BTDPP** were synthesized via Suzuki coupling of (4‐(4,5‐bis(4‐methoxyphenyl)−1‐phenyl‐1H‐imidazol‐2‐yl) phenyl) boronic acid, (4‐(diphenylamino)phenyl)boronic acid and (4‐(bis(4‐methoxyphenyl)amino)phenyl)boronic acid with 4‐bromo‐7‐(pyridin‐4‐yl)benzo[c][1,2,5]thiadiazole, respectively (Schemes S1–S3, Supporting Information). Detailed experimental protocols can be found in the Supporting Information. The structures of **TPABTBP**, **PMBTDP**, and **BTDPP** were thoroughly characterized and confirmed through NMR and high‐resolution mass spectrometry (Figures S2–S11, Supporting Information).

### 2.2 Characteristics of the Photophysical Properties

Molecules featuring a donor (D)–π–acceptor (A) configuration exhibit a significant solvatochromic effect, as evidenced by changes in their photophysical properties in response to variations in solvent polarity. Therefore, we analyzed the photoluminescence (PL) spectra of **TPABTBP**, **PMBTDP**, and **BTDPP** in solvents with different polarities. These compounds exhibited high‐intensity fluorescence in a weak polar solvent, toluene, with emission wavelengths of 600, 575, and 580 nm, respectively (Figure 1b–d). The fluorescence quantum yields of **TPABTBP**, **PMBTDP**, and **BTDPP** in toluene were 50.9%, 99.97%, and 98.95%, respectively (Table S2, Supporting Information).

In toluene, **TPABTBP** showed a red shift of emissions from 600 to 645 nm in methanol, accompanied by a sharp decrease in fluorescence intensity. With increasing solvent polarity, the fluorescence intensity of **PMBTDP** and **BTDPP** decreased markedly, accompanied by a redshift in the emission wavelength. These solvent polarity‐dependent fluorescence emission properties were attributed to the ICT effect contributed by the donation of electrons (methoxy‐substituted triphenylamine, tetra‐aryl imidazole, and triphenylamine) to an electron‐withdrawing group in the benzothiadiazole core. ICT molecules display distinct fluorescence responses in polar and nonpolar solvents. In polar solvents, they experience a more significant relaxation effect, causing a bathochromic‐shifted fluorescence with reduced quantum yield. In contrast, nonpolar solvents induce less ICT effect, leading to emissions at shorter wavelengths with increased fluorescence intensity.^[^ ^16^ ^]^ Furthermore, the ICT effect of **TPABTBP**, **PMBTDP**, and **BTDPP** was confirmed by density functional theory calculations (Figure 1e–g). The lowest unoccupied molecular orbital (LUMO) of **TPABTBP**, **PMBTDP**, and **BTDPP** were predominately occupied on the strong electron‐accepting group of the benzothiadiazole core, while the highest occupied molecular orbital (HOMO) was mainly concentrated in electron‐donating group (methoxy‐substituted triphenylamine, tetra‐aryl imidazole, and triphenylamine). The pull−push structure of **TPABTBP**, **PMBTDP**, and **BTDPP**, along with the separation between LUMO and HOMO, imparts the probes with the ICT character, thus displaying the large Stokes shift and solvatochromism.^[^ ^14^, ^17^ ^]^ The calculated band gaps (△*E*) of **PMBTDP**, **BTDPP**, and **TPABTBP** were 2.87, 2.82, and 2.52 eV, respectively, suggesting a gradual enhancement of the ICT effect. LD polarity has been shown to be close to that of toluene.^[^ ^18^ ^]^ The fluorescence responses of the compounds **TPABTBP** and **PMBTDP** in toluene solution indicated that they were suitable for LD imaging.

In addition to solvatochromism, **TPABTBP**, **PMBTDP**, and **BTDPP** showed AIE. In DMSO, they also showed weak fluorescence emission in DMSO solution. **TPABTBP** and **PMBTDP** showed fluorescence quantum yields below 0.1% in DMSO, while **BTDPP** demonstrated a fluorescence quantum yield of 2.08% in DMSO (Table S2, Supporting Information). When the water content was increased to 80%, the fluorescence emission intensity of **TPABTBP**, **PMBTDP**, and **BTDPP**, reached the maximum level (Figure 1h–m). In aqueous solutions, **TPABTBP**, **PMBTDP**, and **BTDPP** tended to aggregate due to their poor solubility. This aggregation limited their intramolecular motion and triggered AIE. Unexpectedly, we observed a reduction in emission intensity and a slight redshift in the emission spectra of **TPABTBP**, **PMBTDP**, and **BTDPP** when the water content was increased from 80% to 95%. The fluorescence quantum yields of **TPABTBP**, **PMBTDP**, and **BTDPP** in H_2_O (containing 5% DMSO) were 6.53%, 12.86%, and 53.28%, respectively (Table S2, Supporting Information). These results can potentially be explained by two factors: first, the crystallization‐induced emission characteristics of **TPABTBP**, **PMBTDP**, and **BTDPP** ^[^ ^19^ ^]^ and second, the effect of aggregate size.^[^ ^20^ ^]^ **TPABTBP**, **PMBTDP**, and **BTDPP** may undergo crystalline aggregates in low water fractions. However, when the water fraction exceeds 80%, these molecules tend to rapidly aggregate into smaller sizes with reduced emissive.^[^ ^21^ ^]^ The emission of small‐sized aggregates may be more susceptible to the influence of the surrounding solvent environment, resulting in a reduction in emission intensity.

To verify the presence of nanoaggregates in the DMSO/water, dynamic light scattering (DLS) experiments were performed. The DLS data shown in Figure S12 (Supporting Information) revealed that the size of the **TPABTBP** aggregates decreased from ≈196.3 nm at an 80 vol% water fraction to ≈146.4 nm at a 95 vol% water fraction. Similarly, when the water content in the system increased from 80% to 95%, the sizes of the **PMBTDP** and **BTDPP** aggregates reduced from 174.0 and 181.6 nm to 116.5 and 159.3 nm, respectively. We further measured the UV absorption spectra of **TPABTBP**, **PMBTDP**, and **BTDPP** in aqueous solutions (containing 5% DMSO). As shown in Figure S13 (Supporting Information), significant tailing phenomena were observed in the UV absorption spectra of **TPABTBP**, **PMBTDP**, and **BTDPP**. Specifically, **TPABTBP** and **BTDPP** showed distinct absorption peaks at 470  and 480 nm, respectively. The tailing phenomenon in the UV absorption spectra, induced by Mie scattering, further confirmed the formation of aggregates of **TPABTBP**, **PMBTDP**, and **BTDPP** in aqueous solutions. This observation is consistent with the results obtained from Dynamic Light Scattering (DLS) measurements. The results above indicate that in the mixed system of DMSO and water when the water content reaches 80%, the probes **TPABTBP**, **PMBTDP**, and **BTDPP** form aggregates, restricting intramolecular rotation and generating fluorescence. When the water content exceeds 80%, the decrease in fluorescence could possibly be attributed to a reduction in aggregate size. Generally, the larger the aggregate size, the brighter it is.^[^ ^22^ ^]^ Their polarity‐responsive and AIE properties make **TPABTBP**, **PMBTDP**, and **BTDPP** promising probes for LD imaging.

### 2.3 Specific Fluorescent Imaging of LDs in Live Cells

To verify the capacity of the probes (**PMBTDP**, **BTDPP**, and **TPABTBP**) for LD imaging in live cells, the colocalization experiments were performed using BODIPY 493/503, a commercial LD dye. As shown in Figure S14a–c (Supporting Information), the bright red fluorescence signals emitted by **PMBTDP**, **BTDPP**, and **TPABTBP** overlapped precisely with the green emission signals from BODIPY 493/503. Pearson's correlation coefficients were 0.93, 0.87, and 0.96 for **PMBTDP**, **BTDPP**, and **TPABTBP**, respectively, indicating that **PMBTDP**, **BTDPP**, and **TPABTBP** specifically targeted and stained LDs in living cells. The ClogPs of **PMBTDP** (ClogP = 8.65), **BTDPP** (ClogP = 7.21), and **TPABTBP** (ClogP = 7.16) were calculated using XlogP3^[^ ^23^ ^]^ and were significantly higher than the ClogP of BODIPY 493/503 (ClogP = 2.98). Because the LDs are less polar than certain other lipid structures, probes with higher ClogP values are better suited for LD imaging. Therefore, the appropriate lipophilic properties of **PMBTDP**, **BTDPP**, and **TPABTBP** contributed to their ability to target LD. Because **TPABTBP** showed weak fluorescence in polar solvents but strongly in nonpolar systems with emission wavelengths exceeding 600 nm, we selected **TPABTBP** for further research. To explore distractors in the organism and the effect of different pH on **TPABTBP**, we conducted an in vitro assessment of the interference caused by biomolecules (HSA, ATP, ADP, GSH, Cys, glucose and RNA), metal ions (Na^+^, K^+^, Fe^2+^, Fe^3+^, Ca^2+^), and pH on the fluorescence emission of the **TPABTBP**. As shown in Figure S15a,b (Supporting Information), the fluorescence intensity of **TPABTBP** mixed with various potential interferents did not exhibit significant changes. This observation indicates that **TPABTBP** possesses excellent anti‐interference capability in complex biological systems, making it advantageous for the specific imaging of LDs.

To determine the appropriate concentration of **TPABTBP** for LD imaging, HepG2 cells were subjected to bright‐field and fluorescence microscopy observations to evaluate the staining of LDs. Due to its higher refractive index, LDs are prominently visible as dark spots in phase‐contrast microscopy images. As shown in Figure S16 (Supporting Information), at a concentration of 5 µM, only a limited number of brightly fluorescent droplets, stained with **TPABTBP**, demonstrated a well‐pronounced merge with distinct black dots. In contrast, when the concentration of **TPABTBP** was increased to 30 µM, nearly all LDs in the cytoplasm were effectively stained with **TPABTBP**. Although 40 and 50 µM **TPABTBP** still provide good imaging results for LDs, considering the potential cytotoxicity caused by high concentrations of the probe and cost issues, we ultimately selected 30 µM as the final imaging concentration. Subsequently, we investigated whether concentration affects the co‐localization of **TPABTBP** with LDs. As demonstrated in Figure S17 (Supporting Information), after incubation of HeLa cells with various concentrations (5, 10, 30, 40, and 50 µM) of **TPABTBP**, robust fluorescent signals were detected in the red emission channel, and these signals closely overlapped those emitted by BODIPY 493/503. The Pearson's coefficients were 0.90, 0.90, 0.94, 0.91 and 0.88, respectively. Investigations into higher concentrations revealed no significant improvement in imaging quality. Based on the above results, we believe that 30 µM is the appropriate concentration for imaging LDs using **TPABTBP**. Additionally, we investigated the LD targeting efficiency of **TPABTBP** in various living cells, including H9c2 and RAW264.7 cells (Figure S18a,b, Supporting Information). Science the colocalization coefficients of **TPABTBP** and BODIPY 493/503 were 0.92, and their fluorescence signals closely overlapped, we concluded that **TPABTBP** effectively images LDs across different cell types.

**Figure 2:**
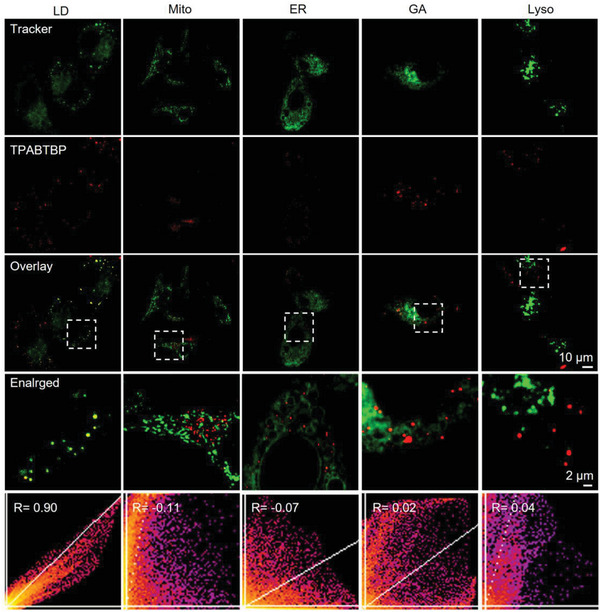
Colocalization of **TPABTBP** with Mito‐Tracker, ER‐Tracker, GA‐Tracker, and Lyso‐Tracker in HepG2 cells. The red fluorescence channel was used to visualize **TPABTBP** (*λ*_ex_/*λ*_em_ = 480/620 nm), while the green fluorescence channels were commercial probes: BODIPY 493/503 (*λ*_ex_/*λ*_em_ = 493/503 nm), Mito‐Tracker Green FM (*λ*_ex_/*λ*_em_ = 495/514 nm), ER‐TrackerGreen (*λ*_ex_/*λ*_em_ = 504/511 nm), NBD C6‐Ceramide‐BSA complex (*λ*_ex_/*λ*_em_ = 522/535 nm), and LysoTracker Red DND‐99 (*λ*_ex_/*λ*_em_ = 577/590 nm). The scatter plots showed the Pearson correlation coefficients of red and green channels. Scale bar: 10 µm. Magnified image: 2 µm.

To further confirm the specificity of **TPABTBP** for LD imaging, immunofluorescence colocalization analysis of the PLIN2 protein, which is highly expressed on the LD membrane, was conducted.^[^ ^24^ ^]^ As shown in Figure S19 (Supporting Information), the red fluorescence signal (indicating LD staining) overlapped well with the green fluorescence signal (representing PLIN2), demonstrating the superior specificity of **TPABTBP** for LD imaging. Additionally, the co‐localization of **TPABTBP** with other organelles was evaluated to confirm its labeling specificity for LDs. As depicted in **Figure** **2**, **TPABTBP** showed minimal co‐localization with commercial probes for mitochondrial (Mito), endoplasmic reticulum (ER), Golgi apparatus (GA), and lysosomal (Lyso). The Pearson coefficients between **TPABTBP** and Mito, ER, GA, and Lyso were −0.11, −0.07, 0.02, and 0.04, respectively. The morphology of LDs visualized by **TPABTBP** was distinctly clear and appeared nearly spherical. The red fluorescence signal generated by **TPABTBP** displayed excellent colocalization with the green fluorescence signal of BODIPY 493/503, with a high Pearson correlation coefficient of 0.9. These results collectively demonstrate the excellent specificity of **TPABTBP** for LD imaging.

### 2.4 Validating the Advantage of **TPABTBP** Relative to BODIPY493/503

Compared to the widely used BODIPY 493/503, **TPABTBP** offers three distinct advantages. Firstly, **TPABTBP** exhibits higher specificity for LD staining compared to BODIPY 493/503. We evaluated whether **TPABTBP** and BODIPY 493/503 exhibited non‐specific staining of lysosomes during imaging. As shown in Figure S20 (Supporting Information), there was a substantial overlap between the fluorescence signals of BODIPY 493/503 and Lyso‐Tracker, indicating potential non‐specific staining of lysosomes by BODIPY 493/503, consistent with previous findings.^[^ ^25^ ^]^ In contrast, the fluorescence signal of **TPABTBP** remained distinctly separate from the signal of lysosomes, confirming its excellent LD selectivity. Additionally, owing to its AIE characteristics, **TPABTBP** exhibits superior photostability compared to BODIPY 493/503. A photobleaching experiment conducted on HepG2 cells co‐incubated with BODIPY 493/503 and **TPABTBP** for 30 min revealed notable differences. As depicted in Figure S21a,b (Supporting Information), while the green fluorescence of BODIPY 493/503 rapidly decayed by 70% within 20 s under the same laser excitation power, **TPABTBP** only exhibited a 6% decrease. Even after continuous excitation for 240 s, the fluorescence intensity of BODIPY 493/503 decreased by ≈90%, whereas **TPABTBP** showed only ≈30% reduction, confirming its superior anti‐photobleaching properties. Moreover, the signal‐to‐noise ratio (SNR) of **TPABTBP** imaging of LDs surpassed that of BODIPY 493/503. SNR calculations conducted for both probes further corroborated their imaging performance. As shown in Figure S22a (Supporting Information), the red fluorescence signals of **TPABTBP** appeared clear and bright, while the green fluorescence signal from BODIPY 493/503 appeared diffuse and blurry throughout the cell. Analysis of ten randomly selected regions for SNR calculation indicated that the SNR of **TPABTBP** was 2.2 times higher than that of BODIPY 493/503 (Figure S22b, Supporting Information). This establishes a foundation for the application of **TPABTBP** to explore the dynamics of LDs. These results demonstrate that compared to the commercial LD probe BODIPY 493/503, **TPABTBP** possesses superior LD imaging capabilities.

### 2.5 Molecular Dynamics (MD) Simulation of the Interaction Between the Probe and LDs

To assess **TPABTBP**’s ability to penetrate the phospholipid monolayer membrane of LDs, we conducted all‐atom molecular dynamics (MD) simulations to track **TPABTBP** migration from the cytosol to the LD core. The construction of the simulated LD system involved three primary steps: 1) constructing the phospholipid membrane system; 2) creating the triacylglycerol (TAG) slab; and 3) inserting the TAG layer between the upper and lower phospholipid monolayers to form the complete simulation system.

Firstly, a solvated phospholipid bilayer (≈ 2 nm thick) was constructed and equilibrated in an isothermal‐isobaric (NPT) ensemble for 100 ns. As depicted in Figure S23 (Supporting Information), after 100 ns of equilibration, the phospholipid membrane system exhibited convergence in terms of the surface area per lipid molecule (APL), phospholipid density, energy of the phospholipid membrane system, and temperature of the simulation system. This convergence indicated that the phospholipid membrane had reached an equilibrium state. Secondly, we used Packmol software to build TAG Slab (10 nm‐thick). Prior to assembling the complete simulation system, a separate 20 ns NPT simulation was conducted on the TAG slab under conditions of 300 K and 1 bar to attain an equilibrium state. After 20 ns of equilibration, the energy of the system and the density of TAG within the TAG slab stabilized, indicating convergence (Figure S24, Supporting Information). Finally, we inserted the equilibrated TAG slab between the upper and lower phospholipid monolayers and conducted a 10 ns NPT equilibration simulation of the combined system. After 10 ns of equilibrium simulation, the system's density, energy, and surface area per lipid molecule exhibited stabilization, indicating that the LD simulation system had reached a stable and converged state (Figure S25, Supporting Information).

**Figure 3:**
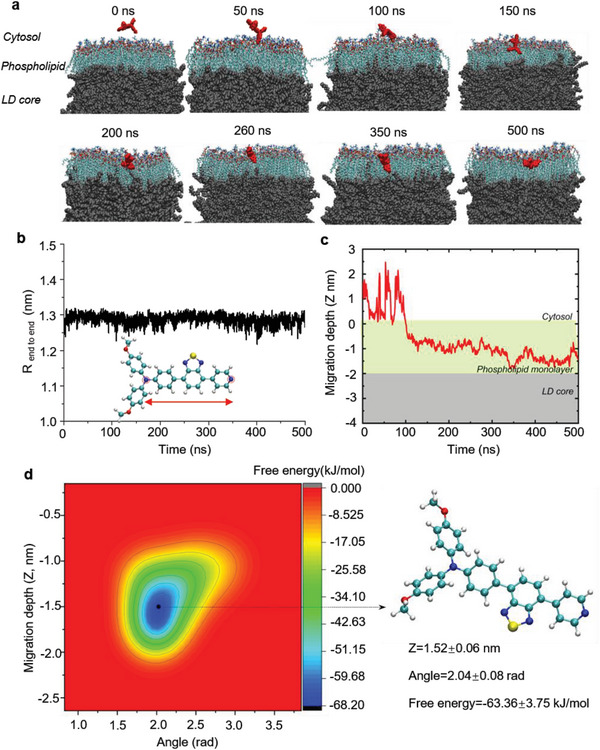
Molecular dynamics (MD) simulation of probe **TPABTBP** migration across the cytosol to the LD phospholipid monolayer. a) Representative snapshots showing **TPABTBP** migration to the phospholipid monolayer during a 500 ns simulation. b) End‐to‐end **TPABTBP** migration distance (*R*_end‐to‐end_). c) The position of the migrating **TPABTBP** in relation to the phospholipid monolayer was determined, with the phospholipid headgroup fixed at *z* = 0 and the monolayer range highlighted in yellow‐green. d) The free energy landscape of **TPABTBP** migration into LDs was determined using multi‐walker well‐tempered meta‐dynamics simulations, and the representative structures are illustrated in the inset figures, with each image linked to the migration depth and angle (between the *R*_end to end_ vector of **TPABTBP** and the membrane normal).

Our LD model comprised two interfaces: one at the interface between the cytosol and phospholipid monolayer (interface I), and the other at the boundary between the phospholipid monolayer and TAGs (interface II). Through unrestricted MD simulations and meta‐dynamics calculations, we investigated **TPABTBP**’s migration process across these boundaries. At 100 ns, **TPABTBP** began to cross interface I and had completely entered the phospholipid monolayer by 200 ns (**Figure** **3**a). However, **TPABTBP** was trapped in the phospholipid monolayer during the 200–500 ns simulation and failed to pass interface II. We quantified **TPABTBP**’s end‐to‐end distance (*R*_end‐to‐end_) to investigate its structural changes during LD migration, revealing a constant probe length of ≈1.3 nm, indicating rigidity (Figure 3b). The migration depth of **TPABTBP** relative to the phospholipid monolayer showed rapid infiltration upon reaching interface I, with subsequent stalling within the monolayer during the 500‐ns stimulation (Figure 3c). Owing to the rigid structural characteristics of **TPABTBP**, the variation conformational order parameter (µ) was independent of the *R*_end‐to‐end_ and solely reflected alteration changes (Figure S26, Supporting Information). To better assess **TPABTBP**’s orientation, we measured the angle between the *R*_end‐to‐end_ vector and the membrane normal (Figure S27, Supporting Information). The rigid structure of **TPABTBP** likely creates a substantial free energy barrier during penetration into the LD core, as geometric rearrangements are required to accommodate the unfolded probe within interlaced TAG chains. Multi‐walker well‐tempered meta‐dynamics simulations with four replica walkers were conducted to obtain the free energy landscape when **TPABTBP** crossed the two interfaces.^[^ ^26^ ^]^ The separate replicas are shown in Figures S28 and S29 (Supporting Information). As shown in Figure 3d, **TPABTBP** exhibited the lowest free energy (−63.36 ± 3.75 kJ mol^−1^) at a maximum migration depth of −1.52 ± 0.05 nm, corresponding to an angle of 2.04 ± 0.08 radians between the *R*_end‐to‐end_ vector and the membrane normal. This finding confirmed that **TPABTBP** cannot migrate to the core of LDs, localizing instead to the phospholipid monolayer membrane. The MD simulation revealed that **TPABTBP** localized to the monolayer phospholipid membrane structure of LDs. The low‐polarity environment of the phospholipid monolayer promotes the fluorescence emission of **TPABTBP**. Additionally, the parallel arrangement of long phospholipid chains in the monolayer helps restrict the intramolecular rotation of **TPABTBP**, further enhancing fluorescence emission. Consistent with previous findings, our results suggested that rigid lipid molecules such as **TPABTBP** cannot cross the LD phospholipid monolayer membrane to enter the lipid core.^[^ ^27^ ^]^

### 2.6 Experimentally Validate that **TPABTBP** Specifically Localized to the Phospholipid Monolayer Membrane of LDs

To validate the specific targeting of **TPABTBP** to the lipid monolayers on LDs, we conducted a series of experiments employing **TPABTBP** for imaging LDs. Initially, LDs were extracted from HepG2 cells utilizing an LD isolation kit and then incubated with **TPABTBP** at room temperature for 3 min prior to microscopic observation. In Figure S30 (Supporting Information), LDs are characterized as red hollow circular structures.

Further examination involved magnifying LDs with a 100x oil objective during laser confocal microscopy imaging of HepG2 cells, where **TPABTBP** appeared as red rings surrounding the LDs, as illustrated in Figure S31 (Supporting Information). This pattern was consistently observed in H9c2 cells under identical magnification, reinforcing the membrane‐targeting capabilities of **TPABTBP**. To capture the dynamics of this process, in‐situ time‐lapse confocal imaging was employed, with Video S1 (Supporting Information) demonstrating the distinctive red, hollow, ring‐shaped patterns of LDs in the cytoplasm, confirming **TPABTBP**’s specific targeting. Moreover, a HepG2 cell line engineered to express GFP‐tagged PLIN2,^[^ ^28^ ^]^ a protein associated with the LD membrane, was used. Following oleic acid (OA) treatment to induce LD formation, these cells were stained with **TPABTBP** and analyzed under 1000x magnification using fluorescence microscopy. Figure S32 (Supporting Information) shows the colocalization of GFP‐PLIN2's green fluorescence with **TPABTBP**’s red fluorescence, particularly notable at higher magnifications, suggesting a specific association of **TPABTBP** with the LD membrane.

**Figure 4:**
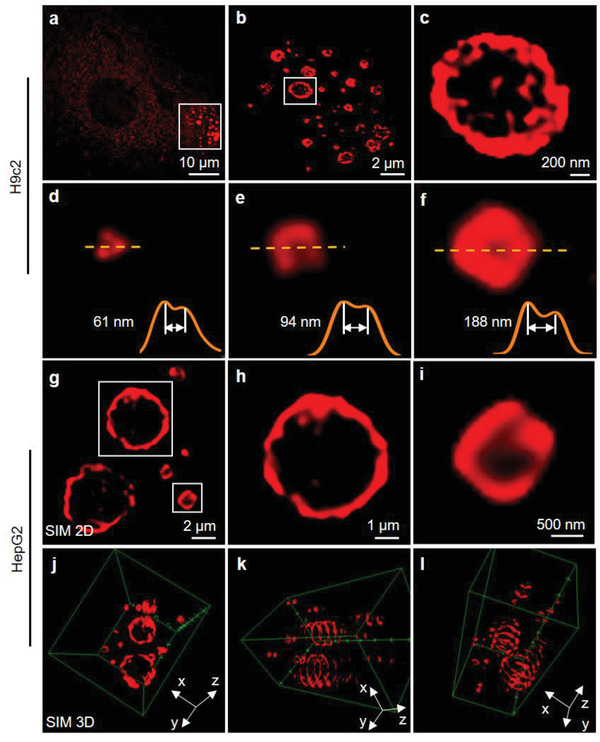
Structured illumination microscopy (SIM) imaging of LDs in live cells. a) SIM images of H9c2 cells stained with 30 µM **TPABTBP** for 0.5 h. b,c) Corresponding enlarged SIM images at different scales (2 µm, 200 nm). d–f) Representative images and fluorescence intensity profiles of the LDs by SIM^2^ (white arrow: diffraction limit). g) SIM images of LDs in HepG2 cells stained with 30 µM **TPABTBP** for 0.5 h. Scale bar: 2 µm. Corresponding enlarged SIM images h, i) at different scales (1 µm, 500 nm). j–l) Reconstruction of *z*‐axis images from SIM viewed from different angles.

Further insights were gained through structured illumination microscopy (SIM). H9c2 cells were incubated with 30 µM **TPABTBP** for 30 min to stain LDs. As shown in **Figure** **4**a, SIM images showed the distribution of spherical LDs in the cytoplasm. By increasing the magnification of the image, the ring‐shaped red fluorescence pattern of the LDs is clearly seen, indicating that **TPABTBP** was localized mainly on the LD monolayer membrane (Figure 4b,c). Due to the excellent spatial resolution of SIM imaging, the diffraction limit of **TPABTBP** in imaging LDs reached 61 nm (Figure 4d). We also observed similar hollow rings in other LDs (Figure 4e,f). Especially, the spectral analysis of the selected LD signals revealed a decrease in the red fluorescence signal of **TPABTBP** at the core of the LDs, while it was enhanced at the LD membrane. These results indicate the binding of **TPABTBP** to the monolayer phospholipid membrane of LDs, and it was consistent with the MD simulations. We further assessed the target specificity of **TPABTBP** toward LD membranes using cells with large LDs, such as HepG2 cells known for their active lipid metabolism.^[^ ^29^ ^]^ As shown in Figure 4g–i, LDs exhibited a distinctive hollow ring‐like structure. Notably, after a layer‐by‐layer scan of LD structures using SIM, 3D structural fitting images unequivocally demonstrated that, from a different rotational perspective, **TPABTBP** signals were localized on the LD membrane (Figure 4j–l). These findings provide further confirmation of **TPABTBP**’s specific targeting of the monolayer phospholipid membrane of LDs.

### 2.7 Imaging the Dynamic Changes in LDs During Ferroptosis

**Figure 5:**
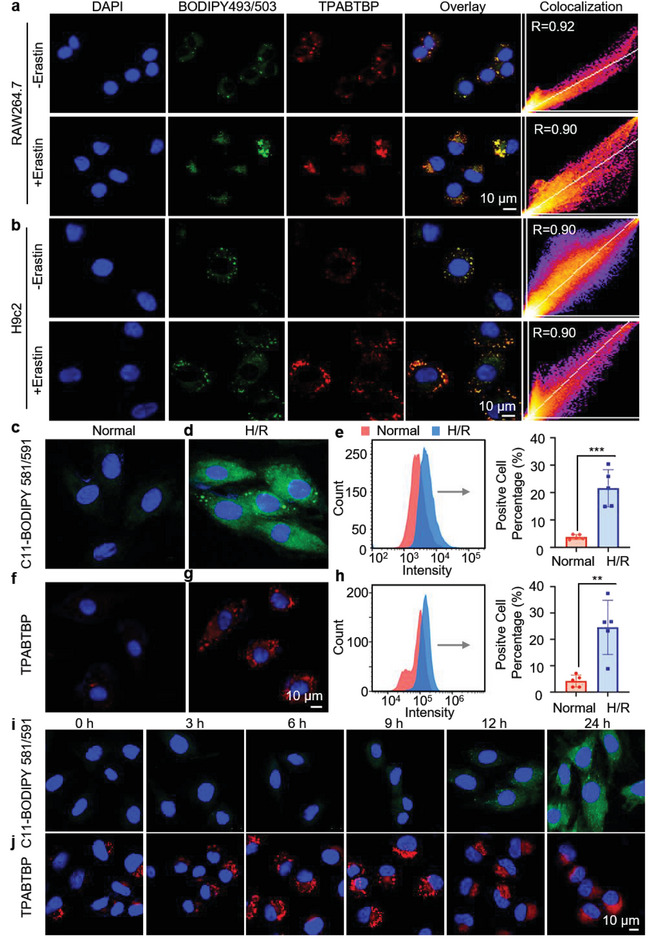
The visualization of LDs during ferroptosis. a,b) CLSM images of RAW264.7 and H9c2 cells treated with Erastin (5 µM) and scatter plots showing the Pearson correlation coefficients of cells cocultured with **TPABTBP** (30 µM) and BODIPY 493/503 (5 µM). Scale: 10 µm. c,d) CLSM images of H9c2 cells subjected to hypoxia for 12 h, reoxygenation for 2 h, and incubation with C11‐BODIPY581/591 (5 µM). e) Corresponding flow analysis of H9c2 cells subjected to hypoxia/reoxygenation (H/R) and then cultured with C11‐BODIPY581/591 (5 µM). f, g) CLSM images of H9c2 cells cultured under H/R conditions and labeled with **TPABTBP** (30 µM). h) Flow cytometry analysis of H9c2 cells cultured with H/R and labeled with **TPABTBP** (30 µM). i, j) The LD numbers and lipid peroxide levels of H9c2 cells treated with 5 µM Erastin as observed by CLSM at various time points (0, 3, 6, 9, 12, and 24 h). The green channel represents C11‐BODIPY581/591 (5 µM), while the red channel represents **TPABTBP** (30 µM). Scale: 10 µm; *n =* 5. Statistical significance is reported as *p < 0.05; **p < 0.01; ***p < 0.001, as determined by Student's t‐test, in panels e through h.

Recent studies have highlighted a close relationship between myocardial ischemia‐reperfusion injury (MIRI) and ferroptosis.^[^ ^30^ ^]^ Although ferroptosis is driven by lipid peroxidation,^[^ ^5^, ^31^ ^]^ the impact of LDs on the dynamic progression of ferroptosis remains unclear. To investigate ferroptosis, we treated H9c2 and RAW264.7 cells with Erastin, a well‐known inducer of ferroptosis, for 24 h, followed by co‐staining with **TPABTBP** and BODIPY 493/503. In the absence of Erastin, LDs were observed as punctate structures distributed throughout the cytoplasm of both RAW 264.7 and H9c2 cells. However, upon Erastin treatment (5 µM), there was a significant increase in both the number and mean diameter of LDs in both cell types (**Figure** **5**a,b). To provide quantitative evidence for this phenomenon, we quantified **TPABTBP** fluorescence intensity in RAW 264.7 and H9c2 cells. In comparison to the control group, after induction for 24 h, the mean fluorescence intensity (MFI) in RAW264.7 and H9c2 cells were 2.0 and 2.8 times higher, respectively (Figure S33a,b, Supporting Information). The increase in the number of LDs during ferroptosis indicated that LDs may have antioxidative functions, and they store polyunsaturated fatty acids to protect cells from lipotoxicity and oxidative stress.^[^ ^32^ ^]^

To mimic MIRI in vitro, H9c2 cells were subjected to induce hypoxia/reoxygenation (H/R) injury.^[^ ^33^ ^]^ C11‐BODIPY 581/591 was utilized to assess the extent of lipid peroxidation in H9c2 cells during the H/R process. Lipid peroxidation results in a transition of the fluorescence emission peak of C11‐BODIPY 581/591 from red light (≈ 590 nm) in the reduced state to green light (≈510 nm) in the oxidized state. H9c2 cells in the normal group exhibited strong red fluorescence with weak green fluorescence, indicating low levels of lipid peroxidation (Figure S34, Supporting Information; Figure 5c). However, after 12 h of hypoxia followed by 2 h of reoxygenation, the MFI of the green channel increased significantly, while the MFI of the red channel decreased markedly by 75.97%, indicating a notable increase in lipid peroxidation (Figure S34c, Supporting Information; Figure 5d). Flow cytometry analysis showed that the lipid peroxidation rate of cardiomyocytes in the H/R treatment group was 5.7‐fold (21.58% vs. 3.77%) higher than that of the normoxia group (Figure 5e). Subsequently, we have performed additional experiments using the Calcein‐AM/PI cytotoxicity assay kit to evaluate cell death under conditions of hypoxia‐reoxygenation (H/R) and Erastin induction. The Calcein‐AM/PI assay enables the discrimination between live and dead cells, with Calcein‐AM emitting intense green fluorescence in live cells, and PI labeling dead cells with red fluorescence. In Figure S35a (Supporting Information), the red fluorescence signal of H9c2 cells significantly increased after 12 h of hypoxia followed by 2 h of reoxygenation compared to the Normal group, indicating an elevated number of dead cells. The quantitative results of live cells labeled with Calcein‐AM indicated that the proportion of live cells in the H/R group decreased to 27.4% (Figure S35b, Supporting Information). These results indicated that simulating ferroptosis in vitro through hypoxia‐reoxygenation indeed leads to a substantial increase in cell death.

Additionally, we used the probe **TPABTBP** to explore how LDs in cardiomyocytes respond to H/R treatment. As illustrated in Figure 5f,g, the number of LDs was significantly increased after H/R treatment. This change aligned with the changes in RAW 264.7 and H9c2 cells when Erastin was used to induce ferroptosis. A quantitative flow cytometry analysis showed that LD content in H9c2 cells after H/R treatment was 5.8‐fold that of the normoxia group (Figure 5h). These findings reveal that H/R treatment induces ferroptosis in living cells, and this process was accompanied by a marked increase in the number of LDs in the cytoplasm.

To explore LD dynamics during ferroptosis, we induced Erastin in HepG2, H9c2, and Hela cells, and then monitored LD changes at various time points through imaging. As depicted in Figure S36a,b (Supporting Information), the quantity of LDs increased during the early stages of ferroptosis induction (0–9 h), followed by a decline during the later stages (12–24 h). In order to reveal the correlation between the trends in LDs and intracellular lipid peroxidation levels, we further investigated the time‐dependent changes in LDs and lipid peroxidation during ferroptosis. H9c2 cells were treated with 5 µM Erastin at various time points (0, 3, 6, 9, 12, and 24 h), labeled with Oil Red O, and treated with C11‐BODIPY 581/591 and **TPABTBP**. The results indicated that with the prolonged induction time of Erastin, the intracellular lipid peroxidation levels continued to increase (Figure 5i; Figure S37, Supporting Information), while LDs exhibited a trend of initially increasing (0–9 h) and then decreasing (Figure 5j; Figure S38, Supporting Information). Furthermore, Erastin‐induced ferroptosis in H9c2 cells was monitored for changes in cell death using Calcein‐AM/PI staining. As depicted in Figure S39a (Supporting Information), the PI fluorescence signal within the cells exhibited a progressive enhancement after 3 h of Erastin induction, reaching a point at 24 h where the majority of cells were labeled with red PI fluorescence. Meanwhile, the Calcein‐AM fluorescence signal, indicative of live cells, remained exceedingly faint. These observations suggested a notable escalation in the cell death rate with increasing duration of Erastin exposure. Moreover, the proportion of viable cells was quantified by assessing the variations in Calcein‐AM fluorescence intensity. The results revealed that after 24 h of Erastin induction, the ratio of live H9c2 cells significantly decreased to only 8.46% (Figure S39b, Supporting Information). Thus, these findings demonstrated that cell death is specific to hypoxia‐reoxygenation (H/R) and Erastin‐induced ferroptosis. In addition, we used flow cytometry to quantify the number of LDs and the level of lipid peroxidation. As depicted in Figure S40 (Supporting Information), the number of LDs continuously increased from 0 to 9 h, peaking at 9 h when the number of LDs was 5.9 times that of the 0‐h group. However, as the induction time with Erastin increased to 12 h, the number of LDs began to decrease. The timing of the changes in LD number in the cytoplasm was completely different from that of the changes in lipid peroxidation level. The level of lipid peroxidation further increased with increasing Erastin treatment time, indicating exacerbated ferroptosis. These results indicated that LD synthesis increases in the early stage, but LDs begin to breakdown in the late stage of ferroptosis.

LDs are not static organelles but increase/decrease in numbers influenced by a long list of biological stimuli such as hormones, energy substrate availability, and hypoxia/reperfusion. Therefore, we used H9c2 and HepG2 cells with different levels of LDs to test the involvement of LDs in Erastin‐induced ferroptosis. As a monounsaturated fatty acid, OA was widely used to promote LD synthesis in living cells.^[^ ^34^ ^]^ Therefore, H9c2 and HepG2 cells were pre‐treated with 100 µM OA for 16 h. Untreated H9c2 and HepG2 cells were used as controls. Subsequently, we assessed LD content and the degree of lipid peroxidation by using **TPABTBP** and C11‐BODIPY during Erastin‐induced ferroptosis.

After OA treatment, both H9c2 and HepG2 cells exhibited a significant increase in LD content (Figure S41a,e, Supporting Information). Specifically, LD content increased ≈2.3‐fold in H9c2 cells and 3.1‐fold in HepG2 cells compared to untreated cells (Figure S41b,f, Supporting Information). However, the levels of lipid peroxidation in both cell lines did not show a significant difference with or without OA pretreatment (Figure S41d,h, Supporting Information). After 9 h of Erastin induction, there was a significant increase in LD content in both H9c2 and HepG2 cells (Figure S41a,e, Supporting Information). In H9c2 and HepG2 cells without OA pretreatment, the LD content increased by 9.6 and 15.9‐fold, respectively (Figure S41b,f, Supporting Information). In OA‐pre‐treated H9c2 and HepG2 cells, the LD content increased by 9.8 and 7.2‐fold, respectively (Figure S41b,f, Supporting Information). After 9 h of Erastin induction, lipid peroxidation levels increased in H9c2 and HepG2 cells but remained at relatively lower levels (Figure S41c,g, Supporting Information). However, compared to the 9 h time point, after 20 h of Erastin induction, the LD content decreased by 77.35% and 86.96% in H9c2 and HepG2 cells without OA pretreatment (Figure S41b,f, Supporting Information), while lipid peroxidation levels increased by 3.9 and 2.9‐fold, respectively (Figure S41d,h, Supporting Information). Similarly, in OA‐pretreated H9c2 and HepG2 cells, the LD content decreased by 68.72% and 78.27%, respectively (Figure S41b,f, Supporting Information), and the lipid peroxidation levels increased by 3.4‐ and 2.2‐fold, respectively (Figure S41d,h, Supporting Information). Interestingly, after 20 h of Erastin induction, cells pretreated with OA exhibited lower levels of lipid peroxidation compared to untreated cells. This observation may be attributed to OA's ability to suppress ferroptosis by reducing the quantity or density of easily oxidizable polyunsaturated fatty acids in the cell membrane.^[^ ^34^, ^35^ ^]^ These results suggest that during ferroptosis, there is an initial increase in cellular LDs followed by a subsequent decrease, irrespective of the initial LD content. In the early stages, cells synthesize new LDs to store free fatty acids as a defense mechanism against lipid peroxidation.^[^ ^31a^ ^]^ However, in the later stages, the breakdown of LDs may release fatty acids, promoting cellular ferroptosis.^[^ ^36^ ^]^

### 2.8 LDs are Degraded via the Lipophagy Pathway, and Inhibiting LD Breakdown Alleviates Ferroptosis

**Figure 6:**
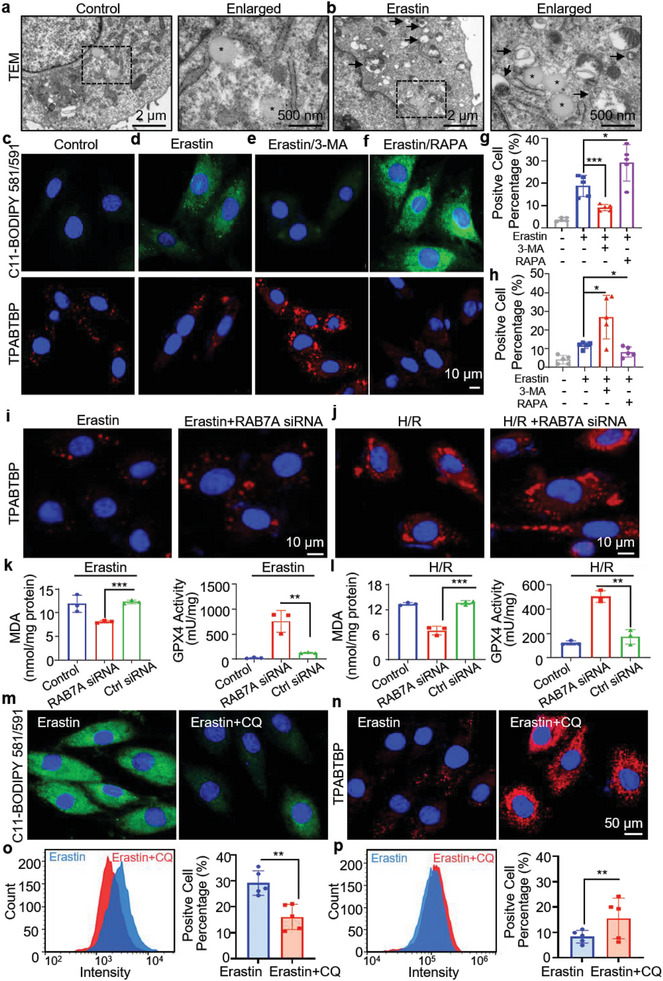
Detection of LDs and ferroptosis in H9c2 cells after inhibition of lipophagy. a,b) Transmission electron microscopy (TEM) images of H9c2 cells treated with 5 µM Erastin for 24 h (black arrows: autophagic vacuole; *: LD). Lipid peroxidation level and LD content in H9c2 cells were divided into four groups: the c) untreated group, the d) Erastin‐induced group, the e) Erastin + 3‐MA treated group, and the f) Erastin + RAPA treated group. Green and red channels correspond to lipid peroxides and LDs. Reagents: 5 µM Erastin; 10 mM 3‐MA; 100 nM RAPA. Scale: 10 µm. Quantification of g) lipid peroxidation levels and h) LD content in H9c2 cells using flow cytometry. i) After induction with 5 µM Erastin for 20 h, the LD content in norma and RAB7A‐knockdown H9c2 cells were evaluated. j) Assessment of LD content in normal and RAB7A knockdown H9c2 cells under H/R conditions. k, l) The intracellular levels of MDA and GPX4 in three types of H9c2 cells (normal H9c2 cells, RAB7A‐knockdown H9c2 cells, and Control siRNA‐treated H9c2 cells) were quantified after induction with 5 µM Erastin for 20 h or under H/R conditions. Confocal images were used to measure m) lipid peroxide levels and n) LD content in H9c2 cells treated with culture medium containing Erastin (5 µM) or Erastin +CQ (5 µM) for 20 h. Scale: 50 µm. o, p) The proportion of cells stained with C11‐BODIPY 581/591 or **TPABTBP** in H9c2 cells were analyzed by flow cytometry. Student's t‐test to compare statistical differences; *p < 0.05; **p < 0.01; ***p < 0.001.

Previous studies have established the involvement of LD breakdown primarily through lipophagy and lipolysis.^[^ ^9^, ^37^ ^]^ To visualize this process, we utilized transmission electron microscopy (TEM) to examine variations in LD morphology and structure in H9c2 cells before and after Erastin treatment. As shown in **Figure** **6**a, in the absence of Erastin, the LDs in H9c2 cells exhibited a regular shape and smooth surface, with an average size of 0.4 µm. However, after treatment with Erastin (5 µM) for 24 h, the number of LDs in the cytoplasm increased, exhibiting irregular shapes and sizes. Notably, many LDs were closely associated with autophagic vesicles, indicating the involvement of autophagy in LD breakdown during ferroptosis (Figure 6b).

To validate the essential role of the autophagic machinery in these processes, we examined the autophagic flux following induction with 3‐methyladenine (3‐MA) or rapamycin (RAPA) during Erastin‐induced ferroptosis in H9c2 cells. The mCherry‐eGFP‐LC3 adenovirus was employed for monitoring alterations at various phases of the autophagy process. When the autophagosome fuses with lysosome, the green fluorescence signal originating from eGFP dissipates owing to the sensitivity of eGFP to acidic conditions.^[^ ^38^ ^]^ Thus, the yellow‐fluorescent and red‐fluorescent puncta represent the formation of autophagosomes and autolysosomes, respectively (Figure S42a, Supporting Information). As shown in Figure S42b,c (Supporting Information), distinct red fluorescence puncta (autolysosomes) were clearly visible, indicating the occurrence of autophagy accompanying Erastin‐induced ferroptosis. In the Erastin/3‐MA group, both the number of autophagosomes (yellow puncta) and autolysosomes (red puncta) decreased, indicating suppressed autophagic flux (Figure S42d,e, Supporting Information). This is attributed to 3‐MA blocking class III PI‐3K and inhibiting autophagophore formation.^[^ ^39^ ^]^ Conversely, in the Erastin/RAPA group, the number of autolysosomes significantly increased (Figure S42f,g, Supporting Information), reflecting unimpeded autophagy due to RAPA inhibiting the mTOR pathway and enhancing autophagosome formation.^[^ ^38^ ^]^ Overall, these findings indicated that 3‐MA inhibits autophagic flux, while RAPA promotes it during Erastin‐induced ferroptosis. In addition, we generated ATG5 gene‐deficient cells to provide additional confirmation of the essential role of autophagy in LD breakdown. The selection of ATG5 gene for knockout was based on its pivotal function in orchestrating the elongation of cell membranes within autophagosomes, establishing its significance as a key autophagy‐related gene.^[^ ^40^ ^]^ Firstly, the successful deletion of the ATG5 gene was confirmed through PCR and Sanger sequencing (Tables S3 and S4 and Figure S43, Supporting Information). Subsequently, we assessed the autophagy flux in ATG5 KO H9c2 cells, which are unable to form autophagophores. As shown in Figure S44 (Supporting Information), we observed an increase in green fluorescence signal in ATG5 KO cells during Erastin induction compared to wild‐type (WT) H9c2 cells. Additionally, the fluorescent puncta content of autolysosomes in ATG5 KO cells exhibited a notably reduced level compared to the WT group after Erastin induction. These findings suggest a significant impediment in autophagic vesicle formation, ultimately inhibiting autophagy.

Next, we investigated how autophagy influences the content of LDs and lipid peroxidation levels during ferroptosis. H9c2 cells were treated with 3‐MA /RAPA and Erastin for 20 h followed by a 30‐min incubation with C11‐BODIPY and **TPABTBP**. As shown in Figure 6c,d, H9c2 cells pretreated with Erastin exhibited a significant increase in lipid peroxidation level and LD content compared to control, indicating the occurrence of ferroptosis. In the presence of both 3‐MA and Erastin, the intensity of C11‐BODIPY 581/591(representative of lipid peroxidation levels) was significantly reduced compared to the Erastin‐induced group (Figure 6d,e). Flow cytometry quantification confirmed a 51.5% decrease in lipid peroxidation levels (Figure 6g). Conversely, the cellular LD content increased by 2.3‐fold (Figure 6h). These results indicate that inhibiting autophagy alleviates Erastin‐induced ferroptosis. As depicted in Figure 6d,f, it is evident that the green fluorescence intensity of C11‐BODIPY 581/591 is significantly increased in the Erastin+RAPA group. Flow cytometry quantification results indicate a 1.6‐fold increase in lipid peroxidation levels compared to the Erastin group (Figure 6g). On the contrary, in the Erastin+RAPA group, the red fluorescence intensity of **TPABTBP** (representing LDs) decreased, and quantitative results showed a 29.92% reduction in LD content compared to the Erastin group (Figure 6h). This result can be attributed to RAPA's role as an autophagy inducer, facilitating the breakdown of LDs and exacerbating lipid peroxidation.^[^ ^9^, ^38^ ^]^ The above results provide preliminary evidence that inhibiting autophagy reduces LD degradation and alleviates the severity of ferroptosis, whereas promoting autophagy has the opposite effect.

To further confirm the key role of autophagy in regulating LDs and ferroptosis, we monitored LDs and lipid peroxidation levels in WT and ATG5 KO cells after inducing them with Erastin for 20 h. As depicted in Figure S45a (Supporting Information), LDs in ATG5 KO cells significantly increased compared to the WT group after Erastin induction. Quantitative analysis of the average fluorescence intensity in the images revealed a 3.7‐fold increase in the red fluorescence intensity of **TPABTBP** (Figure S45b, Supporting Information). In contrast, the green fluorescence intensity of C11‐BODIPY decreased by 80.64% (Figure S45c,d, Supporting Information). These results confirm that LDs are primarily degraded through the autophagy pathway during Erastin‐induced ferroptosis. Inhibiting autophagy can impede LD degradation, thereby alleviating lipid peroxidation. However, it is currently unclear which autophagy regulates LD degradation during ischemia/reperfusion‐induced cardiomyocyte ferroptosis.

Lipophagy is a distinct type of autophagy, characterized by the fusion of LDs with lysosomes.^[^ ^9^, ^41^ ^]^ Therefore, we employed **TPABTBP** and BODIPY 493/503 to monitor lipophagy following Erastin pretreatment. As shown in Figure S46 (Supporting Information), in both the 0‐h and 9‐h groups, the red signal (representing LDs) did not overlap with the green signal (representing lysosomes), indicating that the process of lysosomal engulfment of LDs had not yet occurred. However, after 16 h of Erastin induction, some overlap between Lyso and LDs became visible. After 20 h, lysosomes and LDs almost entirely overlapped. These findings validate the occurrence of LD degradation during ferroptosis via the process of lipophagy. We further performed similar validation experiments using BODIPY 493/503 to label LDs (Figure S47, Supporting Information). Unfortunately, in the 0‐h, 9‐h, and 20‐h groups, there was some co‐localization between BODIPY 493/503 and LysoTracker, likely due to non‐specific staining behavior. These findings confirm that LDs are degraded through lipophagy during ferroptosis, and highlight the superior specificity of **TPABTBP** in imaging LDs compared to BODIPY 493/503.

The lipophagy of LDs depends on RAB7A, a member of the RAS oncogene family.^[^ ^9b^ ^]^ Therefore, we further investigated whether RAB7A knockdown reduces LD breakdown and suppresses ferroptosis in H9c2 cells (Figure S48, Supporting Information). Polymerase Chain Reaction (PCR) (Figure S49, Supporting Information) and Western blot (Figure S50, Supporting Information) analysis proved that the RAB7A was downregulated in H9c2 cells. As shown in Figure S51 (Supporting Information), the control (Ctrl) siRNA did not significantly affect the number of LDs in RAB7A‐knockdown cells. Subsequently, we investigated whether knocking down RAB7A affects lipophagy, thereby regulating LD content and ferroptosis. As shown in Figure S52 (Supporting Information), In the Erastin+RAB7A siRNA group, the red fluorescence signal of LDs and the green fluorescence signal of lysosomes were significantly separated compared to the Erastin‐treated group, indicating a marked reduction in their co‐localization. These results strongly confirm the occurrence of lipophagy during Erastin‐induced ferroptosis in H9c2 cells and demonstrate that downregulating RAB7A effectively inhibits lipophagy. Additionally, confocal FI of **TPABTBP** showed that compared with normal H9c2 cells treated by Erastin or subjected to H/R, the number of LDs in H9c2 cells with the RAB7A gene knocked down was significantly increased (Figure 6i,j). As shown in Figure S53, upon treatment with Erastin, the MFI of RAB7A‐knockdown H9c2 cells was 1.95 times higher than the control group. Moreover, under hypoxia/reoxygenation conditions, the MFI in RAB7A knockdown H9c2 cells exhibited a 1.42‐fold increase compared to the control group. This phenomenon indicates that inhibiting the lipophagy pathway can prevent the breakdown of LDs.

We then examined the impact of RAB7A silencing on lipid peroxidation levels under pro‐ferroptotic conditions. As shown in Figure S54a (Supporting Information), after 20 h of Erastin induction in H9c2 cells with RAB7A knockdown, lipid peroxidation levels were notably lower compared to cells with normal RAB7A expression. Quantitative analysis showed a 27.2‐fold increase in the green fluorescence intensity of C11‐BODIPY after Erastin induction, while the fluorescence intensity decreased by 73.38% in the RAB7A knockdown group compared to the normal RAB7A expression group (Figure S54b, Supporting Information). Additionally, RAB7A knockdown reduced the MDA content by 34% and increased the GPX4 content by 5.8‐fold compared to the control siRNA group (Figure 6k). Similarly, under H/R conditions, RAB7A knockdown led to a 48.8% reduction in MDA content and a 4.1‐fold increase in GPX4 content compared to the control siRNA group (Figure 6l). These findings confirm that inhibiting lipophagy effectively reduces lipid peroxide levels, offering a potential new strategy for mitigating ferroptosis. However, it is worth noting that there are currently no commercially available inhibitors specifically targeting lipophagy.

To validate our findings, we treated Erastin‐induced H9c2 cells with chloroquine (CQ), a well‐established autophagy inhibitor,^[^ ^42^ ^]^ for 24 h, and then assessed the autophagy flux. As shown in Figure S55 (Supporting Information), in the Erastin/CQ group, there was a significant increase in the number of yellow fluorescent puncta (autophagosomes), while the number of red fluorescent puncta (autolysosomes) markedly decreased. This indicated that CQ disrupted the fusion between autophagosomes and lysosomes, thereby impeding autophagy at the stage of autophagosome formation.^[^ ^39^ ^]^ Subsequently, the cells were incubated with C11‐BODIPY or **TPABTBP** to assess the extent of lipid peroxidation and the content of LDs (Figure S56, Supporting Information). Upon addition of CQ to Erastin‐treated H9c2 cells, the intensity of green fluorescence emitted by C11‐BODIPY significantly decreased (m). Flow cytometry analysis further demonstrated that the lipid peroxidation level in cells following CQ treatment decreased from 29.16% to 16.1% compared to the Erastin‐treated group (o). However, under the same conditions, the LD count exhibited a contrasting trend. In the presence of CQ, Erastin‐treated H9c2 cells showed a notable increase in cytoplasmic LDs (Figure 6n), as confirmed by flow cytometry, with LD count being 1.9‐fold higher compared to the Erastin group (Figure 6p). Taken together, these findings suggest that CQ inhibits LD degradation and protects cardiomyocytes from lipid peroxidation‐induced damage.

### 2.9 Evaluation of the Biosafety of the Probe **TPABTBP**

We conducted comprehensive assessments to evaluate the safety profile of **TPABTBP** both in vitro and in vivo. Initially, we examined the cytotoxic effects of **TPABTBP** on H9c2 cells across a range of concentrations (5, 10, 30, and 120 µM) using CCK8 assays. Remarkably, **TPABTBP** exhibited no discernible toxicity even at the highest concentration tested (120 µM), as evidenced by the cell viability data (Figure S57, Supporting Information). Subsequently, we investigated the impact of **TPABTBP** on red blood cells (RBCs) via hemolysis assays. RBCs isolated from C57BL/6J mice were exposed to various concentrations of **TPABTBP** (0, 5, 10, 30, and 120 µM), along with Triton X‐100^[^ ^43^ ^]^ as a positive control. Notably, **TPABTBP** did not induce any hemolysis within the tested concentration range (0–120 µm), as demonstrated by the absence of significant hemoglobin release (Figure S58, Supporting Information).

Moving on to in vivo evaluations, mice were administered **TPABTBP** (2 mg kg^−1^) via intravenous injection every 48 h for three consecutive doses. Control group mice received PBS solution injections under identical conditions. The safety profile was assessed by analyzing serum levels of liver and kidney function markers, including alanine aminotransferase (ALT), aspartate transaminase (AST), creatinine (CREA), and blood urea nitrogen (BUN). Importantly, there were no notable differences in hematological parameters or liver and kidney function between the **TPABTBP**‐injected group and the control group (Table S5 and Figure S59, Supporting Information). Furthermore, histological analysis of major tissues (kidney, spleen, heart, liver, and lungs) collected seven days after intravenous **TPABTBP** administration (2 mg kg^−1^) revealed no signs of necrosis, inflammation, hemorrhage, or other abnormalities. Hematoxylin and Eosin (H&E) staining confirmed the absence of tissue damage or pathological changes (Figure S60, Supporting Information).

These results demonstrated that **TPABTBP** exhibited negligible in vivo toxicity and excellent safety.

### 2.10 Imaging the Dynamic Changes of LDs in I/R Injured Mice

**Figure 7:**
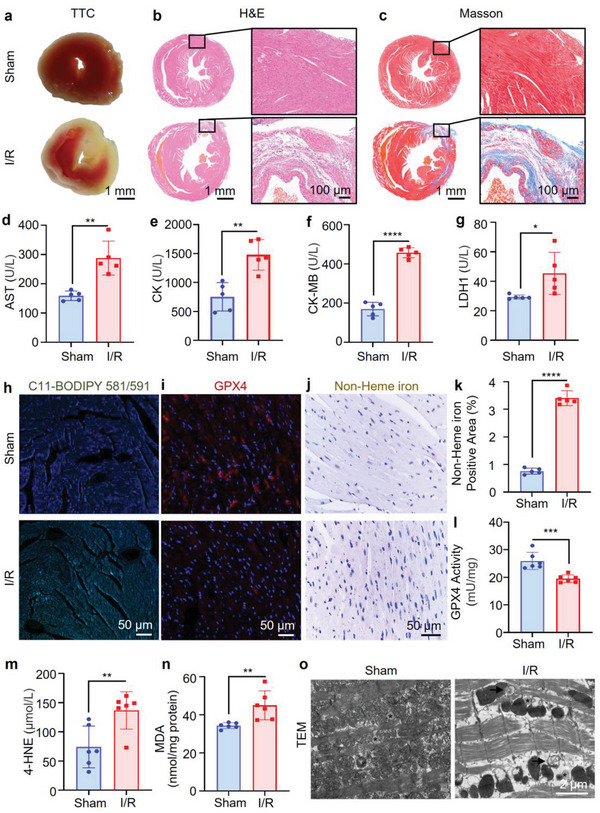
Evaluation of the correlation between ferroptosis and MIRI. a) TTC staining of heart slices. b, c) Scanned and magnified images of b) H&E stained and c) Masson's trichrome stained heart slices obtained from mice subjected to sham surgery or 30 min/4 weeks of I/R induction. Scale: 1000 µm (left), 100 µm (right). d‐g) Measurement of d) AST, e) CK, f) CK‐MB, and g) LDH1 levels in mice; *n =* 6. h, i) CLSM images showing sections of heart tissue labeled with h) C11‐BODIPY 581/591 and i) GPX4. Scale: 50 µm. j, k) Representative images and quantification of the nonheme iron‐positive area in heart sections stained with Prussian blue (enhanced with DAB) (*n =* 5). Scale bar: 50 µm. l‐n) Serum l) GPX4 levels, m) 4‐HNE levels, and n) MDA levels in mice; *n =* 6. o) The mouse myocardium, particularly the ultrastructure of the anterior wall of the left ventricle, was observed by TEM. (Black arrows: autophagic vacuole; *: LD.) Scale: 2 µm. Most of these groups, not groups b or c, underwent 30 min/24 h I/R and sham surgery. The statistical significance of the differences in the results presented in d‐g and k‐n was assessed by Student's t‐test; *p < 0.05; **p < 0.01; ***p < 0.001; ****p < 0.0001. Different letters indicate significant differences between labeled groups (*p <* 0.05).

In vitro experiments have confirmed that cardiomyocyte I/R injury can induce ferroptosis, a process accompanied by dynamic alterations in LD numbers. To delve deeper into the regulation of LDs on myocardial cell ferroptosis during I/R injury, we established a mouse model of MIRI.^[^ ^6^, ^44^ ^]^ The results of the triphenyltetrazolium chloride (TTC) assay revealed distinct myocardial ischemic areas following 30 min of ischemia and 24 h of reperfusion in mice subjected to MIRI compared to the sham‐operated group (**Figure** **7**a). Histological examinations, including hematoxylin and eosin (H&E) and Masson's staining, as well as myocardial enzyme analysis, further confirmed the successful induction of MIRI. The myocardial tissue from the I/R group exhibited irregular shape, disordered arrangement, abnormal intercellular spacing, and inflammatory cell infiltration, along with myocardial fibrosis indicated by blue‐colored collagen‐enriched areas in Masson's trichrome staining (Figure 7b,c). Moreover, biochemical markers such as aspartate transaminase (AST), creatine kinase (CK), creatine kinase‐MB isoenzyme (CK‐MB), and lactate dehydrogenase isoenzyme 1 (LDH1) were significantly elevated in the serum of mice subjected to MIRI compared to the sham group, confirming successful model establishment (Figure 7d–g).

Subsequently, we evaluated lipid peroxidation levels in myocardial tissue to investigate ferroptosis induction during MIRI. As shown in Figure 7h, MIRI led to a significant increase in lipid peroxidation levels compared to the sham group. Immunofluorescence assays revealed a notable decrease in glutathione peroxidase 4 (GPX4) protein expression after 24 h of reperfusion, further indicating ferroptosis induction (Figure 7i). Prussian blue staining with diaminobenzidine (DAB) revealed a marked accumulation of nonheme iron in myocardial tissues after 24 h of reperfusion, with a 4.5‐fold increase compared to the sham group (Figure 7j,k). To further validate ferroptosis induction, we measured the levels of ferroptosis‐associated markers (GPX4, 4‐HNE, and MDA) in blood serum.^[^ ^45^ ^]^ As shown in Figure 7l, after 24 h of myocardial reperfusion, the level of GPX4 in the serum was significantly lower than that in the sham group (19.17 vs 25.93 mU mg^−1^). Compared with those in the sham surgery group, the levels of 4‐HNE and MDA in the I/R group increased 1.8‐ and 1.3‐fold, respectively (Figure 7m,n). TEM imaging of myocardial tissue showed that the mitochondrial membrane densities were condensed in I/R injury mice, a typical feature of ferroptosis^[^ ^46^ ^]^ (Figure 7o). These results strongly confirm that myocardial I/R in mice damages the myocardium by inducing ferroptosis, which is consistent with the results of previous studies.^[^ ^6^, ^47^ ^]^

Next, we used the probe **TPABTBP** to image cardiomyocyte LDs during MIRI. Initially, we conducted in vivo biodistribution studies of **TPABTBP** to determine suitable imaging time points. Subsequently, fluorescence imaging and quantitative analysis on the dissected hearts of mice injected with **TPABTBP** (2 mg kg^−1^). The results, as depicted in Figure S61a,b (Supporting Information), revealed that cardiac fluorescence intensity at 6 h post‐injection was 2.5 and 1.7 times higher than at 1 and 3 h, respectively, with no significant difference observed between 6 and 12 or 24 h. These findings suggested that a 6 h interval was optimal for acquiring high‐quality cardiac imaging results. Furthermore, quantification of fluorescence signals in major organs (heart, lungs, liver, spleen, kidneys) indicated a robust signal of **TPABTBP** in the liver and kidneys (Figure S61c, Supporting Information). This observation aligns with the metabolic pathway of small molecules, primarily metabolized in the liver and excreted through the kidneys following intravenous injection.^[^ ^48^ ^]^

**Figure 8:**
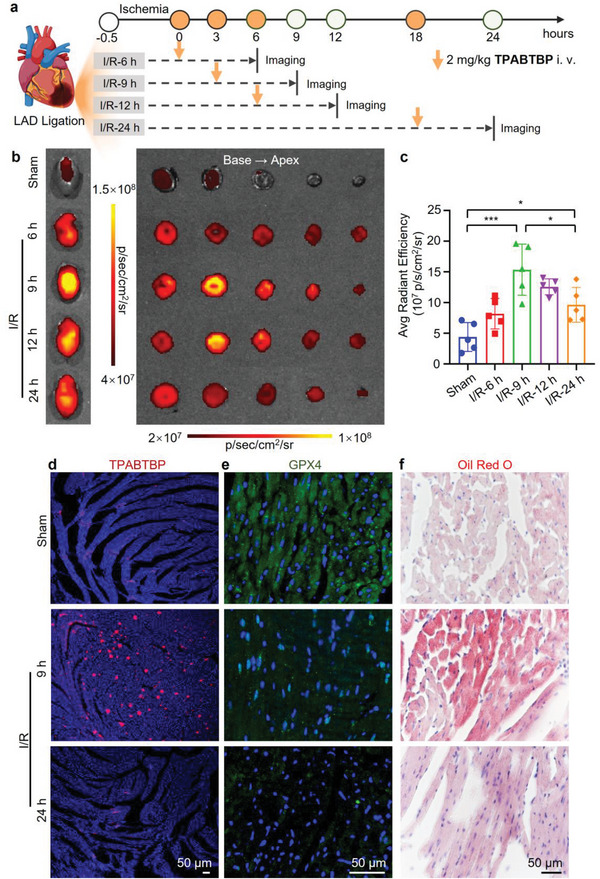
Monitoring the changes of LDs and GPX4 in MIRI mice. a) Schematic illustration of the imaging of the I/R injury hearts at different times. b) Representative ex vivo fluorescence image of hearts and sections in different groups (Sham, I/R‐6, I/R‐9, I/R‐12, and I/R‐24 h). c) Statistical analysis showing the average radiation efficiency in isolated hearts in the Sham, 6, 9, 12, and 24 h reperfusion groups; *n =* 5. d–f) Observation of mouse heart sections by different methods. The images are from the sham, 9, and 24 h reperfusion groups. d) CLSM images were used to measure the LD content in heart sections. Scale: 50 µm. e) CLSM images were used to measure the activity of GPX4 in heart sections. Scale: 50 µm. f) Oil red O staining revealed the LD distribution in heart sections. Scale: 50 µm. The statistical significance of differences in c was determined by Student's t‐test; *p < 0.05; **p < 0.01; ***p < 0.001. Different letters indicate significant differences between the labeled groups (*p <* 0.05).

We then utilized **TPABTBP** to track the LD content in myocardial tissue at different perfusion time points. In the experiment (**Figure** **8**a), mice underwent myocardial I/R and received intravenous injections of the **TPABTBP** probe (2 mg kg^−1^) after 0, 3, 6, and 18 h of reperfusion. After 6 h, their hearts were imaged *ex vivo*. To mitigate background fluorescence from the heart, mice in the sham operation group were also intravenously injected with the same amount of **TPABTBP** as controls. The imaging results revealed weak fluorescence in the hearts of sham‐operated mice. However, compared to the sham group, fluorescence intensity increased by 1.9‐fold after 6 h of reperfusion, further rising to 3.5‐fold after 9 h. Yet, this intensity decreased by 18.26% after 12 h compared to that at 9 h, followed by a 37.4% reduction after 24 h (Figure 8b,c). These results firmly confirm a significant increase in the number of LDs during the early stage (9 h) of myocardial I/R, with substantial decomposition occurring in the later stage of reperfusion. This observation aligns with previous in vitro cell experiments.

To verify that the specificity of the fluorescence signal in the heart was due to the binding of the probe **TPABTBP** to LDs, we froze and sliced the heart after imaging and observed it using confocal microscopy. As shown in Figure 8d, the LDs in the 24 h group were markedly reduced compared to those in the 9 h group. The GPX4 level in the 9 h group was higher than that in the 24 h group, suggesting that an increase in the number of LDs may exert an antagonistic effect on ferroptosis (Figure 8e). Oil red O staining of myocardial tissue confirmed that the number of LDs in the myocardial tissue increased significantly after 9 h of ischemia treatment but decreased after 24 h (Figure 8f). These findings suggest a strong correlation between MIRI and ferroptosis, with LD degradation promoting ferroptosis. During the early reperfusion stage, LD numbers in myocardial tissue notably rise, serving as reservoirs for FFAs, thereby inhibiting lipid peroxidation. However, during the later reperfusion stage, ferroptosis becomes inevitable, and LDs undergo extensive decomposition, releasing FFAs, which promote lipid peroxidation and exacerbate myocardial injury. Therefore, inhibiting LD decomposition during the early reperfusion stage may attenuate MIRI.

### 2.11 Chloroquine (CQ) Inhibits LD Degradation to Mitigate MIRI

**Figure 9:**
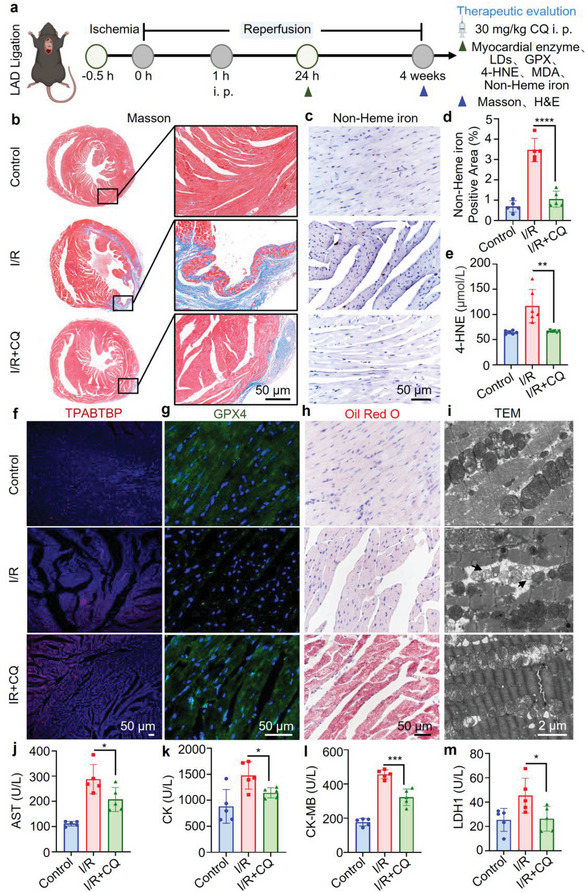
Validation of the therapeutic function of CQ. a) Schematic showing the evaluation of mice with MIRI after treatment with CQ. b) Scanned and magnified images of Masson's trichrome staining of heart slices taken from control mice or mice subjected to 30 min/4 weeks I/R injury and injected with saline or CQ. c) Analyses of heart sections stained with Prussian blue (enhanced with DAB) from the control (untreated), I/R, and CQ treatment group mice. d) Measurement and comparison of the nonheme‐iron‐positive area in control, I/R and CQ treatment group mice; *n =* 5. e) Measurement of serum 4‐HNE levels in control, I/R, and CQ treatment group mice; *n =* 6. f–i) Observation of mouse heart section images obtained via different methods. The images are from control, I/R, and CQ treatment group mice. f) Confocal images were used to observe LDs in heart sections labeled with **TPABTBP**. Scale: 50 µm. g) Fluorescence images were used to observe the activity of GPX4 in heart sections. Scale: 50 µm. h) Corresponding images of heart slices stained with Oil red O. Scale: 50 µm. i) The myocardium was observed by TEM to determine the ultrastructure of the anterior wall in the left ventricle. (Black arrows: autophagic vacuole.) Scale: 2 µm. j–m) Measurements of serum j) AST levels, k) CK levels, l) CK‐MB levels, and m) LDH1 levels in control, I/R, and CQ treatment group mice; *n =* 6. The statistical significance of differences shown in j‐m was determined by Student's t‐test; *p < 0.05; **p < 0.01; ***p < 0.001. Different letters indicate significant differences between the labeled groups (*p <* 0.05).

As previously demonstrated, CQ effectively inhibited LD decomposition in H9c2 cells, thereby mitigating ferroptosis. Subsequently, we sought to determine whether CQ could attenuate MIRI in mice. For early intervention, mice received intraperitoneal injections of CQ (30 mg kg^−1^) 1 h after reperfusion^[^ ^8^, ^49^ ^]^ (**Figure** **9**a). Serum and myocardial tissue were collected 24 h and 4 weeks post‐reperfusion to evaluate the therapeutic efficacy. In comparison to the I/R group, the CQ‐treated group exhibited a significant reduction in myocardial infarct size, as evidenced by TTC staining (Figure S62, Supporting Information). Additionally, myocardial fibrosis was notably diminished after CQ treatment (Figure 9b). Interestingly, nonheme iron levels were reduced by 70% in myocardial tissue after CQ treatment compared with that in the untreated group (Figure 9c,d). Similarly, after CQ treatment, serum 4‐HNE levels in mice were reduced by 43% compared with that in untreated groups (Figure 9e). These findings suggest that CQ effectively inhibits ferroptosis induced by myocardial I/R. We then investigated whether this protective effect was conferred to LD degradation inhibition. The probe **TPABTBP** and Oil red O were used to stain LDs in cardiac tissue after I/R. As shown in Figure 9f, the number of LDs in the myocardial tissue of mice after 24 h of I/R was significantly decreased, while the number of LDs was significantly increased after CQ treatment. Moreover, the extensive co‐localization of the red fluorescence signal from **TPABTBP** with that of BODIPY 493/503 confirmed the suitability of **TPABTBP** for specific LD imaging in myocardial tissue (Figure S63, Supporting Information). Oil red O staining of myocardial tissue indicated the increase in LD number in myocardial tissue after CQ treatment (Figure 9h). Immunofluorescence staining of GPX4 showed significantly higher GPX4 expression in the myocardium after CQ treatment than that in the untreated group (Figure 9g). The increased expression of GPX4 indicated that CQ treatment inhibited LD decomposition, thereby improving the ability of cardiomyocytes to resist lipid peroxidation and attenuate ferroptosis‐induced myocardial injury. To observe the ultrastructural changes in myocardial tissue after treatment, we performed TEM to examine the hearts of CQ‐treated mice. Our analysis revealed that I/R‐treated mice exhibited severe mitochondrial distortion and vacuolation, with numerous autophagic vesicles appearing in the cytoplasm. Then, CQ treatment mitigated the effects of I/R induction (Figure 9i). Moreover, the levels of myocardial enzymes (AST, CK, CK‐MB, and LDH) in serum, which increased due to I/R, were significantly reduced in mice treated with CQ (Figure 9j–m). Taken together, these results suggest that CQ exerts a therapeutic effect on MIRI by inhibiting ferroptosis. We hypothesize that CQ may reduce LD degradation and mitigate ferroptosis by inhibiting lipophagy,^[^ ^41a^ ^]^ thereby alleviating MIRI injury. However, the mechanism underlying this effect remains to be further elucidated.

Subsequently, we examined the biosafety of CQ in vivo. The serum and major organs of the mice were collected for evaluation 24 h after intraperitoneal injection of 30 mg kg^−1^) CQ. As shown in Figure S64 (Supporting Information), levels of the myocardial enzymes (AST, CK, CK‐MB, and LDH1) in the CQ‐treated group did not significantly differ from those of the control, confirming the absence of significant cardiotoxicity induced by **TPABTBP**. Furthermore, serum levels of ALT, BUN, and CREA were within normal ranges, suggesting unaffected liver and kidney metabolism in the mice (Figure S65, Supporting Information). Additionally, H&E staining of major organs revealed structurally intact cardiomyocytes with well‐defined and regular nuclei. No significant pathological damage was observed in the livers, spleens, or kidneys (Figure S60, Supporting Information). Taken together, these results demonstrate that CQ shows good biosafety and is a prospective medication for treating MIRI.

## 3 Conclusion

In summary, we developed an LD‐activated AIE probe, **TPABTBP**, for monitoring the dynamic changes in LD number during myocardial I/R‐induced ferroptosis in mice. The probe **TPABTBP** showed excellent LD‐targeted imaging ability and good biocompatibility. MD simulations and super‐resolution FI confirmed that the probe **TPABTBP** was specifically localized to the phospholipid monolayer membrane of LDs. Compared to the commercially available LD fluorescence dye BODIPY 493/503, **TPABTBP** demonstrated superior LD specificity, enhanced capability for monitoring lipophagy, and remarkable photostability. Using **TPABTBP** as an imaging probe revealed that LD accumulation in cardiomyocytes was increased during the early stage of ferroptosis but decreased via lipophagy in the late stage. Inhibition of autophagy or knockdown of the lipophagy‐related gene RAB7A led to a reduction in the decomposition of LDs and conferred protection against ferroptosis damage onto cardiomyocytes. Imaging of myocardial I/R injured mice with **TPABTBP** showed that the LD content in myocardial tissue increased significantly after 9 h of reperfusion and decreased after 24 h. More importantly, we confirmed that CQ inhibited LD decomposition to alleviate MIR‐induced ferroptosis in vivo. However, whether the inhibitory effect of CQ on ferroptosis is mediated through mechanisms associated with lipophagy requires further investigation.

## 4 Experimental Section

### Materials

BODIPY 493/503 (D3922), ProLong Gold antifade reagent (P10144), C11‐BODIPY 581/591 (D3861), Mito‐Tracker Green FM (M7514), ER‐Tracker Green (E34251), NBD C6‐Ceramide‐BSA complex (N1154), and LysoTracker Red DND‐99 (L7528) and Lipofectamine 3000 were purchased from Thermo Fisher Scientific (USA). Mild immunoprecipitation (IP) cell lysis solution (P0013), glutathione peroxidase 4 (GPX4, S0058), 4′,6‐diamidino‐2‐phenylindole (DAPI, C1006), Calcein‐AM/PI assay kit, QuickBlock immunostaining blocking solution (P0260) and Alexa Fluor 488‐labeled Goat Anti‐Rabbit IgG (H+L) (A0423) were obtained from Beyotime Biotechnology (Nanjing, China). Erastin (HY‐15763), rapamycin (RAPA, HY‐10219), chloroquine (CQ, HY‐17589A), and 3‐methyladenine (MA) (HY‐19312) were acquired from MedChemExpress (Monmouth Junction, NJ, USA). Oil red O, hematoxylin, Prussian blue iron stain (enhanced with DAB) kits, and BCA protein assay kit (G2026) were procured from Servicebio Technology Co., Ltd. (China). A malondialdehyde (MDA) assay kit and 2,3,5‐triphenyl‐2H‐tetrazolium chloride (TTC) assay kit were purchased from Solarbio Science & Technology Co., Ltd. (Beijing, China). A 4‐hydroxynonenal (4‐HNE) ELISA kit was obtained from Mlbio Biotechnology Co., Ltd. (China). Oleic acid (OA, O1008) was bought from Sigma‐Aldrich (St. Louis., MO, USA). Blood/cell/tissue genomic DNA extraction kit (DP304) was obtained from TIANGEN Biochemical Technology Co., Ltd (China). The lipid droplet isolation kit (MET‐5011) was procured from CellBiolabs Inc (USA). A mCherry‐eGFP‐LC3 lentivirus was acquired from Hanbio Biotechnology Co., Ltd. (China). Antibodies against RAB7A (R220246) and Perilipin‐2 (PLIN2, ab108323) were obtained from Abcam Trading Co., Ltd. (UK). The GFP‐tagged PLIN2 plasmid was a gift from Ding Binbin's group (Huazhong University of Science and Technology). Analytical‐grade solvents and reagents were utilized in all experiments.

### Measurement of Fluorescence Quantum Yields

The fluorescence quantum yields of **TPABTBP**, **BTDPP**, and **PMBTDP** in various solvents were measured using an Edinburgh Instruments FS5 steady‐state and time‐resolved fluorescence spectrometer, which was conducted under the following conditions: excitation at 480 nm, emission collection ranged from 500 to 800 nm at intervals of 1 nm, with a signal dwell time set at 0.2 s.

### Cell Culture

The RAW264.7, HeLa, HepG2, and H9c2 cell lines (Chinese Academy of Sciences Cell Bank, China) were cultured using Dulbecco's modified Eagle medium (DMEM, Gibco) containing 1% penicillin‒streptomycin (Gibco) and 10% fetal bovine serum (Gibco, Australia, NY). The cells were incubated at 37 °C with 5% CO_2_ (Thermo Fisher Scientific, USA).

### Dynamic Simulation Method

All molecular dynamics (MD) were performed with the massively parallel simulation package GROMACS version 2020.6.^[^ ^50^ ^]^ The phospholipids, triacylglycerols (TAGs), and ions were modeled with CHARM36 force field. The TIP3P water model^[^ ^51^ ^]^ and compatible force filed parameters for **TPABTBP** were determined with CgenFF.^[^ ^52^ ^]^ Long‐range electrostatics were performed using the particle mesh Ewald (PME) method.^[^ ^53^ ^]^ Van der Waals interactions were truncated at a specified cutoff distance of 1.2 nm.

First, solvated phospholipid bilayer was created, which was ≈2 nm thick and consisted of 1‐palmitoyl‐2‐linoleoyl‐sn‐glycero‐3‐phosphatidylcholine and 1,2‐dipalmitoleoyl‐sn‐glycero‐3‐phosphoethanolamine (77:51). The bilayer was immersed in a 0.15 m KCl solution, and the construction was performed using the CHARMM‐GUI membrane builder.^[^ ^54^ ^]^ Subsequently, it underwent equilibration in the isothermal‐isobaric (NPT) ensemble for 100 ns. The NPT parameters were as follows:

The energy minimization was carried out using a combined steepest descent approach with termination gradients set at 100 kJ mol^−1^.nm. Temperature and pressure control were maintained at 300 K and 1 bar using the Nose´‐Hoover thermostat^[^ ^55^ ^]^ and the Berendsen barostat.^[^ ^55^ ^]^ Periodic boundary conditions were applied in all three dimensions. Long‐range electrostatics were computed with the Particle Mesh‐Ewald method,^[^ ^53^ ^]^ employing a relative tolerance of 1 × 10^−6^. Both real‐space Ewald interactions and van der Waals interactions had a cutoff distance of 1.2 nm. To constrain bond lengths of hydrogen atoms, the LINCS algorithm^[^ ^56^ ^]^ was employed, while a leapfrog algorithm^[^ ^57^ ^]^ with a time step of 2 fs was employed.

We used Packmol software to build TAG Slab. Prior to assembling the complete simulation system, a separate 20 ns NPT simulation was conducted on the TAG slab (10 nm‐thick) under conditions of 300 K and 1 bar to attain an equilibrium state. The simulation parameters for NPT were the same as mentioned above.

Subsequently, a collaboration between the Visual Molecular Dynamics (VMD) and the Packmol software was utilized to insert the equilibrated TAG slab between the upper and lower phospholipid monolayers. This combined system consisted of 256 phospholipids, 555 TAGs, 14 365 water molecules, and 54 ion pairs. After 10 ns of equilibration within the NPT ensemble at 300 K and 1 atm, the resulting dimensions of the system were 8.7 × 8.7 × 18.7 nm. Temperature was regulated using a Nose‐Hoover thermostat, and pressure was maintained using a Berendsen barostat.

In the unrestrained MD setup, a single **TPABTBP** molecule was initially positioned, situated ≈ 1–1.5 nm above the phospholipid membrane within the equilibrated slab system. Subsequently, a continuous MD simulation trajectory of 500 ns was generated for the **TPABTBP** system using a canonical (NVT) ensemble at 300 K. The specific NVT parameters were as follows:

We initiated the energy minimization process using a composite steepest descent protocol with termination gradients set at 100 kJ mol^−1^ nm. Temperature control at 300 K was maintained through the Nose´–Hoover thermostat.^[^ ^55^ ^]^ Periodic boundary conditions were applied in all three dimensions. For long‐range electrostatic interactions, the Particle Mesh‐Ewald method^[^ ^53^ ^]^ was employed, with a relative tolerance of 1×10^−6^. We utilized a 1.2 nm cutoff distance for real‐space Ewald interactions and applied the same value for van der Waals interactions. Hydrogen atom bond lengths were constrained using the LINCS algorithm,^[^ ^56^ ^]^ and a leapfrog algorithm^[^ ^57^ ^]^ was used with a time step of 2 fs.

The migration depth was defined as the vertical distance (along the *z*‐axis) between the center of mass of **TPABTBP** molecules and the average position of the headgroup plane in the phospholipid bilayer. The headgroup plane was determined by calculating the average position of phosphorus atoms along the *z*‐axis.

To analyze the free energy landscape of **TPABTBP** migration through the two interfaces, a multi‐walker well‐tempered meta‐dynamics simulation was conducted with 4 replica walkers. For **TPABTBP**, four initial representative structures were selected from unrestrained MD simulation trajectories, each with varying migration depths ranging from −2 to −0.5 (−2 ≤ *z* ≤ −0.5). In each of these four simulations, **TPABTBP** was initially placed within the phospholipid layer. We employed two collective variables (CV) to characterize the migration depth (*z*) of **TPABTBP** and the angle. The angle was defined as the angle between the *R*_end to end_ vector and the membrane normal. The parameters of meta‐dynamics were set as follows:

Gaussians were deposited at intervals of 500‐time steps with an initial height of 1.2 kJ mol^−1^. A bias factor of 5 was judiciously chosen. The Gaussian width (sigma) was determined based on the fluctuation of the collective variables observed during the unbiased run and set at 0.2. The system was maintained at a temperature of 300 K. And other parameters were defaults. The total time of meta‐dynamics simulation was 5 ns for each initial representative structure.

### Isolation and Visualization of Lipid Droplets (LDs) in HepG2 Cells

HepG2 cells (3 × 10^7^) were initially treated with trypsin and subsequently resuspended in a complete growth medium. The cell suspension was then subjected to centrifugation at 1000 g for 5 min. After centrifugation, the culture medium was carefully removed. These cells were washed with 10 mL PBS, and centrifuged again at 1000 g for 5 min. Subsequently, the cell pellet was resuspended in 1 mL of PBS. The resuspended cells were transferred into a 2 mL Eppendorf (EP) tube, and centrifuged again at 1000 g for 5 min. The supernatant was then removed. Next, the pellet was thoroughly resuspended in 200 µL of Reagent A and incubated on ice for 10 min. Carefully, 800 µL of 1×Reagent B was added to the mixture and thoroughly mixed. After incubating on ice for 10 min, the cells were homogenized by passing them through a 27‐gauge needle attached to a 3 mL syringe five times. Subsequently, briefly centrifuge the homogenate at 100 g for 5 s. Finally, 600 µL of 1× Reagent B was layered on top of the homogenate. The EP tube containing the layered components was subjected to centrifugation in a microcentrifuge for 3 h at a force ranging from 18 000 to 20 000 g at a temperature of 4 °C. After centrifugation, 270 µL of floating LDs from the top of the EP tube were collected. These LDs were subsequently transferred for storage at −80 °C.

Subsequently, disperse 10 µL LDs in 0.5 mL cyclohexane and subject the mixture to ultrasonic dispersion for a duration of 10 min. Following this step, incubate the resulting LD solution with 1 mM **TPABTBP** at room temperature for a period of 3 min. Immediately, transfer 10 µL of the resulting mixture onto a glass slide and proceed to observe it under a multispectral fluorescence microscope.

Moreover, HepG2 cells were seeded in confocal dishes, followed by staining with 30 µM **TPABTBP** for 0.5 h. Next, the cells were washed with PBS, fixed with 4% paraformaldehyde for 10 min, and then labeled with a DAPI staining solution for nuclear identification. Finally, LDs within the HepG2 cell cytoplasm were observed using a 100× oil immersion lens on a multi‐color fluorescence laser confocal microscope (FV3000, Olympus, Japan).

### Co‐localization Analysis of GFP‐tagged PLIN2 and **TPABTBP**

Initially, 5 × 10^4^ HepG2 cells were seeded into confocal dishes and cultured overnight at 37 °C with 5% CO_2_. Subsequently, GFP‐tagged PLIN2 was diluted in Opti‐MEM medium, and HepG2 cells overexpressing GFP‐tagged PLIN2 were constructed via transfection according to the manufacturer's instructions using Lipo3000. Following this, HepG2 cells were co‐cultured with 30 µM **TPABTBP** for 0.5 h. The HepG2 cells were then washed with PBS, fixed with 4% paraformaldehyde, and finally washed three times with PBS. The LDs within the HepG2 cells were observed using a laser confocal microscope (FV3000, Olympus, Japan).

### In Situ Time‐Lapse Confocal Imaging of LDs

HepG2 cells (1 × 10^4^) were seeded into a confocal dish and cultured at 37 °C with 5% CO_2_ for 12 h. Subsequently, the cells were incubated with 30 µM **TPABTBP** staining solution without serum for 0.5 h. Following incubation, the cells were rinsed with PBS containing 1% FBS. The confocal dish was then placed in a live‐cell workstation. The observation of LDs was conducted using a comprehensive spectral ultra‐high resolution laser confocal scanning system, which performed a layered scan across five Z‐positions on HepG2 cells (Stellaris STED, Leica, Germany).

### Structured Illumination Microscopy (SIM) Imaging of LDs

H9c2 and HepG2 cells (1 × 10^4^ per slide) were seeded into glass cover slides and cultured for 12 h at 37 °C with 5% CO_2_. LDs in H9c2 cells were labeled by incubating them with **TPABTBP** (30 µM) and BODIPY 493/503 (5 µM) in serum‐free medium at 37°C in the dark, and HepG2 cells were also labeled by incubating them with **TPABTBP** (30 µM). After 0.5 h of incubation, the treated cells were washed 3 times with PBS and preserved in ProLong Gold antifade reagent. Finally, the LDs were observed under a Lattice SIM^2^ ZEISS Elyra 7 microscope.^[^ ^58^ ^]^

### Responsiveness of Different Probes to Cellular LDs

HeLa, RAW264.7, and H9c2 cells were seeded at 1 × 10^4^ cells mL^−1^ into confocal dishes. After 12 h in culture, the medium was removed, and the cells were co‐incubated with **PMBTDP** (10 µM), **BTDPP** (10 µM), **TPABTBP** (10, 30 µM) and BODIPY 493/503 (5 µM) for 0.5 h. The cells were then washed three times with PBS in the confocal culture dish and fixed using a 4% paraformaldehyde solution. DAPI staining was performed for 10 min. Then, images were captured using a Zeiss LSM 780 single‐photon laser confocal scanning microscope, and fluorescence colocalization was analyzed using ImageJ software.

### Immunofluorescence Staining

H9c2 cells were initially seeded onto glass coverslips and incubated overnight at 37 °C under 5% CO_2_ conditions. Subsequently, the cells were treated with 30 µM **TPABTBP** for 30 min. Afterward, the cells were washed three times with PBS and fixed in 4% paraformaldehyde at room temperature for 10 min. Following fixation, the cells were permeabilized with PBS containing 0.1% Triton X‐100 for 5 min. H9c2 cells were then washed three times with PBS for 5 min each. Subsequently, the cells were treated with QuickBlock immunostaining blocking solution for 10 min. For overnight incubation at 4 °C, cells were treated with Rabbit monoclonal antibody to PLIN2, following the manufacturer's instructions. Alexa Fluor 488‐labeled Goat Anti‐Rabbit IgG (H+L) was then added to the cells for visualization. DAPI was used for nuclear counterstaining. Finally, images were obtained using a confocal microscope.

### Analysis of Colocalization between LDs and Cellular Organelles

At first, BODIPY 493/503, Mito‐Tracker Green FM, ER‐TrackerGreen, NBD C6‐Ceramide, and LysoTracker Red DND‐99 were dissolved and prepared as 1 mM stock solutions according to the manufacturer's instructions. Notably, the NBD C6‐Ceramide required further reaction with BSA to generate NBD C6‐Ceramide‐BSA complex. Subsequently, these organelle stock solutions were diluted in serum‐free DMEM culture medium to achieve working concentrations of, 5 µM BODIPY 493/503 for LDs labeling, 100 nM Mito Tracker Green FM for mitochondrial (Mito) labeling, 1 µM ER‐TrackerGreen for endoplasmic reticulum (ER) labeling, 5 µM NBD C6‐Ceramide‐BSA complex for Golgi apparatus (GA) labeling, and 75 nM LysoTracker Red DND‐99 for lysosome (Lyso) labeling.

HepG2 cells (5 × 10^4^ cells mL^−1^) were seeded into 35 mm glass‐bottom culture dishes and incubated for 12 h at 37 °C with 5% CO_2_. After the incubation period, cells were treated with 1 mL LDs, Mito, ER, GA, or Lyso tracker work solution for 30 min. Following probe incubation, cells were washed three times with HBSS buffer. Subsequently, HepG2 cells were treated with 30 µM **TPABTBP** for 30 min. Finally, cells were subjected to confocal microscopy analysis. Image analysis was performed using ImageJ.

Furthermore, H9c2 cells (5 × 10^4^ cells mL^−1^) were seeded into 35 mm glass‐bottom culture dishes and incubated overnight at 37 °C with 5% CO_2_. Subsequently, the cells were treated with 1 mL of 75 nM LysoTracker Red DND‐99 for 30 min. Afterward, the cells were washed three times with HBSS buffer, followed by treatment with 30 µM **TPABTBP** or 5 µM BODIPY493/503 for 30 min. Finally, the cells were stained with DAPI for 10 min and subjected to confocal microscopy analysis.

### Detection of Cellular LDs after Ferroptosis Induction

Hela, H9c2, and HepG2 cells (1 × 10^4^ cells per well) were treated with 5 µM Erastin in a growth medium for 1, 3, 6, 9, 12, 16, 20, and 24 h. These cells were incubated with **TPABTBP** (30 µM) in confocal dishes or 6‐well plates at 37 °C for 0.5 h. Subsequently, the cells in the 6‐well plate were washed three times with PBS before being detached from the dish by scraping. Next, these cells were washed with PBS, fixed with paraformaldehyde, and stained with DAPI. Confocal fluorescence scanning microscopy (CLSM) was then performed to obtain fluorescence images. The intensity of LD staining was quantified by measuring the mean fluorescence intensity (MFI) within cells. MFI was quantified in individual cells using ImageJ (Fiji 2.15.0), and *n =* 3 for each measurement.

Besides, H9c2 cells were further incubated with **TPABTBP** (30 µM) in 6‐well plates at 37 °C for 0.5 h. Subsequently, the cells were washed three times with PBS before being detached from the well by scraping. H9c2 cells were resuspended in 300 µL of PBS and used for flow cytometry measurements (Alex488 channel, SONY ID7000 full spectrum analysis flow cytometer).

In addition, H9c2 cells were stained with Oil Red O and the intracellular distribution of the LDs after Erastin treatment was observed at different time points (3, 6, 9, 12, and 24 h). First, the post‐induced H9c2 cells were fixed with 4% paraformaldehyde. Subsequently, they were covered with 60% isopropanol solution and allowed to incubate for 15 s. Following this, the isopropanol solution was removed, Oil Red O was introduced, and the cells were immersed in the dye for a period of 0.5 h. Next, the cell nuclei were stained with hematoxylin for 3 min. The cells were counterstained with aniline blue and then washed with water. Finally, the LDs were photographed with a Nikon Ni‐E optical microscope after a drop of glycerol‐gelatin was applied to seal the sample.

### Lipid Peroxidation Assay

H9c2 cells were seeded in a 6‐well plate or confocal dish (1 × 10^4^ cells mL^−1^). C11‐BODIPY 581/591 was dissolved in dimethyl sulfoxide (DMSO) to produce a 5 µM working solution. In the reduced state, the excitation and emission maxima of C11 BODIPY 581/591 was 581/591 nm; after oxidation, the probe shifted the excitation and emission to 488/510 nm. The cells were then collected and incubated with this C11‐BOPIPY 581/591 working solution for a total of 0.5 h after treatment with Erastin for 3, 6, 9, 12, or 24 h. For cell counting by flow cytometry (in the BODIPY channel, *E_ex_* = 488 nm), the H9c2 cells in the 6‐well plate were removed and suspended in 0.3 mL of PBS. After fixing and staining with DAPI, the cells were analyzed by CLSM.

### Hypoxia/Reoxygenation (H/R) Cell Culture

To simulate MIRI in vitro, a hypoxic culture system was constructed according to the AnaeroPack (Mitsubishi, Japan) instructions. In brief, culture bags, disposable anaerobic oxygen capsules, oxygen indicators, and airtight containers were used. H9c2 cells were cultured overnight at 37 °C with 5% CO_2_ until the cells adhered to the walls of culture dishes with the oxygen concentration at 0.1% and the CO_2_ level at 5% for 1 h in the cell culture system. After 12 h of anoxic incubation, the dishes with H9c2 cells were removed from the closed vessel and placed in a reoxygenation system for culturing at 37 °C for 2 h. Finally, the experiment was terminated, and the cells were labeled with C11‐BODIPY 581/591 (5 µM) and **TPABTBP** (30 µM) for 0.5 h, fixed and washed. The cells were then observed by CLSM.

### Calcein‐AM /PI Staining

H9c2 cells were seeded in 96‐well plates or confocal culture dishes (1 × 10^4^ cells mL^−1^). Subsequently, a detection buffer was utilized to dilute the Calcein‐AM and propidium iodide (PI) staining solutions by a factor of 1000, creating staining working solutions. Following hypoxia‐reoxygenation cultivation or induction with Erastin for 3, 6, 9, 12, or 24 h, cells were collected and washed once with PBS. The combined Calcein‐AM and PI working solutions were then applied to the cells, followed by incubation in the dark for 0.5 h. After staining, detection was performed using laser confocal microscopy or a fluorescence microplate reader. (Calcein‐AM excitation/emissio*n =* 494/517 nm; PI excitation/emissio*n =* 535/617 nm).

### Transmission Electron Microscopy (TEM)

The cells and tissues were exposed to a solution containing 2.5% glutaraldehyde, 2% paraformaldehyde, and 2 mM CaCl_2_ in a 0.15 m sodium calcium carbonate buffer solution at room temperature for a duration of 2 h. Then, the specimens were fixed in a 1% osmic acid solution for another 2 h. The samples were washed three times with 0.1 M PBS, sliced, and dehydrated with acetone. Epoxy 812 (Ted Pella) was used to embed the specimens. After polymerization of the specimens in an oven at 60 °C for 48 h, ultrathin sections (70 nm) were prepared under an ultramicroscope (Leica EM UC7) with a diamond sectioning knife (Ultra 45°) and placed on copper mesh (300 mesh). Uranium‒lead double‐stained sections were imaged with a Hitachi HT7700 TEM system operating at 80 kV.

### Inhibition of Cellular Autophagy

H9c2 cells were cultured with 5 µM Erastin and 100 nM rapamycin (RAPA) or 10 mM 3‐methylladenine (3‐MA) for 20 h. The H9c2 cells were then labeled with 30 µM **TPABTBP** or 5 µM C11‐BODIPY 581/591. After 0.5 h of incubation, the cells were fixed with 4% paraformaldehyde and labeled with DAPI. Subsequently, the H9c2 cells were observed by fluorescence microscopy, and the percentage of positive cells was determined based on the flow cytometry data.

### Generation and Validation of Knockout ATG5 Cell Line

For the generation of ATG5 knockout (KO) cells, single guide RNAs (sgRNAs) were designed using the online CRISPR design tool (Red Cotton, China). Specifically, sgRNA‐1 (g5) had the sequence GTGATAGGTGTGCGGAAGTTGG, and sgRNA‐2 (g6) had the sequence AGAACTCTACTCCGTGGTTTAGG (Table S3, Supporting Information). These sgRNAs were designed to target the exon region of the ATG5 gene for CRISPR/Cas9 genome editing. A prioritized list of sgRNAs was generated, taking into account both specificity and efficiency scores. Oligos corresponding to the two targeting sites were annealed and ligated into the YKO‐RP006 vector (Ubigene Biosciences Co., Ltd., China). The resulting YKO‐RP006‐hRABL6[gRNA] plasmids containing the target sgRNA sequences were transfected into the target cells using Lipofectamine 3000 (Thermo Fisher Scientific). Subsequently, 24–48 h after transfection, puromycin was introduced to facilitate the selection of successfully edited cells. Following antibiotic selection, a specific number of cells were subjected to limited dilution and subsequently inoculated into a 96‐well plate. Single clones were selected after 2–4 weeks of culture.

In addition, these ATG5 KO clones were subsequently validated using PCR and Sanger sequencing. The genomic DNA from H9c2 cells was extracted using the genomic DNA extraction kit. Simultaneously, primers were designed to target the knockout region for PCR amplification. The nucleic acids were subjected to denaturation, annealing, and extension steps, followed by further analysis of the target gene's knockout status through gel electrophoresis. Furthermore, PCR products were subjected to TA cloning, followed by Sanger sequencing to definitively determine the mutation status of each allele. The sgRNAs and primer sequences employed for PCR design can be found in Table S4 (Supporting Information).

### Detection of Autophagy Flux

H9c2 cells and ATG5 KO cells were seeded in a 6‐well plate and allowed to achieve 50%−70% confluence before transfection. According to the manufacturer's instructions, cells were infected with mCherry‐eGFP‐LC3 adenovirus in the culture medium for 24 h. Subsequently, the culture medium was replaced with a fresh complete medium, and the cells were further incubated at 37 °C for an additional 24 h. Following this incubation period, the H9c2 cells were treated with a combination of RAPA (100 nM), 3‐MA (10 mM), or CQ (5 µM), in addition to 5 µM Erastin, and incubated further. Finally, images were acquired using laser confocal microscopy, and ImageJ was used to count yellow puncta (autophagosome) and red puncta (autolysosome).

### In Vitro RAB7A Knockdown

H9c2 cells were transfected with short interfering RNA (siRNA), specifically, RAB7A‐siRNA‐3 (5′‐AUCAAACACCAGAACACAGCA‐3′) and negative control siRNA (Ctrl, 5′ ACGUGACACGUUCGGAGAA‐3′), obtained from Kincaid Biologicals Co., Ltd. (Wuhan, China). RNA interference (RNAi) experiments were performed with H9c2 cells after Lipofectamine 3000 treatment according to the instructions of the manufacturer in combination with Opti‐MEM. The expression of the genes targeted with siRNA was further verified using quantitative PCR (qPCR) and Western blot (WB) analysis.

### qPCR

Total mRNA was extracted from H9c2 cells with RAB7A knockdown, as well as from cultured cells, using TRIzol reagent. Subsequently, cDNA synthesis was carried out employing reverse transcription reagents from Vazyme. qPCR was conducted using PerfectStart Green qPCR SuperMix (+Dye II), following the manufacturer's recommended protocols. The mRNA expression levels of the relevant genes were normalized to the expression of the housekeeping gene β‐actin.

### WB

Total protein was extracted and lysed from the RAB7A downregulation H9c2 cells using RIPA lysis buffer. Protein concentrations were measured using the BCA protein assay kit. Equal amounts of the specified proteins were loaded onto a 10% SDS–PAGE gel and subsequently transferred onto PVDF membranes. Afterward, these membranes were blocked with a 5% skim milk solution in TBST and incubated with their respective primary antibodies overnight at 4 °C, followed by incubation with HRP‐conjugated secondary antibodies for 1 h. Protein bands were visualized using an ECL kit and analyzed with an AIWBwell imaging system (Servicebio).

### Detection of LDs in RAB7A‐Knocked Down Cells

H9c2 cells with RAB7A knocked down were seeded (1 × 10^5^ cells mL^−1^) in a 6‐well plate or confocal dish and exposed to 5 µm Erastin in medium for 20 h or H/R cell culture. These cells were labeled with 30 µM **TPABTBP** at 37 °C for 0.5 h. Next, the cells in the confocal culture dish were washed with PBS and treated with 4% paraformaldehyde. DAPI was used to stain nuclei, and fluorescence images were obtained by CLSM.

### Evaluation of Lipid Peroxidation in RAB7A‐Knocked Down Cells

H9c2 cells were successfully transfected with the target RAB7A siRNA in a 12‐h incubation period and then subjected to Erastin treatment (5 µM, 20 h). The RAB7A downregulation H9c2 cells were labeled with 5 µM C11‐BODIPY 581/591 at 37 °C for 0.5 h. Subsequently, the cells in the confocal culture dish were washed with PBS and treated with 4% paraformaldehyde. Nuclei were stained with DAPI, and fluorescence images were captured using confocal laser scanning microscopy (CLSM).

Moreover, the RAB7A‐knocked down H9c2 cells were collected and exposed to IP solution for 30 s on ice. The cells were lysed by ultrasonic fragmentation (2 s of ultrasound treatment at 3 s intervals for 10 cycles). Finally, the mixture was subjected to high‐speed centrifugation (12 000 rpm, 10 min), and the upper layer was collected. The extracted proteins were assayed using the instructions provided with the GPX4 and MDA measurement kits. Quantitative data were obtained using a microplate reader (TECAN).

### CQ Intervention of Lipophagy

H9c2 cells (1 × 10^4^ cells mL^−1^) were inoculated into a 6‐well plate or uniformly inoculated onto glass coverslips and treated with a 5 µM CQ and 5 µM Erastin mixture for 20 h. H9c2 cells were then stained with **TPABTBP** (30 µM) or C11‐BODIPY 581/591 (5 µM). The cells were obtained for subsequent quantification by flow cytometry. H9c2 cells on glass coverslips were treated with 4% paraformaldehyde and then stained with DAPI for 10 min. The slides were sealed with an anti‐fluorescence quencher. Finally, fluorescence images were acquired by Zeiss LSM 800 microscopy.

### Animal Models

Adult male mice (C57BL/6J, Beijing Vital River Laboratory) that were 8–12 weeks old were used in this study. They were bred in a specific‐pathogen‐free (SPF) animal house in ventilated cages with standard corncob bedding. Mice could freely obtain pure water treated by reverse osmosis (RO) and radiation‐sterilized feed. All animal procedures were approved by the Institutional Animal Care and Use Committee (IACUC) of Tongji Medical College, Huazhong University of Science and Technology (IACUC number: 3125) in accordance with the National Institutes of Health (NIH) guidelines for the care and use of laboratory animals.

The mice were randomly assigned to three groups: sham group, ischemia/reperfusion (I/R) group, and CQ treatment group. Mice were anesthetized by intraperitoneal (i.p.) injection of pentobarbital (60 mg kg^−1^) and placed on a warming pad maintained at 37 °C to establish MIRI. After entering the anesthetization maintenance period, the mice were intubated with a 22‐gauge intravenous catheter, and mechanical ventilation was applied with an ALC‐V8‐SLA animal ventilator (Alcbio, China) at a rate of 110 breaths per minute. The third and fourth intercostal spaces were moved to expose the heart, and sterile 8‐0 absorbable sutures (Jinhuan, China) were used to close the left anterior descending (LAD) coronary artery, which was tied tightly with sterile thread. The left ventricle wall turned pale, indicating that the ligation had been properly positioned. After 30 min of ischemia, the 8‐0 suture was cut to induce LAD coronary artery reperfusion. Finally, the muscles and skin on the chest were sutured closed. The animals in the sham group underwent the same procedure as the experimental group, except for ligation of the LAD coronary artery. In the treatment group, CQ (30 mg kg^−1^) was injected i.p. ≈1 h after reperfusion and was allowed to circulate in the mice for 24 h. The other steps in the procedure were the same as those used for the I/R group.

### Histological Assessment of the Infarct Area

Following 24 h of reperfusion, the heart was removed and rinsed in PBS. Hearts were sliced transversely into 4–5 sections in parallel to the atrioventricular groove and frozen at −20 °C for 20 min. The powder in TTC kit was removed and dissolved in PBS to prepare a 1% TTC solution. Subsequently, the tissue slices were immersed in the TTC dye solution, incubated in a 37 °C water bath in the absence of light for 0.5 h, and then extracted. Finally, the cells were fixed with polyformaldehyde for a duration of 15 min.

After 4 weeks of reperfusion, fresh myocardial tissue was fixed in 4% paraformaldehyde. It was embedded in paraffin and sectioned into consecutive sections. These slices were stained using hematoxylin and eosin (H&E) for use in a conventional histological examination via microscopy. For Masson's staining, paraffin sections were precultured overnight in a drying oven at 37 °C. After dewaxing and rehydration, the sections were fixed in Bouin solution at 56 °C for 90 min. After washing with running water for 20 min, the sections were stained with hematoxylin solution for 15 min and Biebrich fuchsin solution for 20 min at 20 °C. The cells were counterstained with aniline blue for 15 min and incubated in 1% acetic acid for 2 min at 20 °C. Imaging of these sections were obtained with a slice‐scanning system (OLYMPUS, VS120).

### Myocardial Enzyme Levels

Twenty‐four hours after MIRI surgery, mouse blood was centrifuged at 3000 rpm for 15 min at 4 °C for zymography. The serum was isolated. Measurements of four myocardial enzymes in serum, namely, aspartate transaminase (AST), creatine kinase (CK), creatine kinase‐MB isoenzyme (CK‐MB), and lactate dehydrogenase isoenzyme 1 (LDH1), were obtained in accordance with the instructions of the relevant assay kits (Servicebio, China).

### Assessment of Nonheme Iron Level in the Myocardium

Tissue nonheme iron levels were measured with a Prussian blue iron stain (enhanced with DAB) kit. A paraffin‐embedded tissue section was first removed. After it reached room temperature, the sample was dewaxed and rehydrated. A Prussian blue working solution was prepared according to the kit instructions and incubated with the samples at 37 °C for 20 min. Next, the slices were removed from the dye and washed in PBS three times, and then, dye solution was added dropwise to samples, which were incubated for 3 min. The slices were soaked in distilled water for 10 min and dehydrated with an ethanol gradient. Next, xylene was applied to the slices, which were then sealed with neutral gum before observation via light microscopy. Finally, nonheme iron was quantified using ImageJ software.

### Assessments of the Lipid Peroxidation Level in Mice: C11‐BODIPY 581/591 Dyes Tissue

The frozen sections of myocardium were incubated with C11‐BODIPY 581/591 for 0.5 h. Cells were labeled with DAPI after fixation and then imaged by CLSM.

### Serological Measurements of Lipid Peroxidation Markers

The levels of GPX4 and MDA in mouse serum were measured by using kits following the manufacturer's instructions. In addition, the 4‐HNE level in serum was measured using an ELISA kit. Standard and sample wells were prepared, and 50 µL of standard at various concentrations was added to the wells, and 10 µL of sample was mixed with 40 µL of sample diluent and then added to the sample wells. This process was followed by the addition of 0.1 mL of horseradish peroxidase (HRP)‐conjugated antibody to each well. The plate was washed five times, and 50 µL of Substrate A and B was added after the plate was sealed with a membrane, then, the plate was incubated in a 37 °C water bath for 1 h. Next, the plate was incubated at 37 °C in the dark for 15 min. The optical density (OD) was read at 450 nm after the addition of a termination solution. Finally, the concentration results were measured on the basis of a standard curve.

### Immunohistochemistry

Frozen sections were allowed to equilibrate at room temperature for 15 min, fixed with 4% polyformaldehyde for 10 min, and then washed in PBS. Adherent tissue samples were removed from the substrate by treating samples with 0.1% Triton‐X100 for 10 min and then washing them with PBS. The samples were cultured overnight at 4 °C with a diluted primary antibody, namely, an anti‐GPX4 mouse monoclonal antibody (mAb) (Servicebio, China). After washing with PBS, the samples were then incubated with Cy3/FITC‐conjugated secondary antibodies for 1.5 h at room temperature. Finally, the sections were treated with an anti‐fade mounting medium containing a DAPI stain. The fluorescence images were acquired using a slice‐scanning system.

### Measurement of the LD Content in Myocardial Tissue: Oil Red O Staining

The saturated Oil Red O solution prepared with isopropanol was mixed with distilled water in a 6:4 ratio. This solution was placed in a 60–70°C water bath for 0.5 h, cooled naturally, and filtered through qualitative filter paper to obtain an Oil red O working solution. The frozen myocardium sections were removed from the −20 °C freezer, allowed to stand at room temperature for 5–10 min to reach room temperature, and gently immersed in working solution for 8–10 min. The slices were removed from the solution, allowed to stand for 3 s, and then submerged in two cylinders containing 60% isopropanol for 3–5 s. The slices were then stained with hematoxylin, washed, and then sealed with glycerol–gelatin. Photomicrographs were captured using an Eclipse microscope (Nikon Ni‐E).

### LD Staining

Frozen myocardial sections were treated with **BTDPP**, **PMBTDP**, and **TPABTBP** for 0.5 h respectively, and the cells were labeled with DAPI after fixation. Finally, laser confocal microscopy imaging was performed to determine the LD content through ImageJ.

### In Vitro Fluorescence Imaging (FI) of the Heart

For evaluating the biodistribution of **TPABTBP**, mice (*n =* 3 per independent experiment) were injected with **TPABTBP** (2 mg kg^−1^) via the tail vein. Following this, the mice were sacrificed and dissected at 1, 3, 6, 12, and 24 h post‐injection. Main organs (heart, liver, spleen, lungs, and kidneys) were removed for in vitro FI on an IVIS spectrum system (PerkinElmer, USA).

To assess the LD content, model mice of MIRI (*n =* 5 per independent experiment) were injected with **TPABTBP** (2 mg kg^‐1^) through the tail vein after reperfusion for 0, 3, 6, and 18 h. They were sacrificed 6 h later, and the hearts were removed for in vitro FI on an IVIS spectrum system. The sham operation group received the same dose and was imaged 6 h later. We then sliced each heart immediately into 4–5 short‐axis sections and imaged them again.

In all FI experiments, fluorescence reflectance images were obtained employing 480 nm excitation and 670 nm emission filter settings, with a 6 s exposure time. White light images were acquired for each FI dataset, and the fluorescence signal was quantified using Living Image (version 4.4).

### Biosafety in Vitro

First, 6×10^4^ H9c2 cells per well were uniformly inoculated in 96‐well plates, and then, different concentrations of TBABTBP (0, 5, 10, 30, and 120 µM) diluted with DMEM containing 10% FBS was added. These cells were cocultured with cardiomyocytes in a volume of 0.1 mL per well for 12 h. Then, CCK8 reagent (HyClone, China) was added and incubated with the cells for 1.5 h. The final OD580 values were read using an enzyme marker. The percentage of viable cells was calculated, and the difference in cell viability between the treatment and control groups was determined. In addition, 200 µL of erythrocytes were extracted from healthy mice and placed these cells onto plates. Then, the erythrocytes were cultured with PBS (negative control), Triton‐X100 (positive control), and various concentrations of **TPABTBP** at 37 °C for 6 h. The cells were then centrifuged, and the supernatants from each sample were analyzed by determining the OD at 414 nm using a multifunctional microplate detection platform (TECAN SPARK, Switzerland). The hemolysis rate (%) was calculated as follows: hemolysis rate (%) = (OD sample‐OD negative)/ (OD positive‐OD negative) × 100%. We also collected 1 mL of erythrocytes from healthy mice; diluted the cells in saline at a ratio of 1:3; dispersed them in Eppendorf (EP) tubes; mixed them with PBS, Triton X‐100, or probes; and incubated them for 2 h before obtaining images.

### Biosafety in Vivo

C57BL/6J mice were randomly assigned to a PBS, **TPABTBP** (2 mg kg^−1^), and CQ (30 mg kg^−1^) group. The mice in the PBS and **TPABTBP** groups (*n =* 5) were injected in the tail vein every 48 h intervals three times. On day 7, blood was collected from the mice, which were then sacrificed and dissected. Samples were collected for routine H&E evaluation of the kidney, liver, heart, spleen, and lungs. The mice in the CQ group were given a single intraperitoneal injection, sacrificed, and dissected the next day. The processes performed with the CQ group were the same as those performed with the other two groups. A portion of the collected whole blood was used for routine blood tests, while the other portion was centrifuged, and the serum was collected to evaluate the AST, urea nitrogen (BUN), creatinine (CREA), and alanine aminotransferase (ALT) levels using the respective assay kits.

### Statistical Analysis

Data analysis and graph preparation were performed using GraphPad Prism software. All the summarized data were presented as the mean ± standard deviation (SD). Blinding or randomization was used. Student's t‐tests were performed to compare two groups and one‐way ANOVAs were performed for comparisons among three or more groups. Differences were considered to be significant when the *p‐*value was < 0.05.

## Conflict of Interest

The authors declare no conflict of interest.

## Supporting information

Supporting Information

Supplemental Video 1

## Data Availability

The data that support the findings of this study are available from the corresponding author upon reasonable request.

## References

[1] G. Heusch , B. Gersh , Eur. Heart J. 2017, 38, 774. 27354052 10.1093/eurheartj/ehw224

[2] a) D. J. Hausenloy , D. M. Yellon , Nat. Rev. Cardiol. 2016, 13, 193; 26843289 10.1038/nrcardio.2016.5

[3] a) D. J. Hausenloy , D. M. Yellon , J. Clin. Invest. 2013, 123, 92; 23281415 10.1172/JCI62874 PMC3533275

[4] a) Z. Wang , Nat. Cell Biol. 2023, 25, 515; 10.1038/s41556-023-01129-5 37059879

[5] S. J. Dixon , K. M. Lemberg , M. R. Lamprecht , R. Skouta , E. M. Zaitsev , C. E. Gleason , D. N. Patel , A. J. Bauer , A. M. Cantley , W. S. Yang , B. Morrison 3rd , B. R. Stockwell , Cell 2012, 149, 1060. 22632970 10.1016/j.cell.2012.03.042 PMC3367386

[6] X. Fang , H. Wang , D. Han , E. Xie , X. Yang , J. Wei , S. Gu , F. Gao , N. Zhu , X. Yin , Proc. Natl Acad. Sci 2019, 116, 2672. 30692261 10.1073/pnas.1821022116 PMC6377499

[7] H. D. Miyamoto , M. Ikeda , T. Ide , T. Tadokoro , S. Furusawa , K. Abe , K. Ishimaru , N. Enzan , M. Sada , T. Yamamoto , S. Matsushima , T. Koumura , K. Yamada , H. Imai , H. Tsutsui , JACC Basic Tranl Sci. 2022, 7, 800. 10.1016/j.jacbts.2022.03.012 PMC9436815 36061338

[8] a) X. Yang , Y. Chen , J. Guo , J. Li , P. Zhang , H. Yang , K. Rong , T. Zhou , J. Fu , J. Zhao , Adv. Sci. 2023, 10, 2207216; 10.1002/advs.202207216 PMC10161035 36951540

[9] a) T. Petan , Rev. Physiol. Biochem. Pharm. 2020, 185, 53; 10.1007/112_2020_51 33074407

[10] a) I. Berlman , Handbook of Fluorescence Spectra of Aromatic Molecules, Elsevier, The Netherlands 1971;

[11] a) G. Lv , B. Cui , H. Lan , Y. Wen , A. Sun , T. Yi , Chem. Commun. 2015, 51, 125; 10.1039/c4cc07656g 25384304

[12] A. Loudet , K. Burgess , Chem. Rev. 2007, 107, 4891. 17924696 10.1021/cr078381n

[13] a) J. Qian , B. Z. Tang , Chem. Commun. 2017, 3, 56;

[14] a) P. Tan , W. Zhuang , S. Li , J. Zhang , H. Xu , L. Yang , Y. Liao , M. Chen , Q. Wei , Chem. Commun. 2021, 57, 1046; 10.1039/d0cc07336a 33409527

[15] H. F. Su , Q. C. Peng , Y. U. Liu , T. Xie , P. P. Liu , Y. C. Cai , W. Wen , Y. H. Yu , K. Li , S. Q. Zang , Biomaterials 2022, 288, 121691. 35948493 10.1016/j.biomaterials.2022.121691

[16] a) Y. Zhao , W. Shi , X. Li , H. Ma , Chem. Commun. 2022, 58, 1495; 10.1039/d1cc05717k 35019910

[17] a) Z. Zheng , H. Liu , S. Zhai , H. Zhang , G. Shan , R. T. Kwok , C. Ma , H. H. Sung , I. D. Williams , J. W. Lam , Chem. Sci. 2020, 11, 2494; 34084415 10.1039/c9sc06441a PMC8157451

[18] a) X. Zheng , W. Zhu , F. Ni , H. Ai , S. Gong , X. Zhou , J. L. Sessler , C. Yang , Chem. Sci. 2019, 10, 2342; 30881662 10.1039/c8sc04462g PMC6385674

[19] Y. Dong , J. W. Lam , A. Qin , J. Sun , J. Liu , Z. Li , J. Sun , H. H. Sung , I. D. Williams , H. S. Kwok , Chem. Commun. 2007, 21, 3255. 10.1039/b704794k 17668092

[20] X. Zheng , Q. Peng , L. Zhu , Y. Xie , X. Huang , Z. J. N. Shuai , Nanoscale 2016, 8, 15173. 27417250 10.1039/c6nr03599j

[21] M. Jiang , X. Gu , J. W. Y. Lam , Y. Zhang , R. T. K. Kwok , K. S. Wong , B. Z. Tang , Chem. Sci. 2017, 8, 5440. 28970923 10.1039/c7sc01400g PMC5609514

[22] a) Q. Li , Y. Li , T. Min , J. Gong , L. Du , D. L. Phillips , J. Liu , J. W. Lam , H. H. Sung , I. D. Williams , Angew. Chem., Int. Ed. 2020, 132, 9557; 10.1002/anie.201909706 31557385

[23] T. Cheng , Y. Zhao , X. Li , F. Lin , Y. Xu , X. Zhang , Y. Li , R. Wang , L. Lai , J. Chem. Inform. Model. 2007, 47, 2140. 10.1021/ci700257y 17985865

[24] a) T. H. Tsai , E. Chen , L. Li , P. Saha , H. J. Lee , L. S. Huang , G. S. Shelness , L. Chan , B. H. Chang , Autophagy 2017, 13, 1130; 28548876 10.1080/15548627.2017.1319544 PMC5529083

[25] a) M. Cao , T. Zhu , M. Zhao , F. Meng , Z. Liu , J. Wang , G. Niu , X. Yu , Anal. Chem. 2022, 94, 10676; 35853217 10.1021/acs.analchem.2c00964

[26] A. Barducci , G. Bussi , M. Parrinello , Phys. Rev. Lett. 2008, 100, 020603. 18232845 10.1103/PhysRevLett.100.020603

[27] S. H. Son , G. Park , J. Lim , C. Y. Son , S. S. Oh , J. Y. Lee , Nat. Commun. 2022, 13, 1. 35750680 10.1038/s41467-022-31400-6 PMC9232528

[28] M. Yue , B. Hu , J. Li , R. Chen , Z. Yuan , H. Xiao , H. Chang , Y. Jiu , K. Cai , B. Ding , EMBO J. 2023, 42, 112542. 10.15252/embj.2022112542 PMC10308351 37218505

[29] a) Y. Dai , X. Zhao , H. Ji , D. Zhang , P. Zhang , K. Xue , S. Misal , H. Zhu , Z. Qi , Chem. Eng. J. 2021, 410, 128186;

[30] a) W. K. Zhao , Y. Zhou , T. T. Xu , Q. Wu , Oxid. Med. Cell Longev. 2021, 2021, 9929687; 34725566 10.1155/2021/9929687 PMC8557044

[31] B. R. Stockwell , J. P. Friedmann Angeli , H. Bayir , A. I. Bush , M. Conrad , S. J. Dixon , S. Fulda , S. Gascon , S. K. Hatzios , V. E. Kagan , K. Noel , X. Jiang , A. Linkermann , M. E. Murphy , M. Overholtzer , A. Oyagi , G. C. Pagnussat , J. Park , Q. Ran , C. S. Rosenfeld , K. Salnikow , D. Tang , F. M. Torti , S. V. Torti , S. Toyokuni , K. A. Woerpel , D. D. Zhang , Cell 2017, 171, 273. 28985560 10.1016/j.cell.2017.09.021 PMC5685180

[32] a) A. P. Bailey , G. Koster , C. Guillermier , E. M. Hirst , J. I. MacRae , C. P. Lechene , A. D. Postle , A. P. Gould , Cell 2015, 163, 340; 26451484 10.1016/j.cell.2015.09.020 PMC4601084

[33] J. Wen , D. Wang , L. Cheng , D. Wu , L. Qiu , M. Li , Y. Xie , S. Wu , Y. Jiang , H. Bai , B. Xu , H. Lv , Cell Biol. Int. 2021, 45, 757. 33289183 10.1002/cbin.11513

[34] a) J. M. Ubellacker , A. Tasdogan , V. Ramesh , B. Shen , E. C. Mitchell , M. S. Martin‐Sandoval , Z. Gu , M. L. McCormick , A. B. Durham , D. R. Spitz , Nature 2020, 585, 113; 32814895 10.1038/s41586-020-2623-z PMC7484468

[35] a) L. Magtanong , P. J. Ko , M. To , J. Y. Cao , G. C. Forcina , A. Tarangelo , C. C. Ward , K. Cho , G. J. Patti , D. K. Nomura , Cell Chem. Biol. 2019, 26, 420; 30686757 10.1016/j.chembiol.2018.11.016 PMC6430697

[36] X. Jiang , B. R. Stockwell , M. Conrad , Nat. Rev. Mol. Cell Biol. 2021, 22, 266. 33495651 10.1038/s41580-020-00324-8 PMC8142022

[37] E. Jarc , T. Petan , Yale J. Biol. Med. 2019, 92, 435. 31543707 PMC6747940

[38] R. Unno , T. Kawabata , K. Taguchi , T. Sugino , S. Hamamoto , R. Ando , A. Okada , K. Kohri , T. Yoshimori , T. Yasui , Autophagy 2020, 16, 709. 31257986 10.1080/15548627.2019.1635382 PMC7138204

[39] K. Kajiwara , H. Osaki , S. Greßies , K. Kuwata , J. H. Kim , T. Gensch , Y. Sato , F. Glorius , S. Yamaguchi , M. Taki , Nat. Commun. 2022, 13, 2533. 35534485 10.1038/s41467-022-30153-6 PMC9085894

[40] a) C. Burman , N. T. Ktistakis , Semin. Immunopathol. 2010, 32, 397; 20740284 10.1007/s00281-010-0222-z

[41] a) Y. A. Wen , X. Xing , J. W. Harris , Y. Y. Zaytseva , M. I. Mitov , D. L. Napier , H. L. Weiss , B. M. Evers , T. J. Gao , Cell Death Dis. 2017, 8, e2593; 28151470 10.1038/cddis.2017.21 PMC5386470

[42] a) M. Mauthe , I. Orhon , C. Rocchi , X. Zhou , M. Luhr , K. J. Hijlkema , R. P. Coppes , N. Engedal , M. Mari , F. Reggiori , Autophagy 2018, 14, 1435; 29940786 10.1080/15548627.2018.1474314 PMC6103682

[43] Y. Han , X. Chu , L. Cui , S. Fu , C. Gao , Y. Li , B. Sun , Drug Deliv 2020, 27, 502. 32228100 10.1080/10717544.2020.1745328 PMC7170363

[44] X. Fang , Z. Cai , H. Wang , D. Han , Q. Cheng , P. Zhang , F. Gao , Y. Yu , Z. Song , Q. Wu , P. An , S. Huang , J. Pan , H. Z. Chen , J. Chen , A. Linkermann , J. Min , F. Wang , Circ. Res. 2020, 127, 486. 32349646 10.1161/CIRCRESAHA.120.316509

[45] Y. Santin , L. Fazal , Y. Sainte‐Marie , P. Sicard , D. Maggiorani , F. Tortosa , Y. Y. Yucel , L. Teyssedre , J. Rouquette , M. Marcellin , C. Vindis , J. C. Shih , O. Lairez , O. Burlet‐Schiltz , A. Parini , F. Lezoualc'h , J. Mialet‐Perez , Cell Death Differ. 2020, 27, 1907. 31819159 10.1038/s41418-019-0470-y PMC7244724

[46] W. S. Yang , R. SriRamaratnam , M. E. Welsch , K. Shimada , R. Skouta , V. S. Viswanathan , J. H. Cheah , P. A. Clemons , A. F. Shamji , C. B. Clish , Cell 2014, 156, 317. 24439385 10.1016/j.cell.2013.12.010 PMC4076414

[47] S. Wang , X. Li , S. Y. Chong , X. Wang , H. Chen , C. Chen , L. G. Ng , J. W. Wang , B. Liu , Adv. Mater. 2021, 33, 2007490. 10.1002/adma.202007490 33576084

[48] a) Y. Sun , M. Ding , X. Zeng , Y. Xiao , H. Wu , H. Zhou , B. Ding , C. Qu , W. Hou , A. Er‐Bu , Chem. Sci. 2017, 8, 3489; 28507722 10.1039/c7sc00251c PMC5418643

[49] Q. Lu , W. Li , Z. Li , Z. Chen , W. Fu , Q. Jiang , S. Ding , Int. J. Clin. Exp. Pathol. 2019, 12, 2639. 31934092 PMC6949570

[50] B. Hess , C. Kutzner , D. van der Spoel , E. Lindahl , J. Chem. Theory Comput. 2008, 4, 435. 26620784 10.1021/ct700301q

[51] A. D. MacKerell , D. Bashford , M. Bellott , R. L. Dunbrack , J. D. Evanseck , M. J. Field , S. Fischer , J. Gao , H. Guo , S. Ha , D. Joseph‐McCarthy , L. Kuchnir , K. Kuczera , F. T. Lau , C. Mattos , S. Michnick , T. Ngo , D. T. Nguyen , B. Prodhom , W. E. Reiher , B. Roux , M. Schlenkrich , J. C. Smith , R. Stote , J. Straub , M. Watanabe , J. Wiorkiewicz‐Kuczera , D. Yin , M. Karplus , J. Phys. Chem. B 1998, 102, 3586. 24889800 10.1021/jp973084f

[52] K. Vanommeslaeghe , E. Hatcher , C. Acharya , S. Kundu , S. Zhong , J. Shim , E. Darian , O. Guvench , P. Lopes , I. Vorobyov , A. D. Mackerell Jr. , J. Comput. Chem. 2010, 31, 671. 19575467 10.1002/jcc.21367 PMC2888302

[53] a) U. Essmann , L. Perera , M. L. Berkowitz , T. Darden , H. Lee , L. G. Pedersen , J. Chem. Phys. 1995, 103, 8577;

[54] S. Jo , T. Kim , V. G. Iyer , W. Im , J. Comput. Chem. 2008, 29, 1859. 18351591 10.1002/jcc.20945

[55] H. J. C. Berendsen , J. P. M. Postma , W. F. Van Gunsteren , A. DiNola , J. R. Haak , J. Chem. Phys. 1984, 81, 3684.

[56] B. Hess , H. Bekker , H. J. Berendsen , J. G. Fraaije , J. Comput. Chem. 1997, 18, 1463.

[57] W. F. Van Gunsteren , H. J. J. Berendsen , Mol. Simul. 1988, 1, 173.

[58] W. Zhao , S. Zhao , L. Li , X. Huang , S. Xing , Y. Zhang , G. Qiu , Z. Han , Y. Shang , D. E. Sun , C. Shan , R. Wu , L. Gu , S. Zhang , R. Chen , J. Xiao , Y. Mo , J. Wang , W. Ji , X. Chen , B. Ding , Y. Liu , H. Mao , B. L. Song , J. Tan , J. Liu , H. Li , L. Chen , Nat. Biotechnol. 2021, 40, 606. 34782739 10.1038/s41587-021-01092-2

