# Supplementary material for: A Membrane‐Targeting Aggregation‐Induced Emission Probe for Monitoring Lipid Droplet Dynamics in Ischemia/Reperfusion‐Induced Cardiomyocyte Ferroptosis
Source: Adv Sci (Weinh). 2024 May 2;11(26):2309907. doi: 10.1002/advs.202309907 (PMC11234465; doi:10.1002/advs.202309907)
Supplement: Supplementary file 1 — Supporting Information [file ADVS-11-2309907-s002.pdf]

ADVANCED SCIENCE
Open Access

Supporting Information

for *Adv. Sci.*, DOI 10.1002/advs.202309907

A Membrane-Targeting Aggregation-Induced Emission Probe for Monitoring Lipid Droplet Dynamics in Ischemia/Reperfusion-Induced Cardiomyocyte Ferroptosis

Yihui Wang, Yuan Song, Lingling Xu, Wuqi Zhou, Wenyuan Wang, Qiaofeng Jin, Yuji Xie, Junmin Zhang, Jing Liu, Wenqian Wu, He Li, Le Liang, Jing Wang, Yali Yang, Xiongwen Chen, Shuping Ge, Tang Gao, Li Zhang* and Mingxing Xie**

Supporting Information

A membrane-targeting aggregation-induced emission probe for monitoring lipid droplets dynamics during myocardial ischemia-reperfusion induced ferroptosis

Yihui Wang[#], Yuan Song[#], Lingling Xu, Wuqi Zhou, Wenyuan Wang, Qiaofeng Jin, Yuji Xie, Junmin Zhang, Jing Liu, Wenqian Wu, He Li, Le Liang, Jing Wang, Yali Yang, Xiongwen Chen, Shuping Ge, Tang Gao[*], Li Zhang[*], Mingxing Xie[*]

Table S1. Comparisons on the performance and applications of reported LDs fluorescent probes.

Probe structure	E_{ex}/E_{em} (nm)	Stokes shift (nm)	Imaging application	Ref
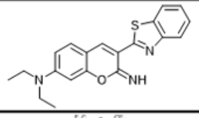	479/534	55	A549, 4T1, Foam Cell fatty liver tissues, atherosclerosis mice model	1
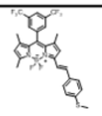	561/615	54	RAW264.7, HepG2 cells, atherosclerosis mice model	2
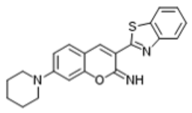	450/521	71	A549 cells, foam cells, atherosclerosis mice model	3
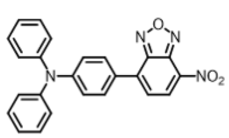	556/657	101	HeLa cells, RAW 264.7, foam cells hepatic tissues of mice with fatty livers, main aortic tree of the Arteriosclerosis mice	4
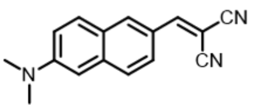	458/523	65	RAW 264.7, hepatic tissues of mice with fatty livers, main aortic tree of the Arteriosclerosis mice	5
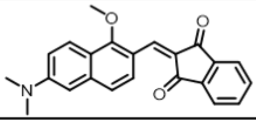	561/600	39	3T3-L1 preadipocyte cells, Fatty liver tissues	6
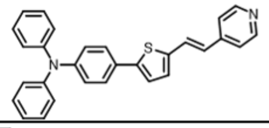	410/535	125	HeLa, A549, SMMC-7721 cells, fatty liver tissues, inflammatory living mice, cancer patient tissues	7
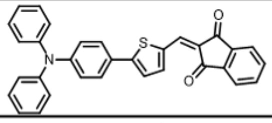	520/600	80	ccRCC tumor cells, 786-O cells, human ccRCC tumor tissues	8
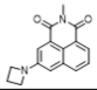	436/520	84	MCF-7 cells HeLa cells	9
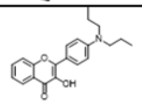	414/568	154	HepG2 cells	10
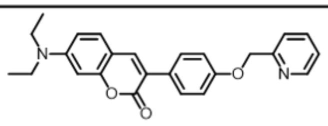	405/480	75	HeLa cells	11
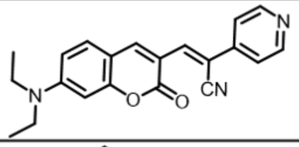	405/503	98	HeLa cells	12
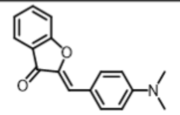	405/501	96	A549 cells	13

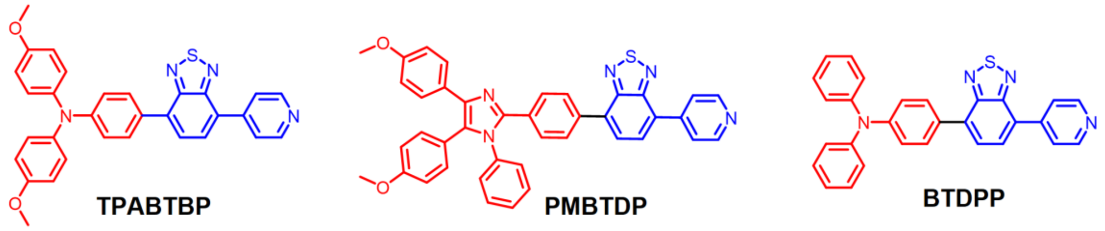

Figure S1. The structure of **TPABTBP**, **PMBTDP** and **BTDPP**.

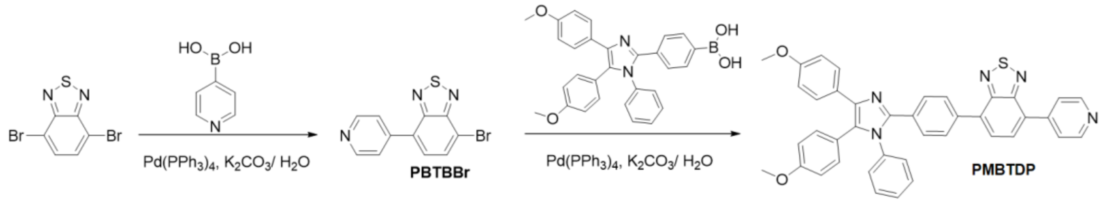

Scheme S1. Synthetic route of **PMBTDP**.

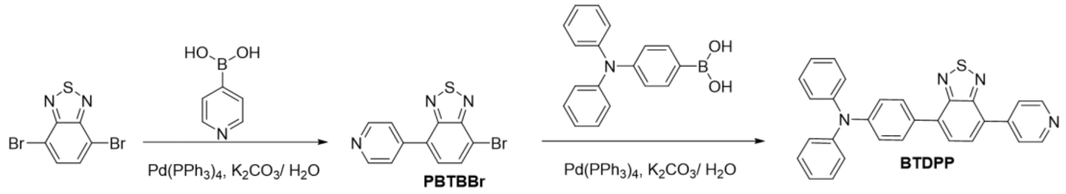

Scheme S2. Synthetic route of **BTDPP**.

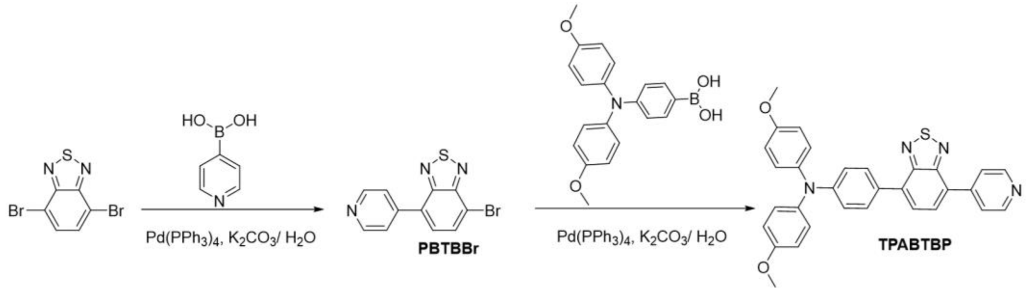

Scheme S3. Synthetic route of **TPABTBP.**

*Synthesis of compound **PBTBBr**:* Pyridine-4-boronic acid (200 mg, 1.62 mmol), 4,7-dibromo-benzo[c][1,2,5]thiadiazole (472.8 mg, 1.62 mmol), 250 mg potassium carbonate, and 20 mg $Pd(PPh_3)_4$ were added to a 100 mL round bottom flask equipped with a condenser. The resulting mixture was dissolved in 10 mL DMF and 2 mL water under nitrogen protection to obtain a solution, which was stirred (400 rpm) and refluxed at 80°C for 24 h. After cooling to room temperature (25°C), the reaction mixture was extracted three times with ethyl acetate under anhydrous conditions, and the organic phase was collected and dried over anhydrous sodium sulfate. The solvent was then removed under reduced pressure (vacuum degree of 0.1 Mpa) to obtain a crude product. The crude product was purified by silica gel column chromatography using petroleum ether/ethyl acetate (25:1, v/v) as the eluent to obtain solid **PBTBBr** (226.2 mg, 48%

yield). ^{1}H NMR (600 MHz, $CDCl_3$), δ(ppm): 8.78-8.79 (m, 2H), 7.98-7.99 (d, 1H), 7.86 - 7.87(d, 2H), 7.69-7.70 (d, 1H); ^{13}C NMR (150 MHz, $CDCl_3$), δ(ppm):153.96, 152.48, 150.22, 143.94, 132.12, 130.89, 128.92, 123.48, 115.42.

*Synthesis of compound **TPABTBP***: Compound **PBTBBr** (55.1 mg, 0.19 mmol) and compound (4-(bis(4-methoxyphenyl)amino)phenyl)boronic acid (66.3 mg, 0.19 mmol) were dissolved in 5 mL of DMF and stirred thoroughly (600 rpm) at room temperature (25°C). Subsequently, 1 mL of an aqueous solution containing 50 mg of K_2CO_3 was added to the reaction mixture, followed by the addition of 10 mg of the catalyst $Pd(PPh_3)_4$. The reaction mixture was refluxed under nitrogen protection at 80 °C for 12 hours. After TLC monitoring confirmed complete reaction, the solvent was removed by vacuum distillation (0.1MPa), and the product **TPABTBP** was obtained by silica gel column chromatography (petroleum ether/ethyl acetate, 25:1, v/v) with a yield of 32.8%.^{1}H NMR(600 MHz, $CDCl_3$), δ(ppm): 8.92- 8.93 (d, 2H), 8.58-8.57 (d, 2H), 8.07-8.09 (d, 1H), 7.88-7.90 (d, 2H), 7.83-7.85 (d, 1H), 7.16-7.17 (m, 4H), 7.05-7.06 (d, 2H), 6.88-6.90 (d, 4H), 3.83 (s, 6H); ^{13}C NMR(150 MHz, $CDCl_3$), δ(ppm): 156.63, 154.11, 153.19, 152.36, 150.13, 142.52, 139.92, 137.88, 130.87, 130.26, 127.47, 127.02, 125.83, 125.29, 124.72, 118.92, 114.91, 55.53. HRMS (ESI^+): calcd for $C_{31}H_{25}N_4O_2S^+$[M+H] $^+$: 517.1693; found: 517.1689.

*Synthesis of compound **PMBTDP***: Compound **PBTBBr** (150 mg, 0.52 mmol) and compound (4-(4,5-bis(4-methoxyphenyl)-1-phenyl-1H-imidazol-2-yl)phenyl)boronic acid (245.5 mg, 0.52 mmol) were dissolved in 5 mL of DMF and stirred thoroughly (600 rpm) at room temperature (25°C). Subsequently, 1 mL of an aqueous solution containing 80 mg of K_2CO_3 was added to the reaction mixture, followed by the addition of 20 mg of the catalyst $Pd(PPh_3)_4$. The reaction mixture was refluxed under nitrogen protection at 80°C for 12 hours. After TLC monitoring confirmed complete reaction, the solvent was removed by vacuum distillation (0.2 MPa), and the product **PMBTDP** was obtained by silica gel column chromatography with a yield of 32.8%.^{1}H NMR(600 MHz, $CDCl_3$), δ(ppm): 8.94 (s, 2H), 8.50 (s, 2H), 7.94-8.09 (m, 4H), 7.73-7.74 (m, 2H), 7.39-7.54 (m, 5H), 7.20-7.21 (m, 2H), 7.04-7.08 (m, 2H), 6.88-6.89 (d, 2H), 6.81-6.82 (d, 2H), 3.80-3.82 (m, 6H); ^{13}C NMR(150 MHz, $CDCl_3$), δ(ppm): 159.24, 158.50,

153.91, 153.54, 152.08, 150.09, 145.76,144.9, 142.69, 138.16, 137.29, 136.43, 135.88, 135.22, 134.42, 132.39, 130.34, 130.15, 129.26, 129.02, 128.93, 128.85, 128.79, 128.56, 128.54, 128.41, 127.78, 124.78, 124.02, 123.59, 123.48, 122.73, 115.92, 113.88, 55.22, 55.15. HRMS (ESI^+): calcd for $C_{40}H_{29}N_5O_2S$: 643.2042; found: 643.2053.

Synthesis of compound ***BTDPP*****:** Compound **PBTBBr** (100 mg, 0.22 mmol) and compound (4-diphenylamino)phenylboronic acid (63.6 mg, 0.22 mmol) were dissolved in 5 mL of DMF and stirred thoroughly (700 rpm) at room temperature (25°C). Subsequently, 1 mL of an aqueous solution containing 55 mg of K_2CO_3 was added to the reaction mixture, followed by the addition of 8 mg of the catalyst $Pd(PPh_3)_4$. The reaction mixture was refluxed under nitrogen protection at 80°C for 12 hours. After TLC monitoring confirmed complete reaction, the solvent was removed by vacuum distillation (0.3 MPa), and the product **BTDPP** was obtained by silica gel column chromatography with a yield of 32.8%. ^{1}HNMR (600 MHz, $CDCl_3$), δ(ppm): 8.77-8.78 (d, 2H), 7.95-7.96 (d, 2H), 7.88-7.90 (m, 3H), 7.79-7.80 (d, 1H), 7.29-7.32 (m, 4H), 7.07-7.10 (m, 2H); ^{13}CNMR (150 MHz, $CDCl_3$), δ(ppm): 154.07, 153.63, 150.12, 148.51, 147.33, 144.80, 134.74, 132.33, 130.91, 130.16, 130.06, 129.42, 129.26, 128.97, 128.85, 126.88, 125.10, 123.55, 122.55.

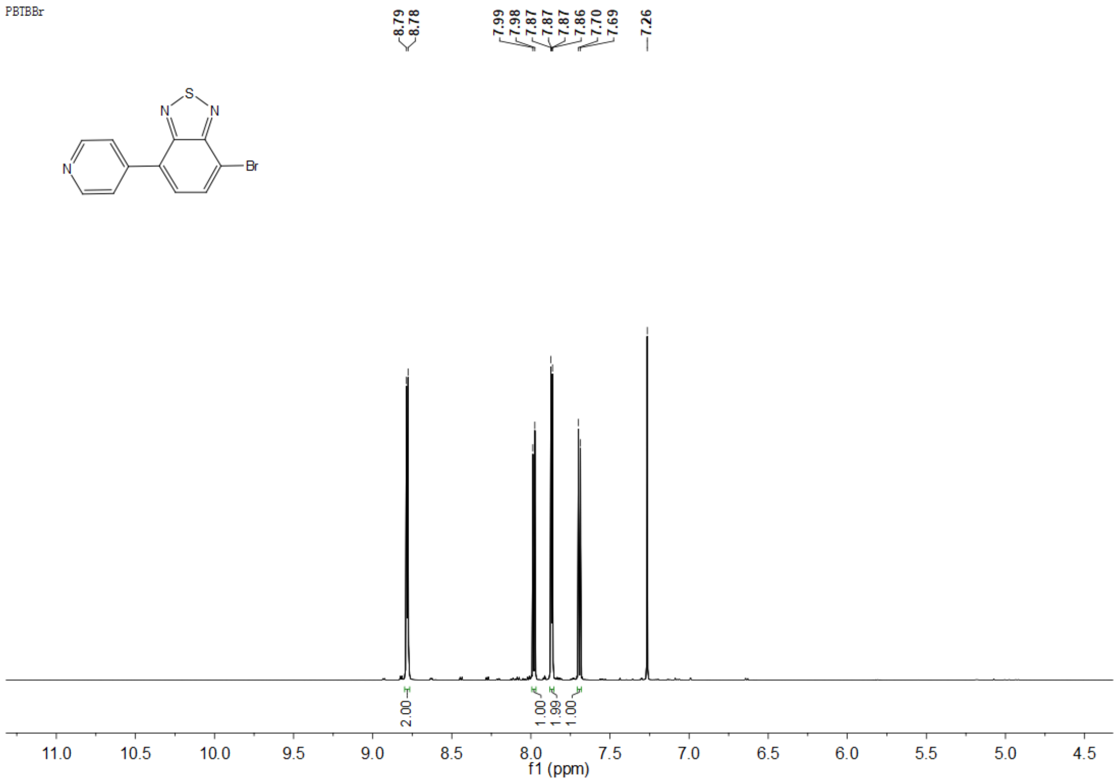

Figure S2. ^{1}HNMR spectrum of **PBTBBr**.

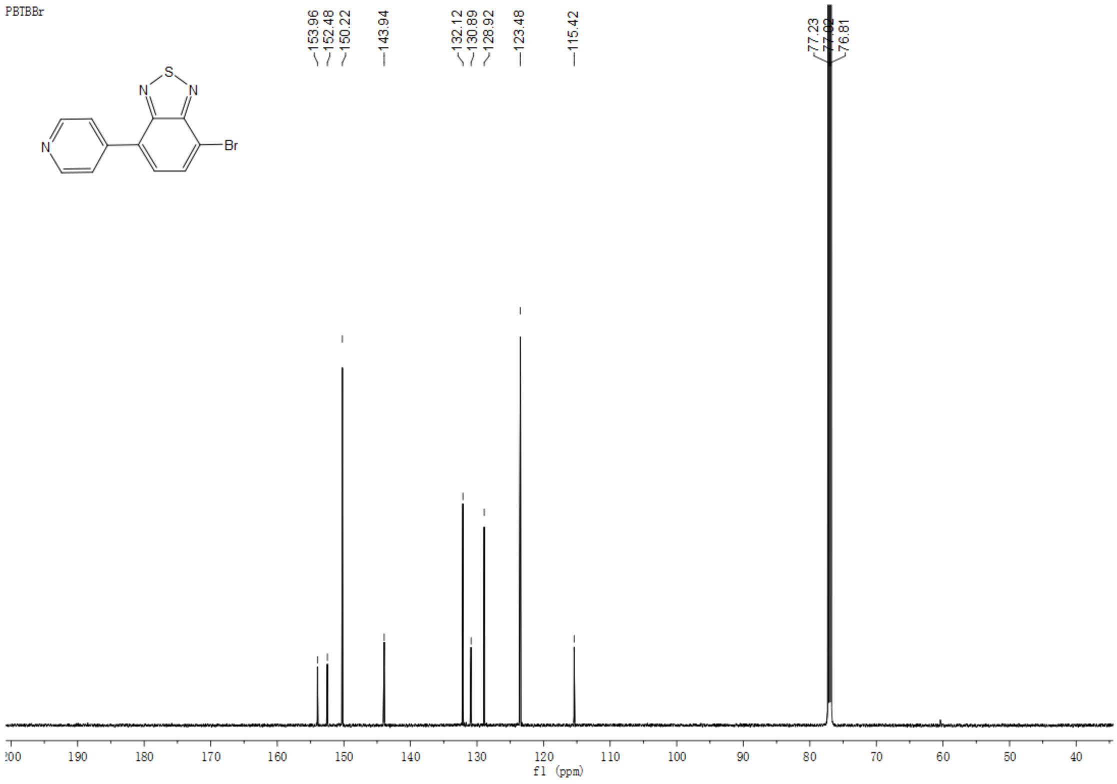

Figure S3. ^{13}CNMR spectrum of **PBTBBr**.

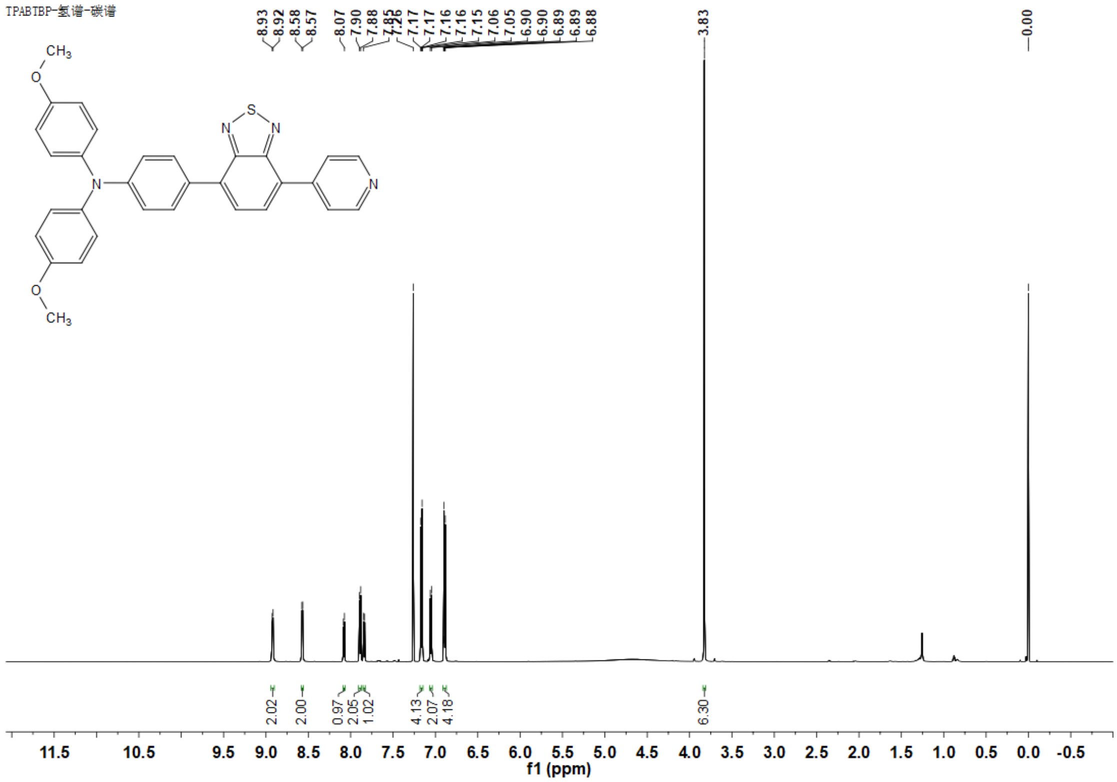

Figure S4. ^{1}HNMR spectrum of **TPABTBP.**

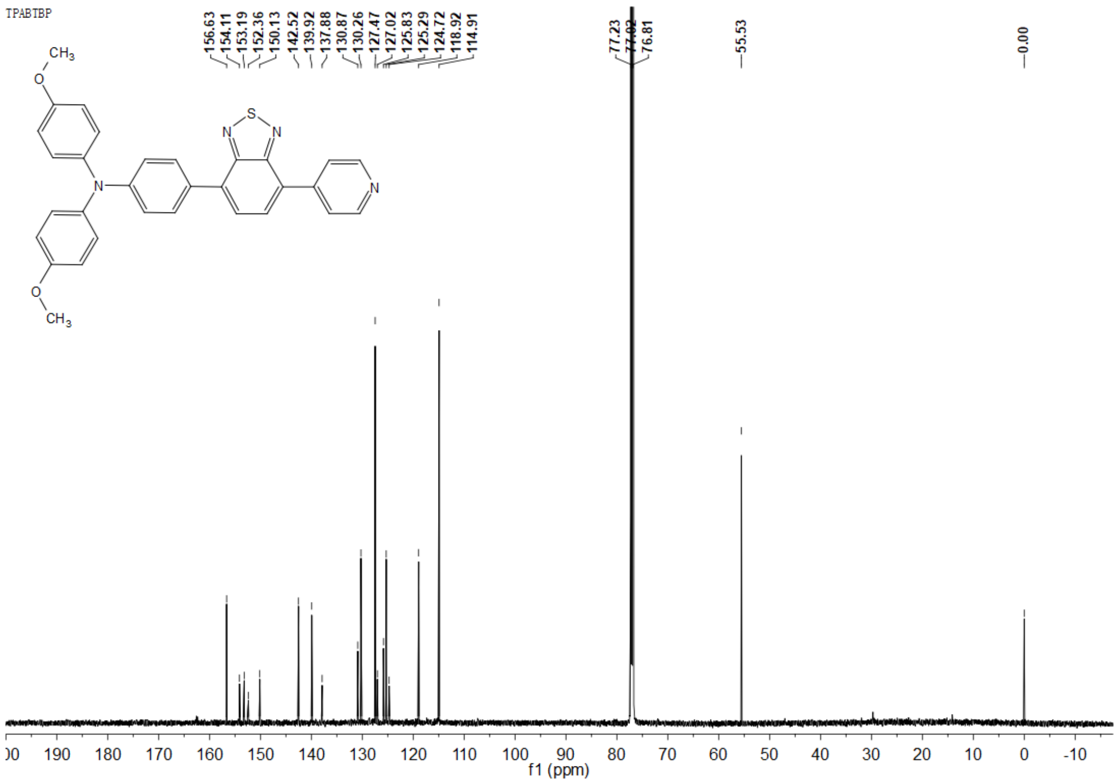

Figure S5. ^{13}CNMR spectrum of **TPABTBP.**

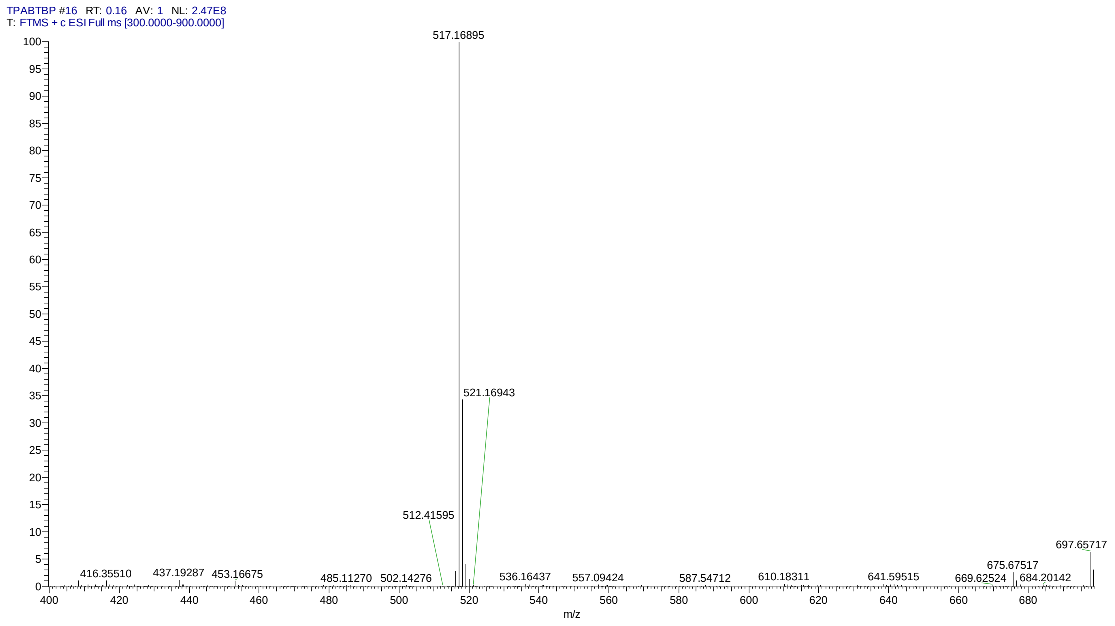

Figure S6. HRMS spectrum of **TPABTBP.**

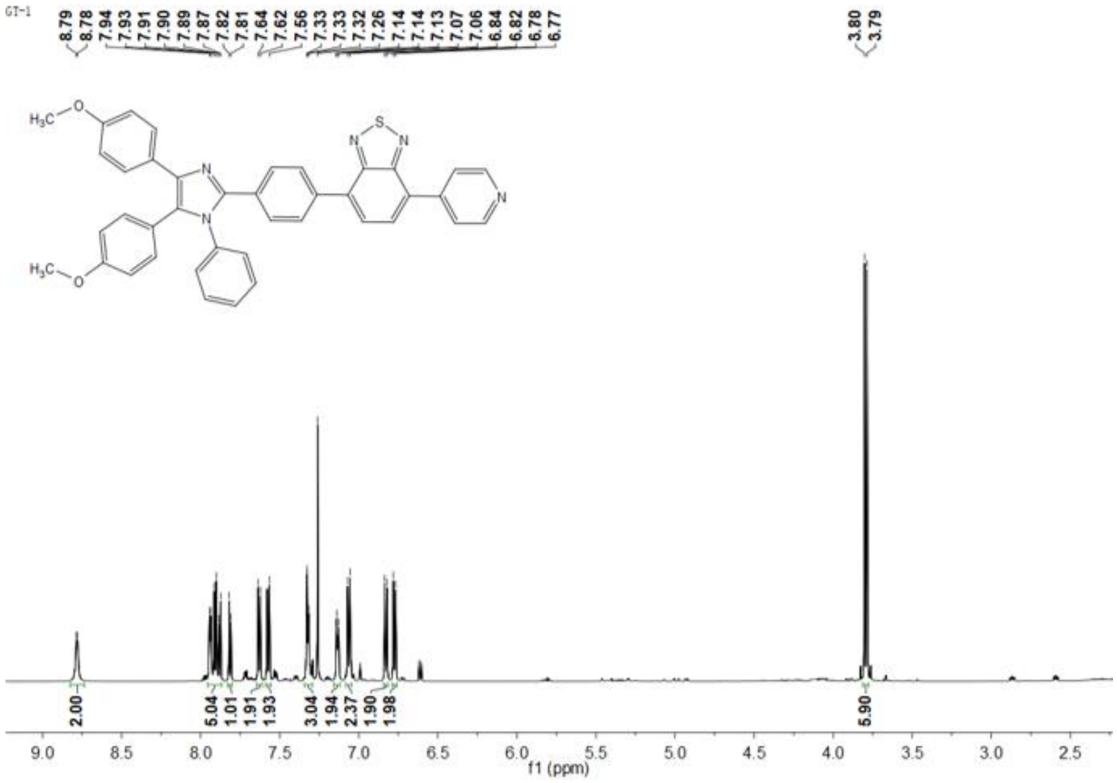

Figure S7. ^{1}HNMR spectrum of **PMBTDP.**

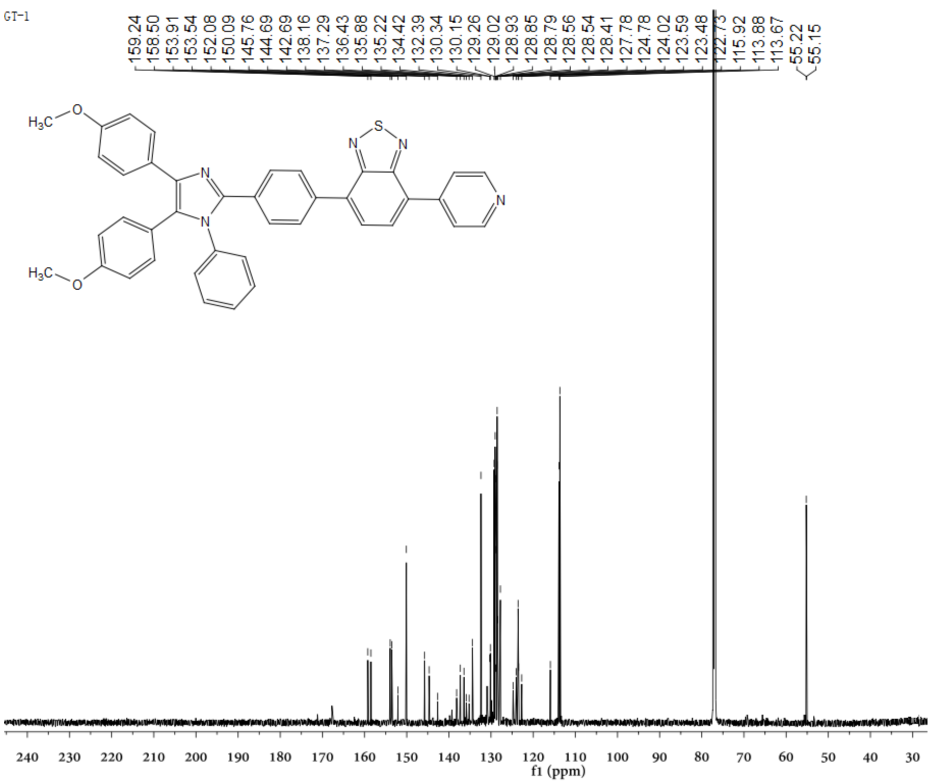

Figure S8. ^{13}CNMR spectrum of **PMBTDP.**

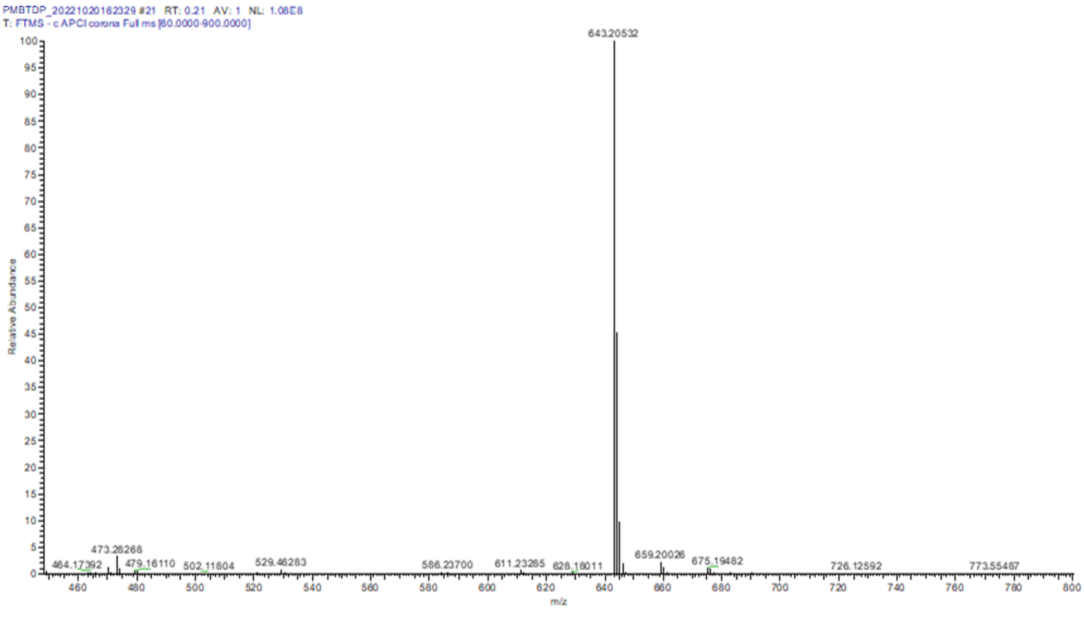

Figure S9. HRMS spectrum of **PMBTDP.**

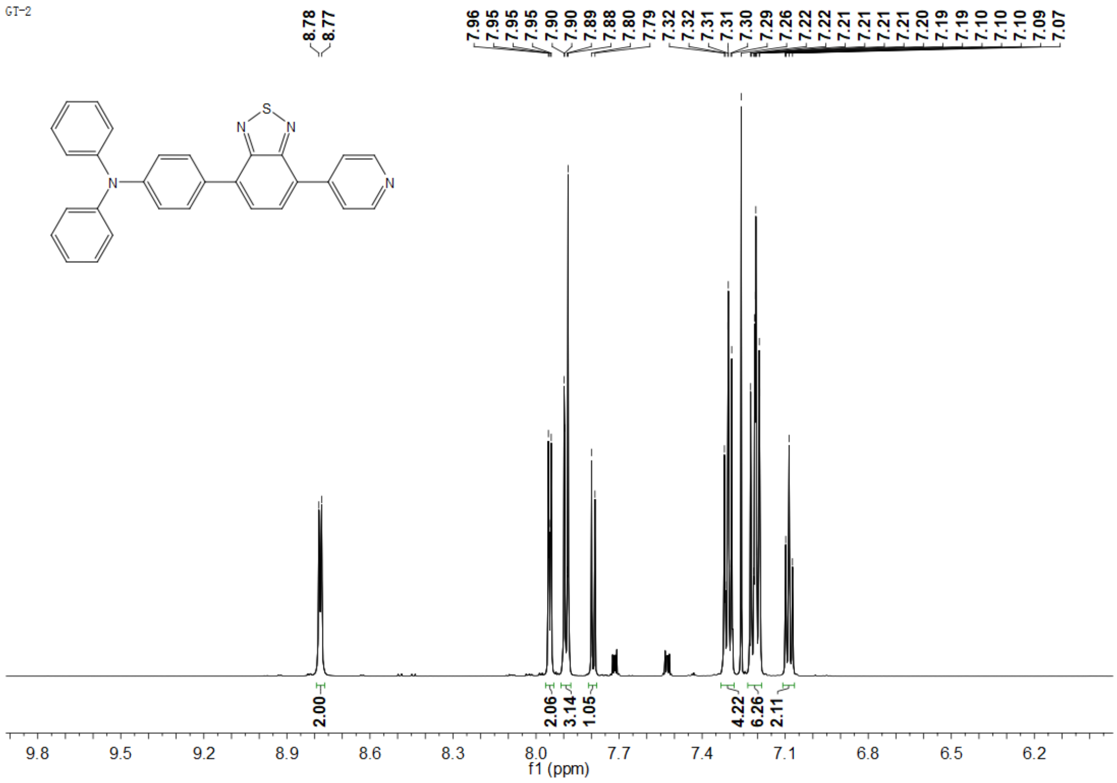

Figure S10. ^{1}HNMR spectrum of **BTDPP.**

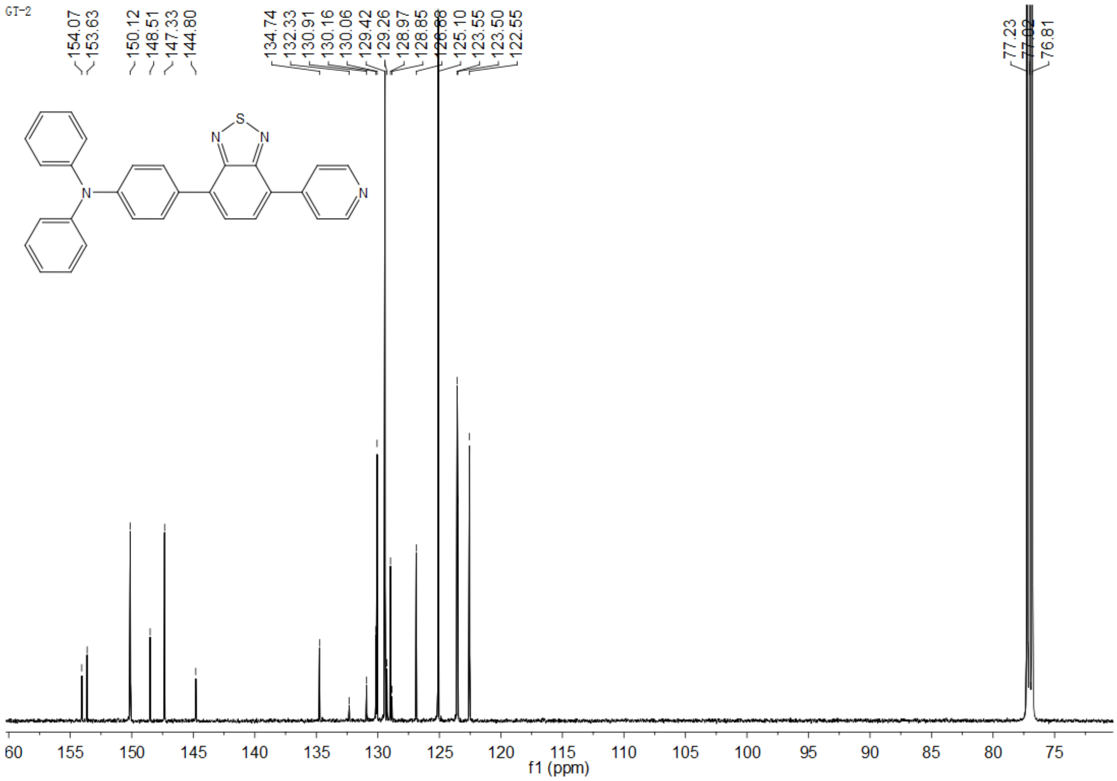

Figure S11. ^{13}CNMR spectrum of **BTDPP.**

Table S2. The fluorescence quantum yields of **TPABTBP**, **PMBTDP**, and **BTDPP** in various solvents.

	**TPABTBP**	**PMBTDP**	**BTDPP**
Toluene	50.90%	99.97%	98.95%
DMSO	<0.1%	<0.1%	2.08%
H_2O (containing 5% DMSO)	6.53%	12.86%	53.28%

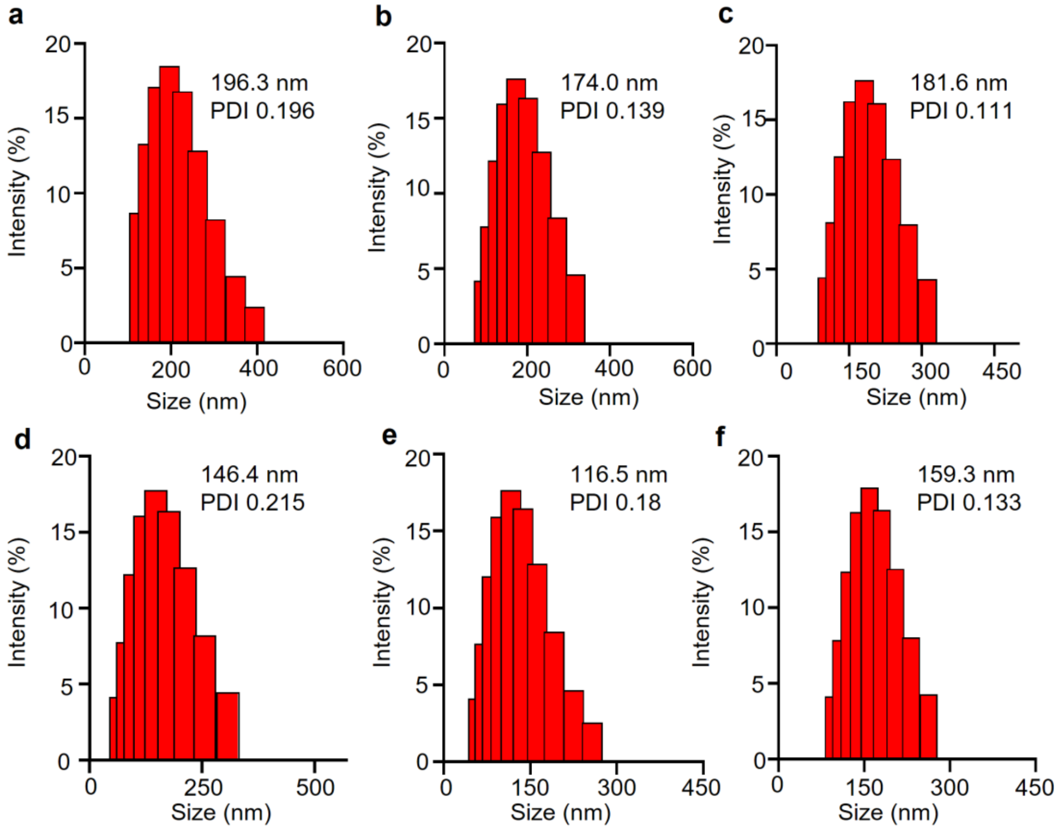

Figure S12. Dynamic light scattering (DLS) characterization results of the nanoaggregates of a, d) **TPABTBP**, b, e) **PMBTDP**, and c, f) **BTDPP** formed at 80% and 95% water fractions in the DMSO/H_2O mixtures.

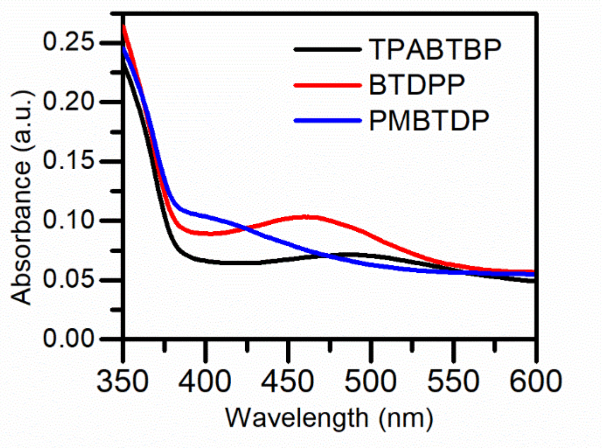

Figure S13. Absorption spectra of **TPABTBP**, **BTDPP** and **PMBTDP** in aqueous solutions (containing 5% DMSO).

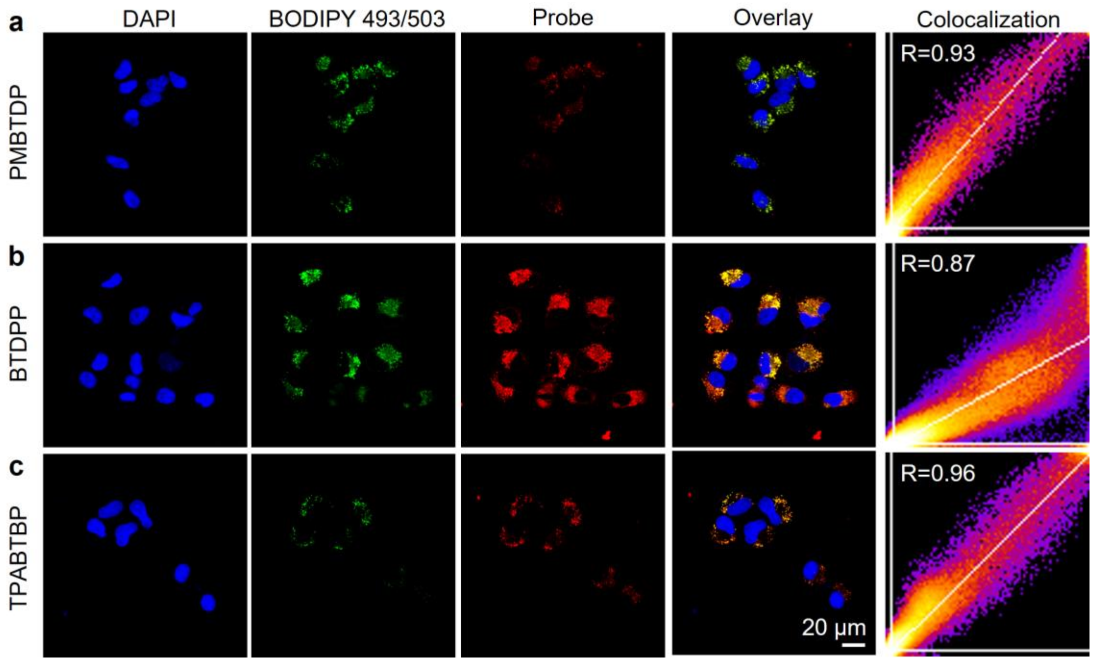

Figure S14. Confocal fluorescence scanning microscopy (CLSM) images of LD. a) CLSM images of HeLa cells co-stained with BODIPY 493/503 (5 μM) and **PMBTDP** (10 μM); b) CLSM images of HeLa cells co-stained with BODIPY 493/503 (5 μM) and **BTDPP** (10 μM); c) CLSM images of HeLa cells co-stained with BODIPY 493/503 (5 μM) and **TPABTBP** (10 μM). Scale bar: 20 μm.

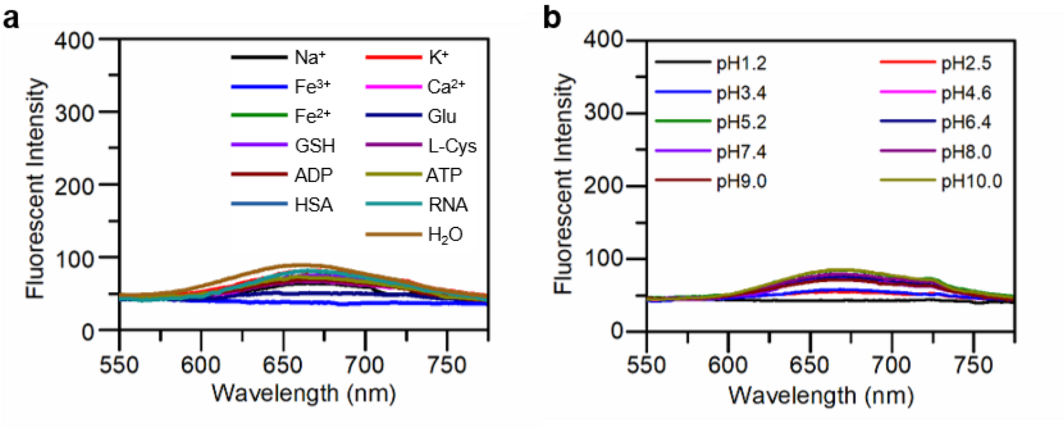

Figure S15. a) Fluorescence intensities of **TPABTBP** (10 μM) in ultrapure water or in the presence of Na^{+}, K^{+}, Fe^{2+}, Fe^{3+}, Ca^{2+}, glucose, HSA (5 mg/mL), ATP, ADP, GSH, Cys and RNA (0.23 nM); the concentration of the Na^{+}, K^{+}, Fe^{2+}, Fe^{3+} and Ca^{2+} are 5 mM, and the concentration of ATP, ADP, GSH, Cys are 1 mM; b) The fluorescence intensity of **TPABTBP** (10 μM) in solutions with different pH values. λex = 480 nm.

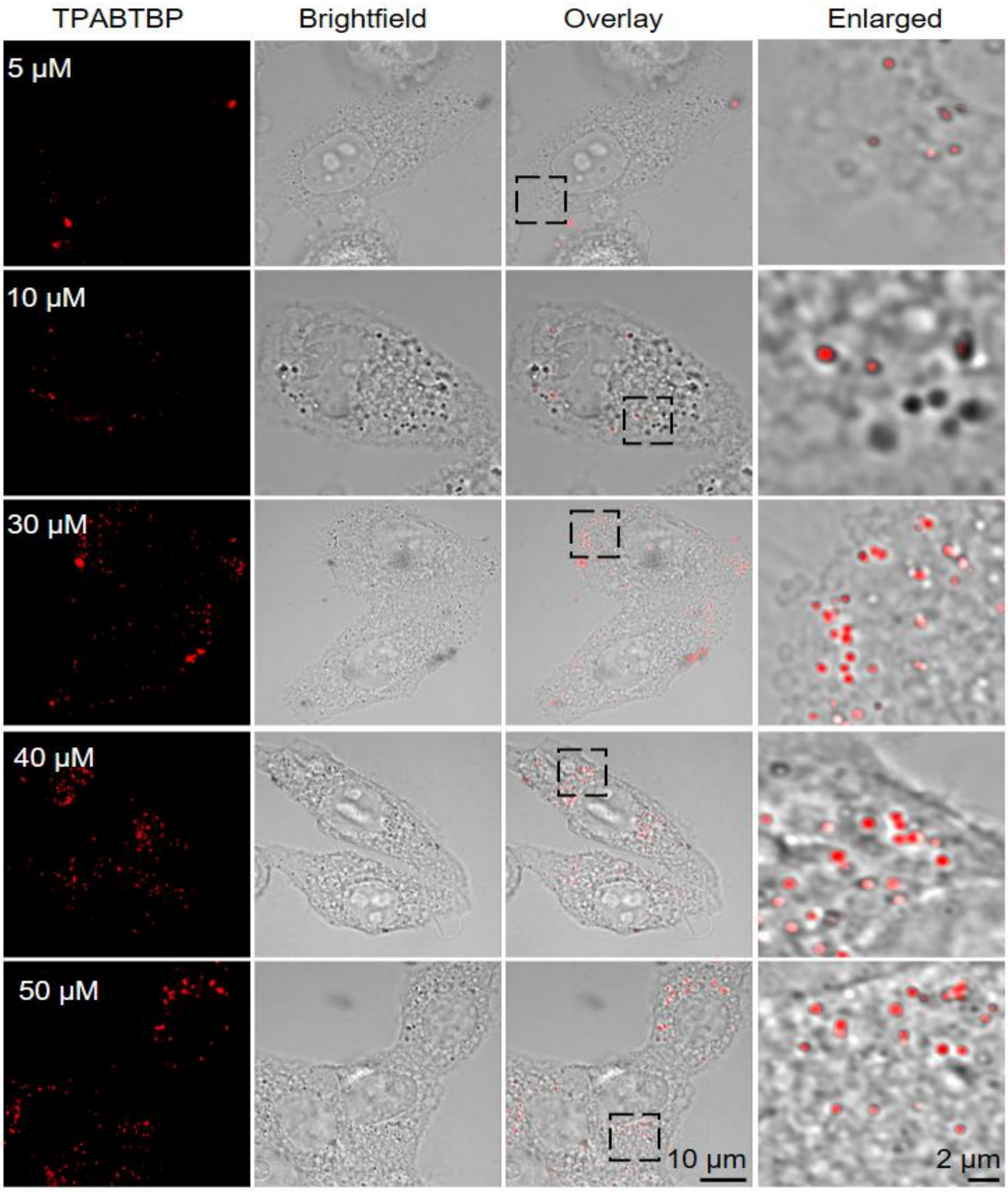

Figure S16. Imaging LDs in HepG2 cells with different concentrations of **TPABTBP** (5, 10, 30, 40 and 50 μM). Scale bar: 10 μm. Magnified image: 2 μm.

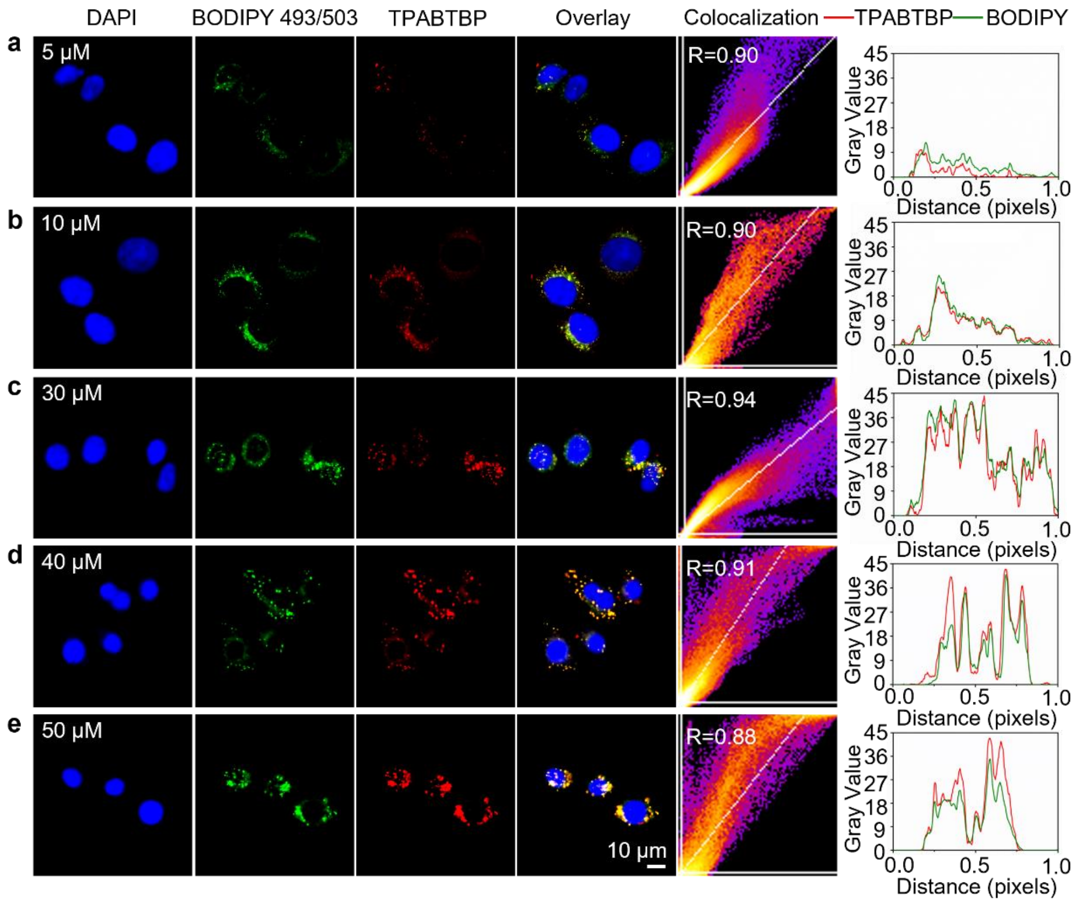

Figure S17. CLSM images of Hela cells co-stained with the same concentration of **TPABTBP** and BODIPY 493/503 (5, 10, 30, 40 and 50 μM). Scale :10 μm.

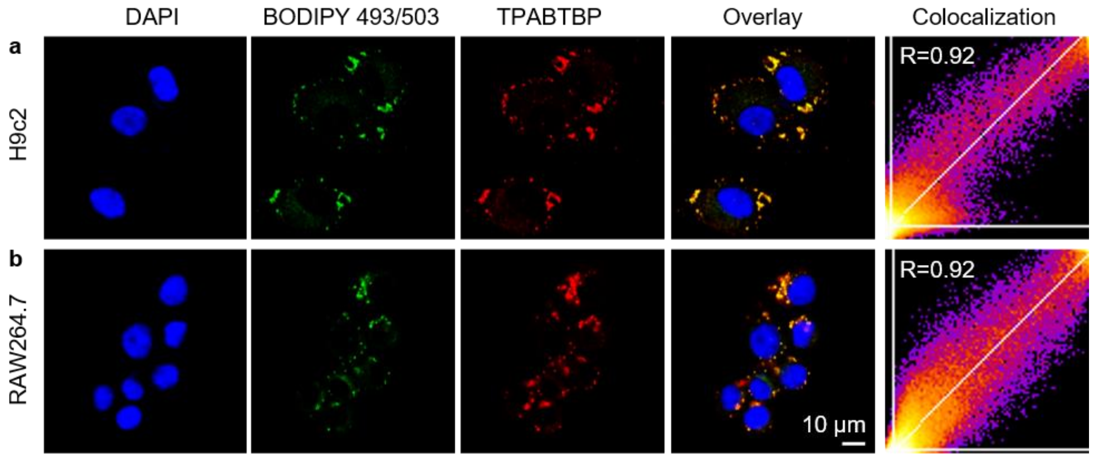

Figure S18. CLSM images of a) H9c2 cells or b) RAW264.7 cells co-stained with BODIPY 493/503 (5 μM) and **TPABTBP** (30 μM); Scale bar: 10 μm.

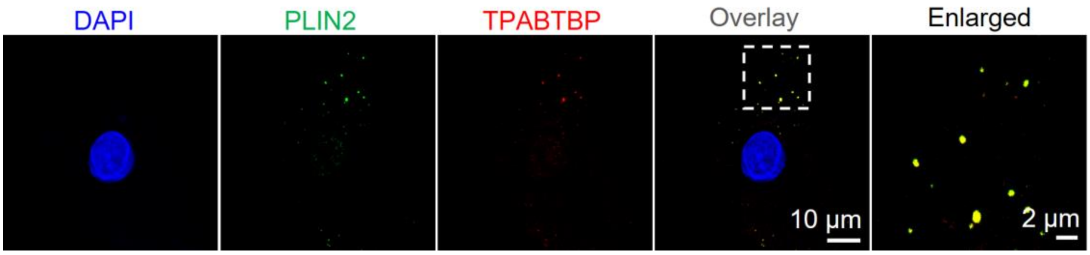

Figure S19. Immunofluorescence staining of H9c2 cells; The Alex 488-labeled antibody was used to stain a primary antibody targeting PLIN2; The LDs were stained with **TPABTBP**. Scale bar: 10 μm, Magnified image: 2 μm.

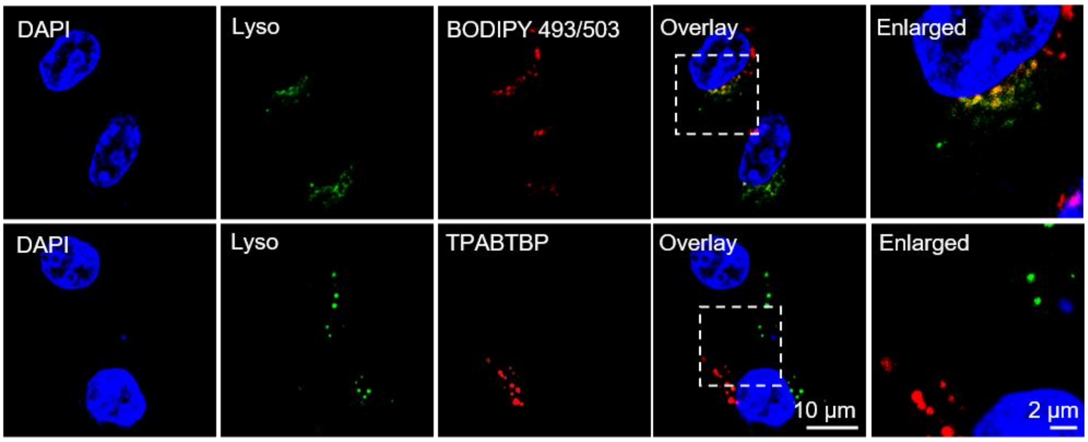

Figure S20. Fluorescence imaging analysis of H9c2 cells was conducted to assess the co-localization of BODIPY 493/503 and **TPABTBP** with LysoTracker™ Red DND-99. LysoTracker™ Red DND-99, $\lambda_{ex}/\lambda_{em}$=577/590 nm. BODIPY 493/503, $\lambda_{ex}/\lambda_{em}$=493/503 nm, and **TPABTBP**, $\lambda_{ex}/\lambda_{em}$=480/620 nm. Scale bar = 10 μm. Magnified image: 2 μm.

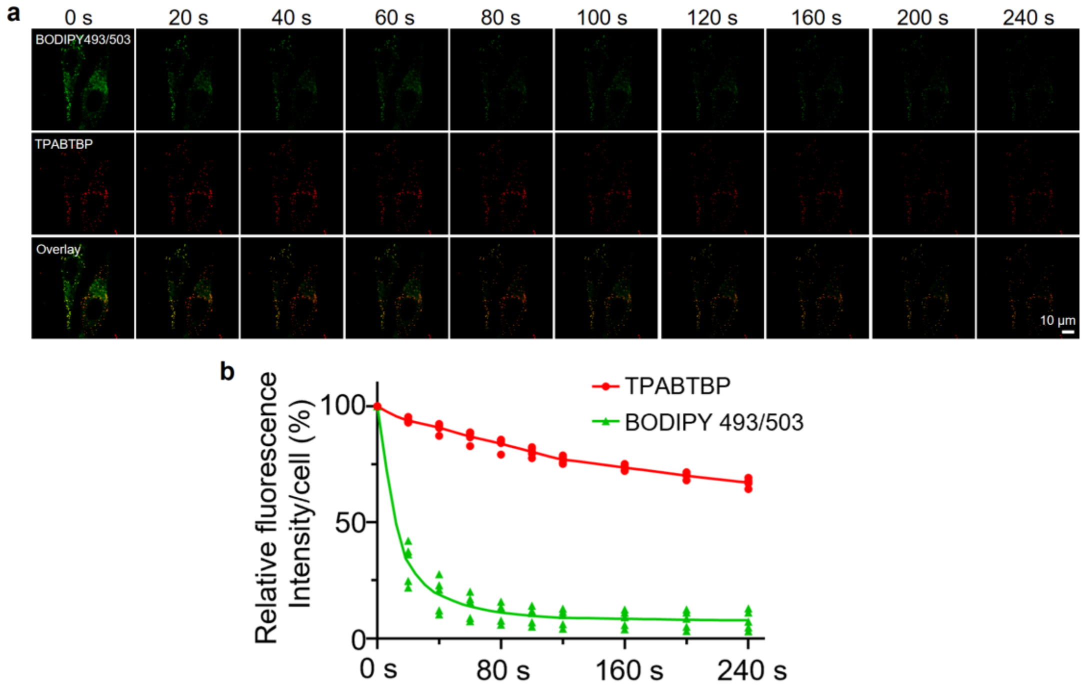

Figure S21. a) Confocal fluorescence imaging and b) fluorescence quantification analysis was conducted to evaluate the photostability of BODIPY 493/503 and **TPABTBP**. Scale bar: 10 μm; n=3.

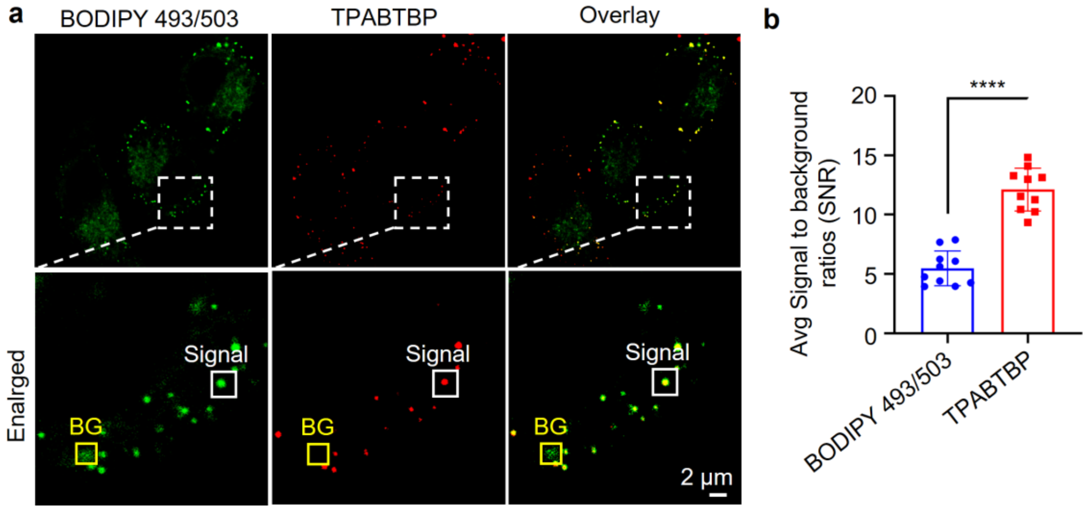

Figure S22. a) CLSM images of HepG2 cells stained with **TPABTBP** and BODIPY493/503, with signal and background (BG) regions marked for each channel; b) SNR of **TPABTBP** and BODIPY493/503. Scale bar: 2 μm; n=10, ****P<0.0001.

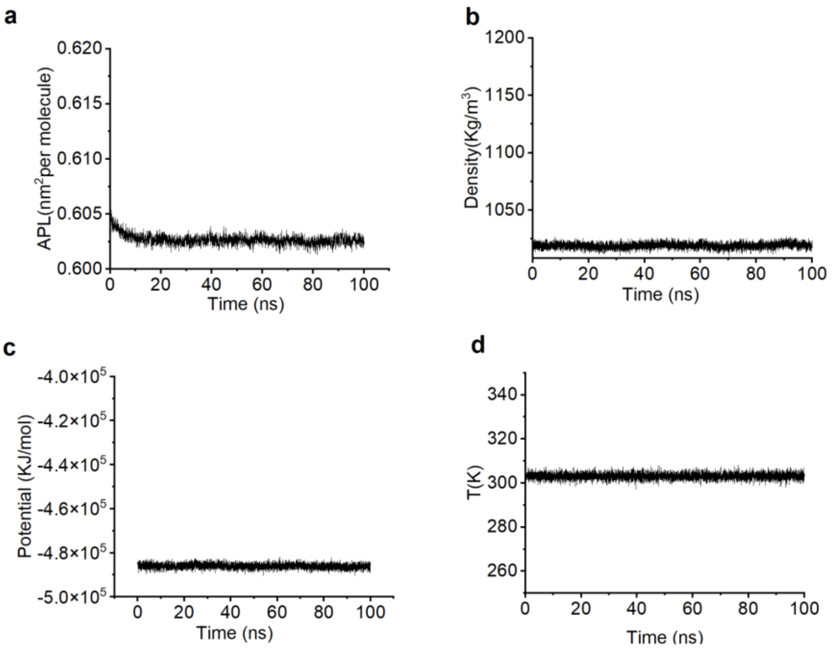

Figure S23. a) Variations in the surface area per lipid molecule (APL) during the 100 ns NPT equilibration of the phospholipid membrane; b) Changes in phospholipid density observed during the 100 ns NPT equilibration process; c) The energy profile of the phospholipid membrane system as it evolved during the 100 ns NPT equilibration; d) Temperature fluctuations recorded throughout the 100 ns NPT equilibration period.

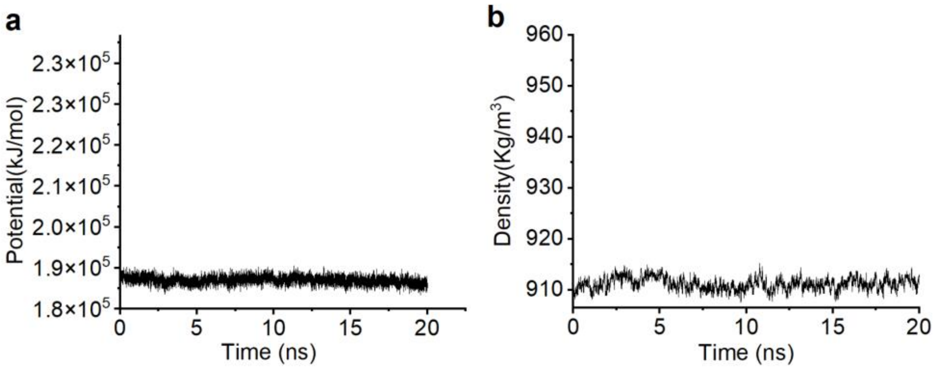

Figure S24. a) The energy profile of the TAG slab as it evolved during the 20 ns NPT equilibration; b) Changes in TAG density observed during the 20 ns NPT equilibration process.

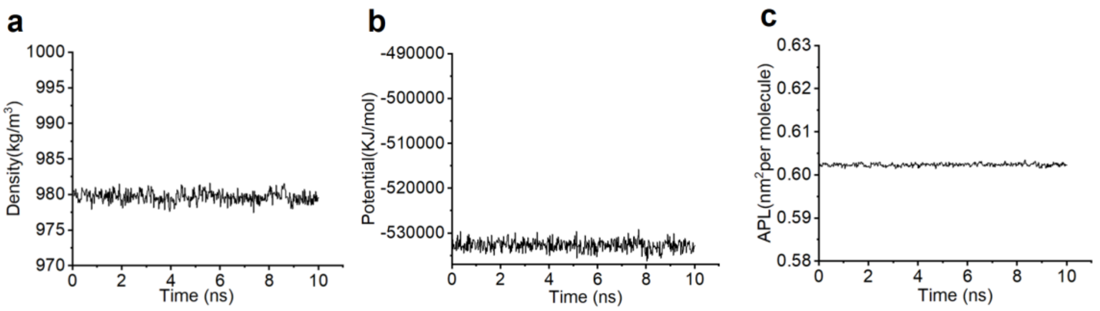

Figure S25. a) Evolution of system density during the 10 ns NPT equilibration of the lipid droplet simulation system. b) Variations in the system's energy throughout the 10 ns NPT equilibration process. c) Changes in the surface area per lipid molecule (APL) within the system during the 10 ns NPT equilibration of the lipid droplet simulation.

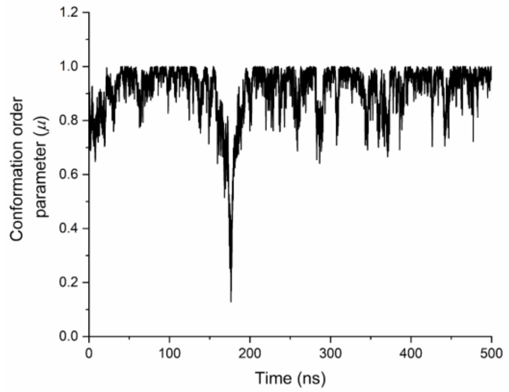

Figure S26. Time dependent change of conformation order parameter μ.

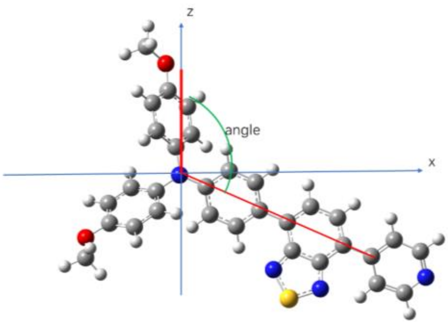

Figure S27. Defining an angle between the $R_{end\text{-}to\text{-}end}$ vector and the membrane normal.

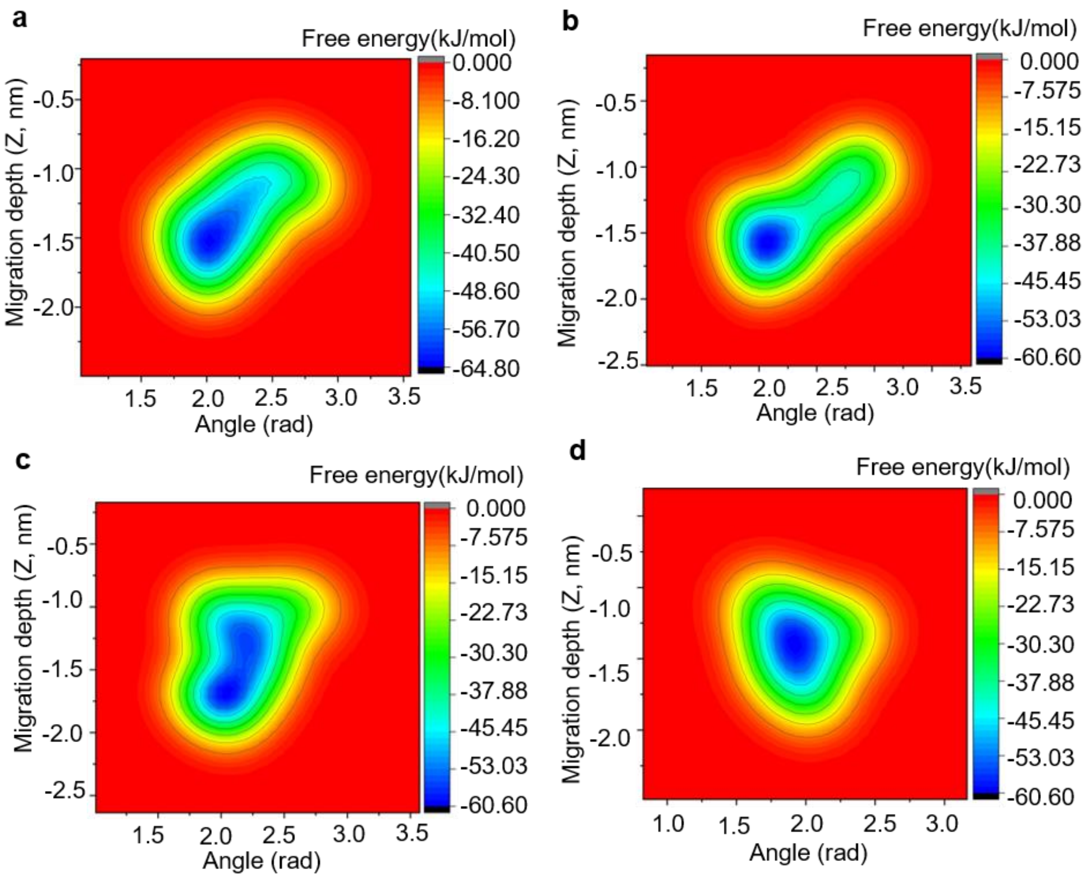

Figure S28. Migration depth, angle between the $R_{end\text{-}to\text{-}end}$ vector and the membrane normal, and free energy relationship of probe **TPABTBP** during the four independent meta-dynamics simulations. Initial position at a) z = -0.5 nm, b) z = -1.0 nm, c) z = -1.5 nm, d) z = -2.0 nm.

Initial position	Migration depth (nm)	Angle (rad)	Free energy (kJ/mol)
z=-0.5	-1.45	2.02	-63.31
z=-1.0	-1.54	2.08	-65.78
z=-1.5	-1.58	2.12	-58.09
z=-2.0	-1.50	1.93	-66.27

Figure S29. The average values and standard deviations of the migration depth, angle between the $R_{end\text{-}to\text{-}end}$ vector and the membrane normal, and free energy are -1.52±0.06 nm, 2.04±0.08 rad, and -63.36±3.75 kJ/mol, respectively.

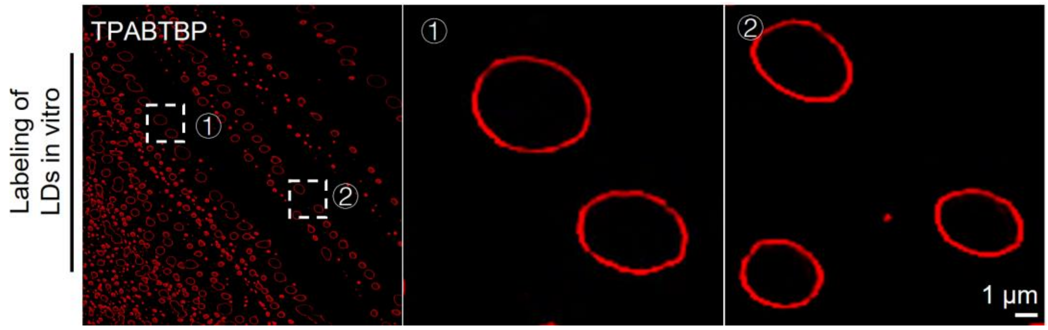

Figure S30. TPABTBP (10 μM) was used for confocal fluorescence imaging of isolated LDs from HepG2 cells. Scale bar: 1 μm.

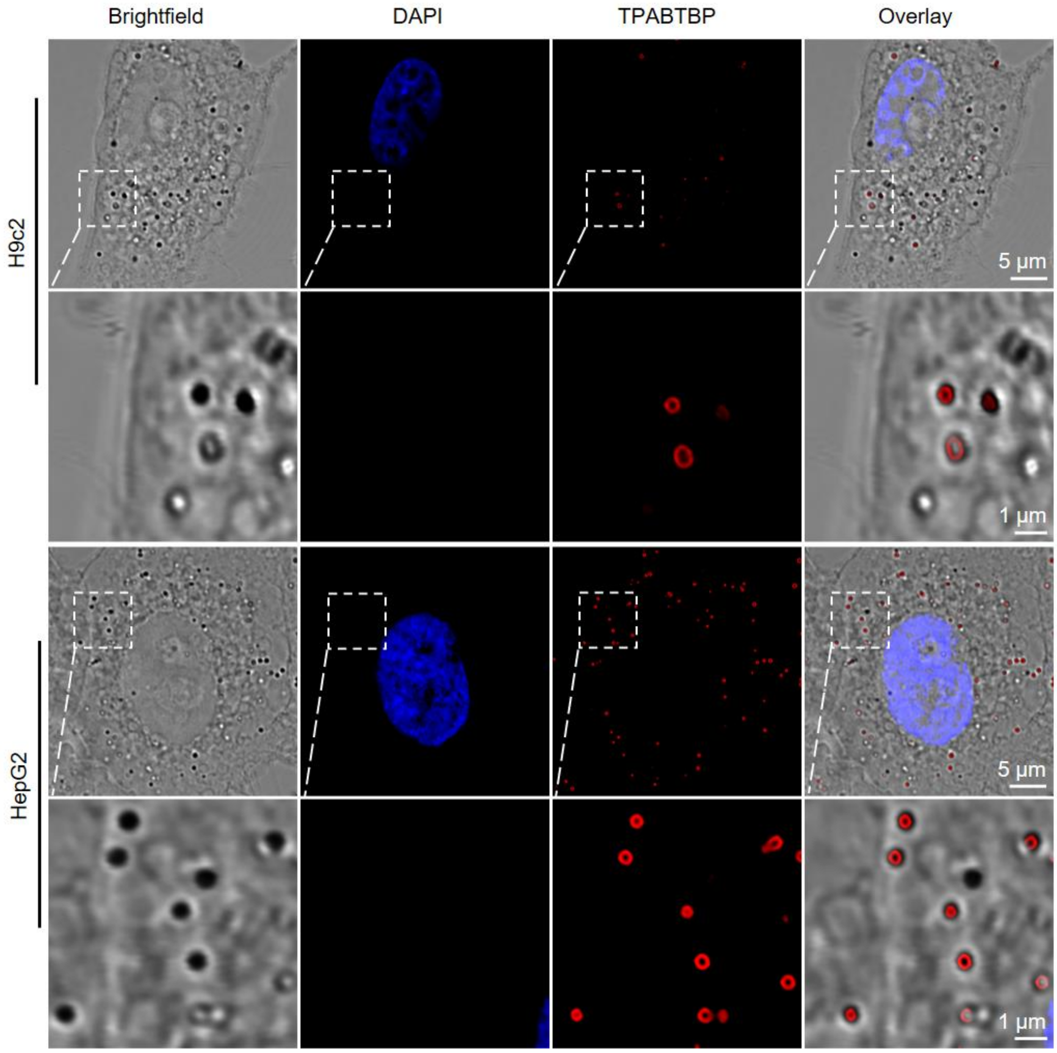

Figure S31. Confocal laser microscopy observation of the distribution of **TPABTBP** on LDs in H9c2 and HepG2 cells. Scale bar: 5 μm. Magnified image: 1 μm.

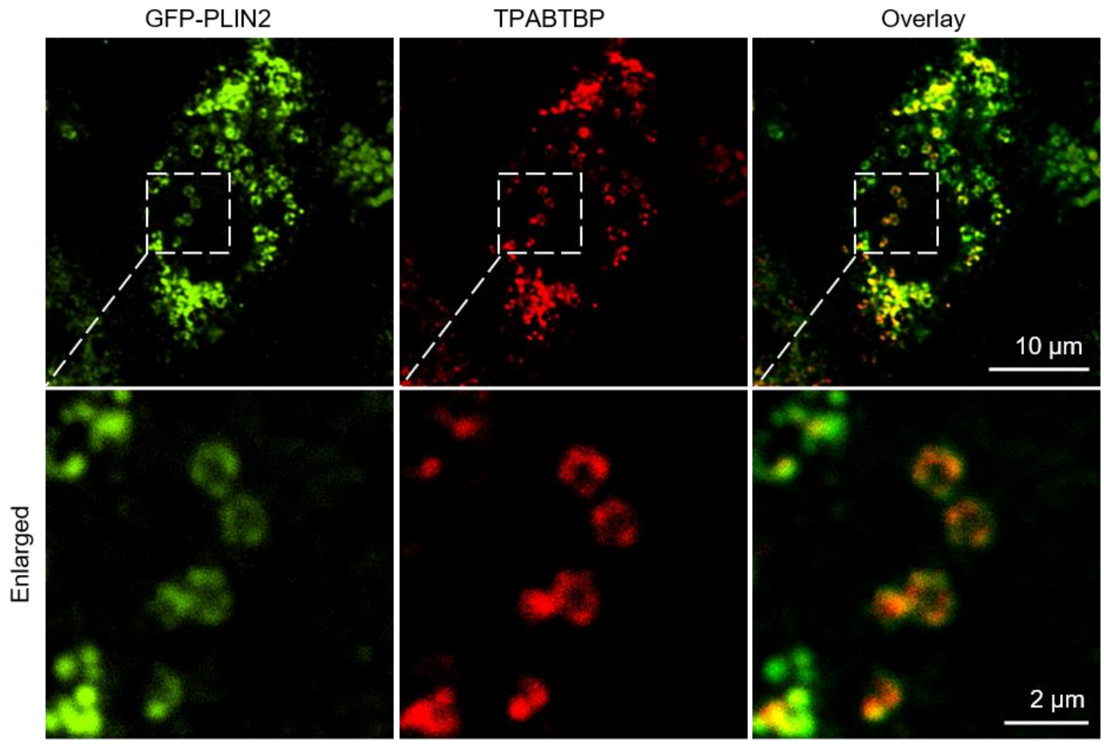

Figure S32. HepG2 cells expressing GFP-PLIN2 were treated with 200 μM OA for 12 h, then fixed and stained with **TPABTBP**. Cells were imaged by confocal microscopy. Scale bar: 10 μm. Magnified image: 2 μm.

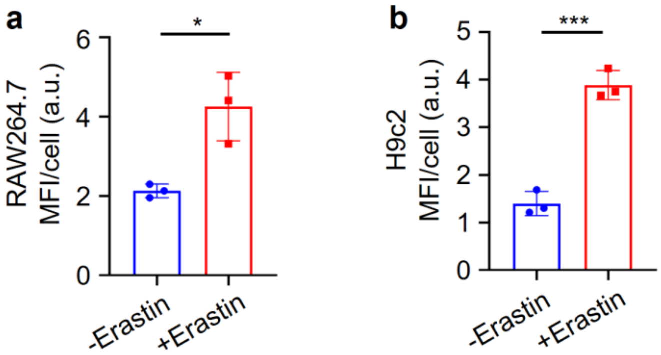

Figure S33. CLSM fluorescence intensities were quantified for a) RAW264.7 and b) H9c2 cells. The Cells were treated with Erastin (5 μM) for 24 h and then stained with **TPABTBP** (30 μM). n=3. *$P < 0.05$; ***$P < 0.001$.

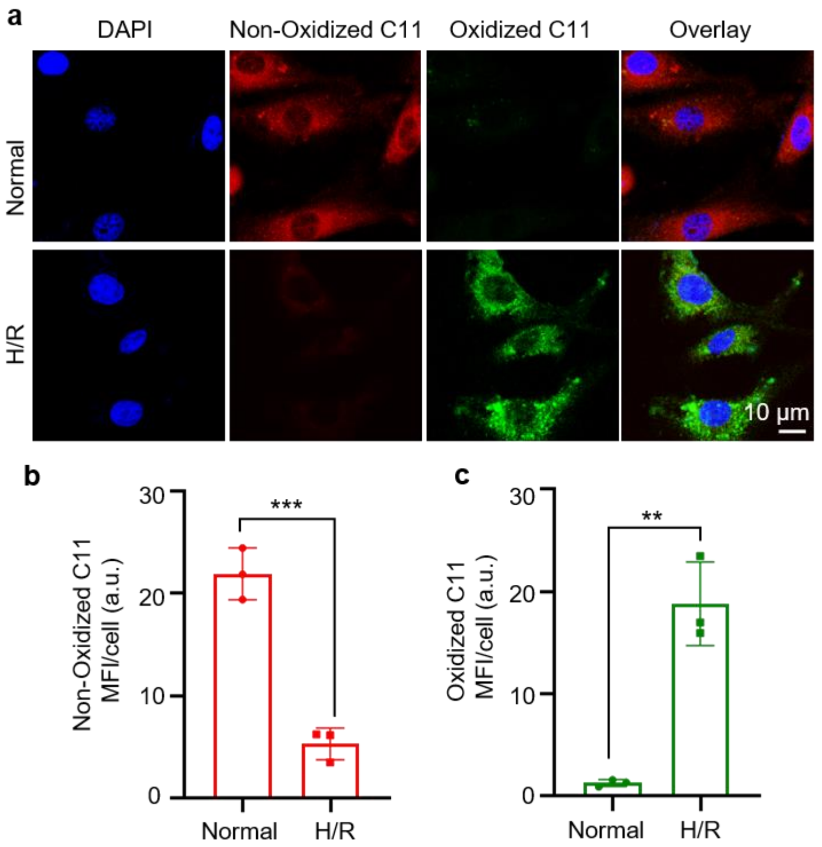

Figure S34. a) CLSM images of H9c2 cells exposed to hypoxic for 12 h and then reoxygenated for 2 h (H/R) and incubated with C11-BODIPY 581/591 (C11, 5 μM). Quantification of mean fluorescence intensity (MFI) of b) non-oxidized and c) oxidized C11-BODIPY 581/591 (n=3, ** p <0.01, *** p <0.001).

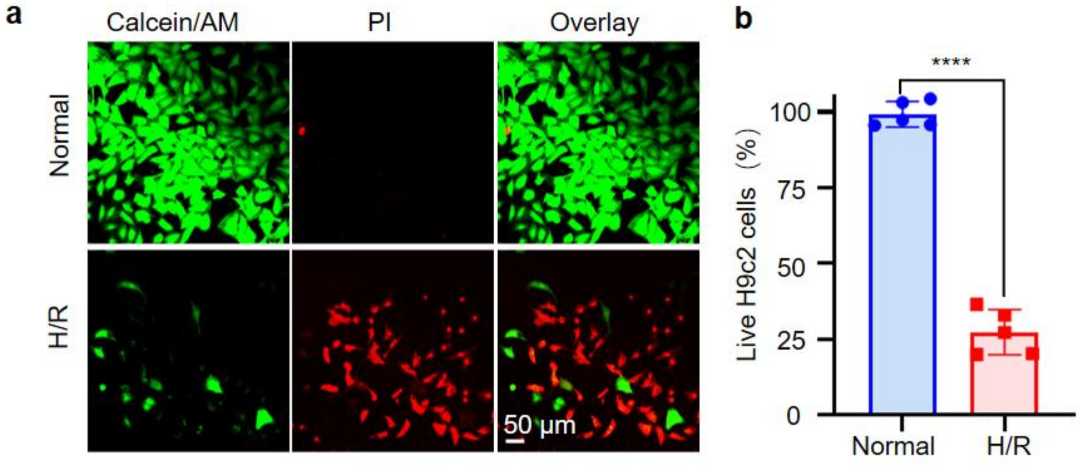

Figure S35. a) CLSM images of H9c2 cells exposed to hypoxic for 12 h and then reoxygenated for 2 h (H/R) and incubated with Calcein-AM/PI. b) Quantification of the mean fluorescence intensity (MFI) of Calcein-AM for evaluating the live cells ratio (n=5, **** P<0.0001).

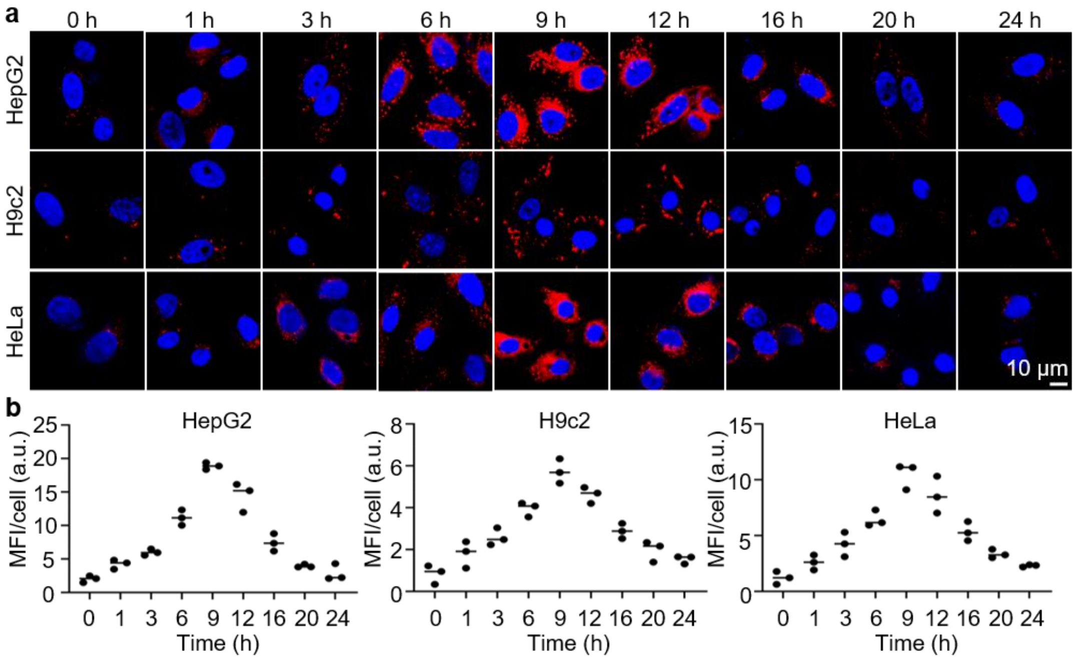

Figure S36. Dynamic changes in LD count during Erastin-induced ferroptosis in living cells. a) Confocal fluorescence imaging of LDs using **TPABTBP** (5 μM) after induction with Erastin (5 μM) at different time points (0, 1, 3, 6, 9, 12, 16, 20, and 24 h) in HepG2, H9c2, and HeLa cells. Scale: 10 μm. b) Quantification of mean fluorescence intensity (MFI). n=3.

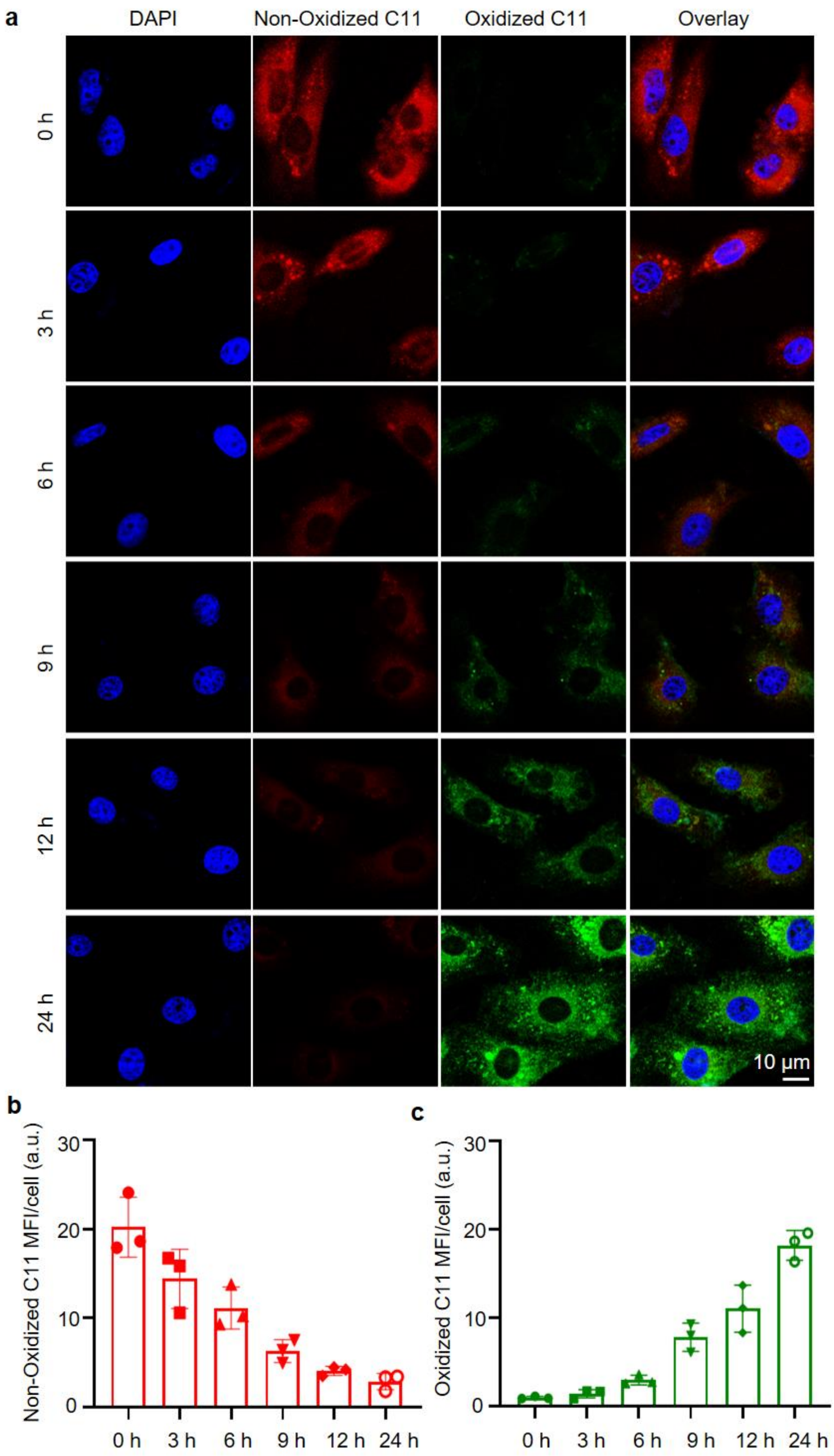

Figure S37. a) The lipid peroxide levels of H9c2 induced by 5 μM Erastin were observed by confocal microscopy at different time points (0, 3, 6, 9, 12 and 24 h). Quantitative analysis of mean fluorescence intensity (MFI) of b) non-oxidized and c)

oxidized C11-BODIPY 581/591(n = 3).

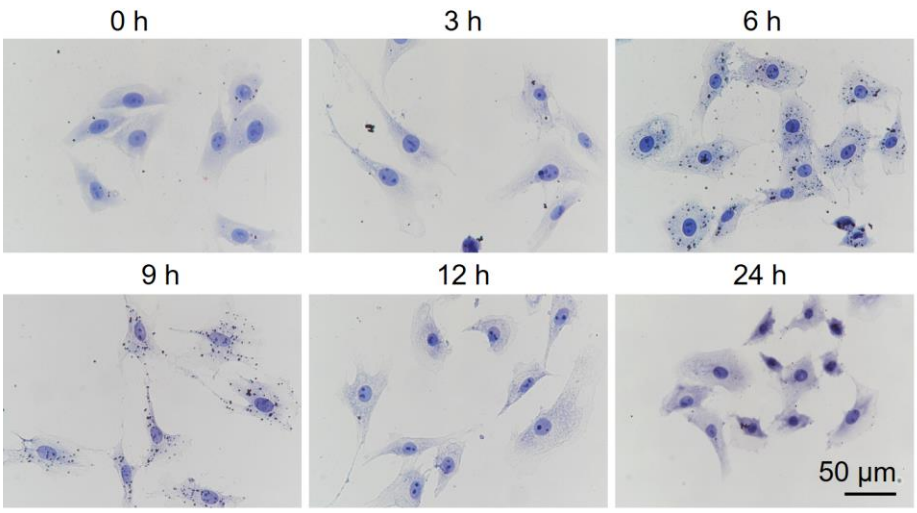

Figure S38. Oil red O staining was used to monitor the contents of LDs induced by 5 μM Erastin for 0 h, 3 h, 6 h, 9 h, 12 h, 24 h. Scale :50 μm.

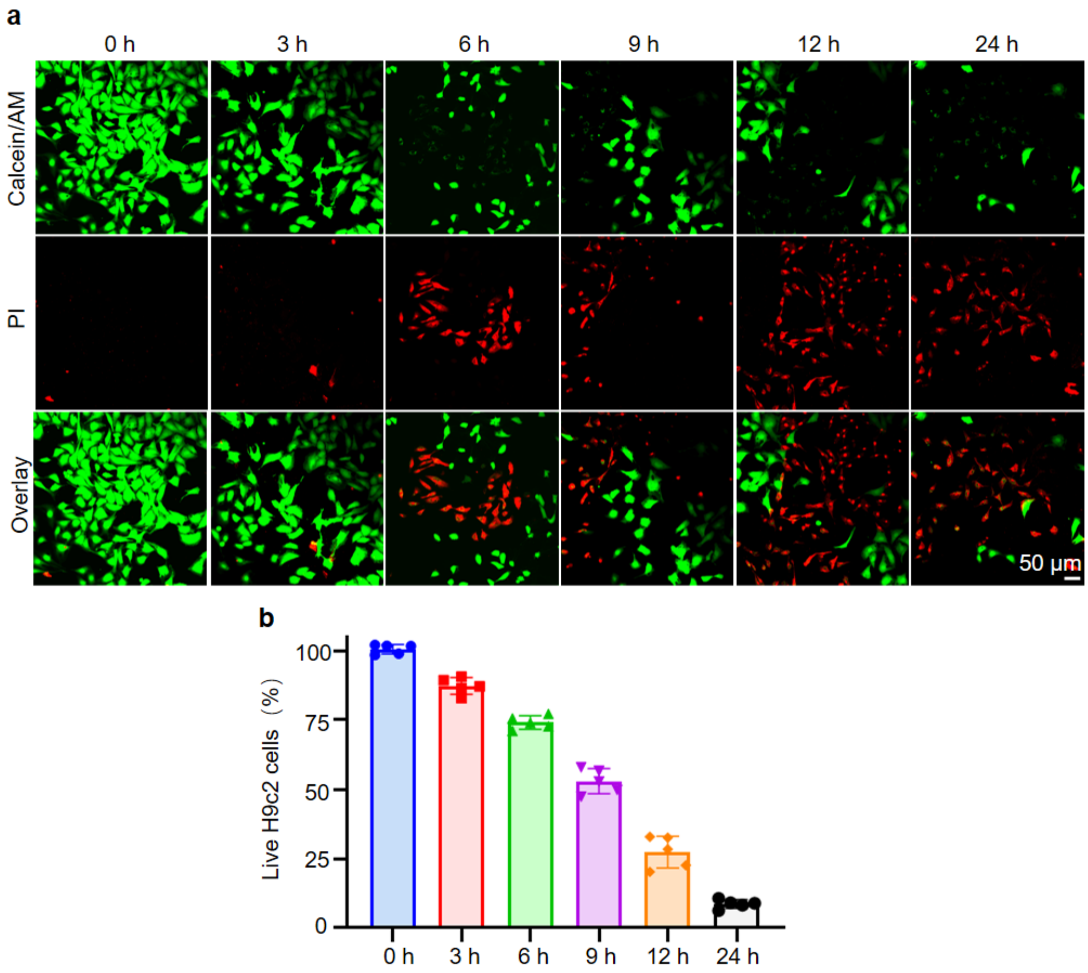

Figure S39. a) Calcein-AM/PI staining was employed to assess the cell viability of H9c2 cells treated with 5 μM Erastin, which were subsequently observed using

confocal microscopy at various time points (0, 3, 6, 9, 12, and 24 h). Scale :50 μm. b) Quantification of the mean fluorescence intensity (MFI) of Calcein-AM for the analysis of the proportion of live H9c2 cells (n = 5).

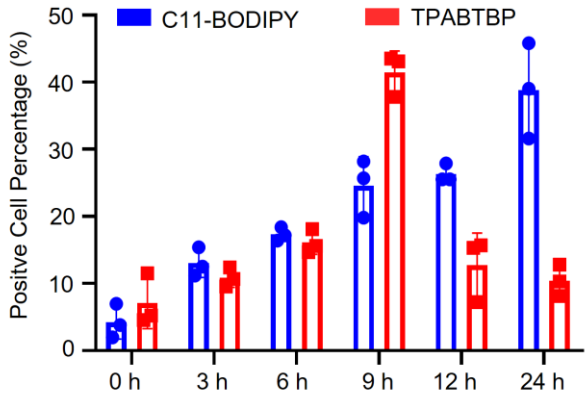

Figure S40. Flow cytometry was used to detect the proportion of **TPABTBP** (30 μM) and C11-BODIPY 581/591 (5 μM) labeled positive cells at different time points (0 h, 3 h, 6 h, 9 h, 12 h, 24 h). n=3.

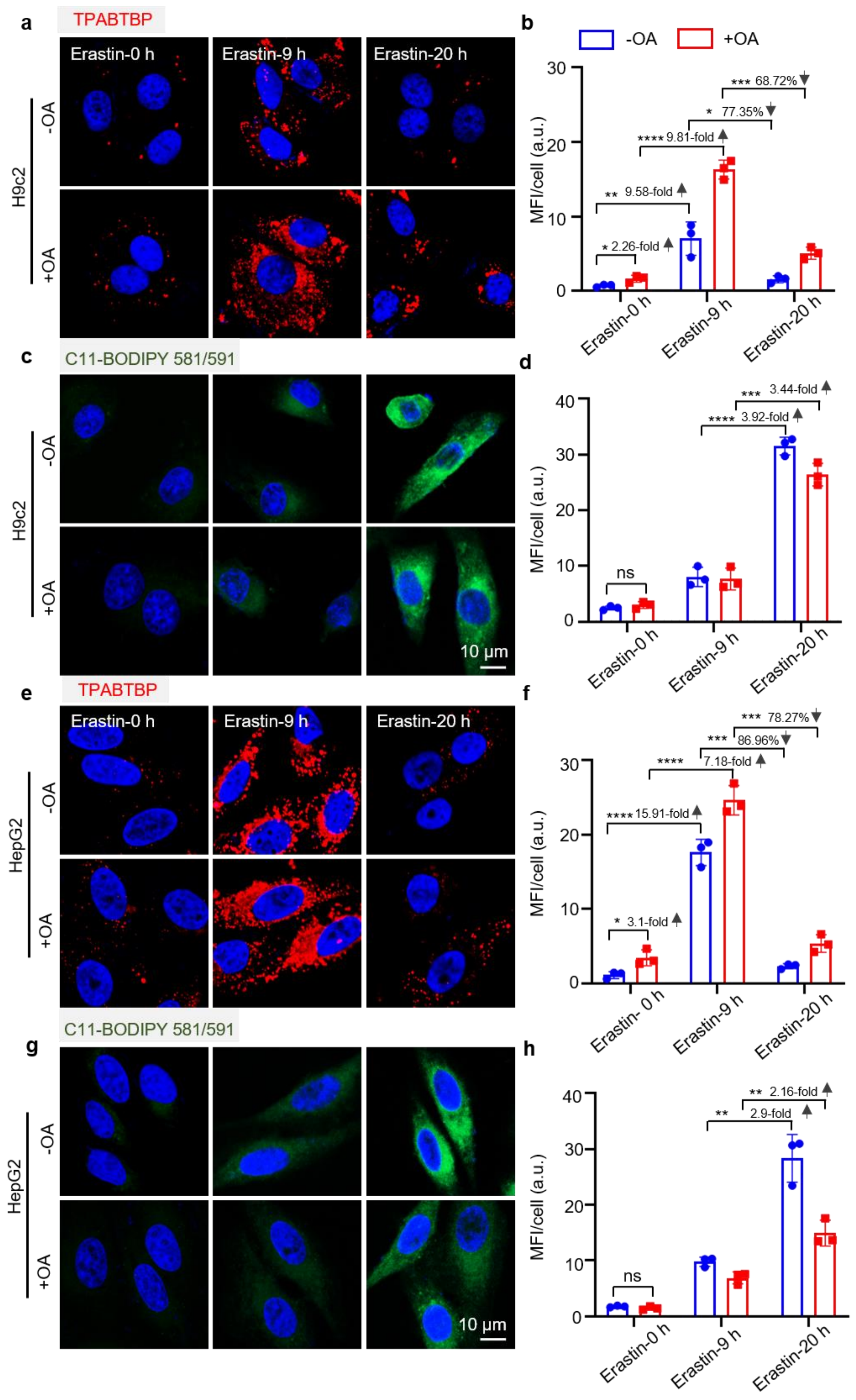

Figure S41. Detection of LDs and lipid peroxidation during ferroptosis in the context

of 1μM oleic acid (OA) pretreatment for 16 h. a) Representative confocal images depict LDs labeled with **TPABTBP** in OA-pretreated H9c2 cells, followed by induction with 5 μM Erastin. b) The mean fluorescence intensity (MFI) of **TPABTBP**, representing LDs, in H9c2 cells induced with Erastin was determined from fluorescent images using ImageJ. c) Confocal microscopy images were obtained to assess lipid peroxidation using C11-BODIPY 581/591 in H9c2 cells pretreated with OA during Erastin induction. d) The MFI analysis of C11-BODIPY 581/591 in H9c2 cells pretreated with OA followed by induction with Erastin was performed based on fluorescent images. e) Confocal microscopy images and f) quantification were conducted to assess LDs in OA-pretreated HepG2 cells during Erastin induction. The level of lipid peroxidation was evaluated in OA-pretreated HepG2 cells induced with Erastin using g) confocal images and h) quantitative assessment. Scale :10 μm. n=3; $^{**}P < 0.01$; $^{***}P < 0.001$; $^{****}P < 0.0001$.

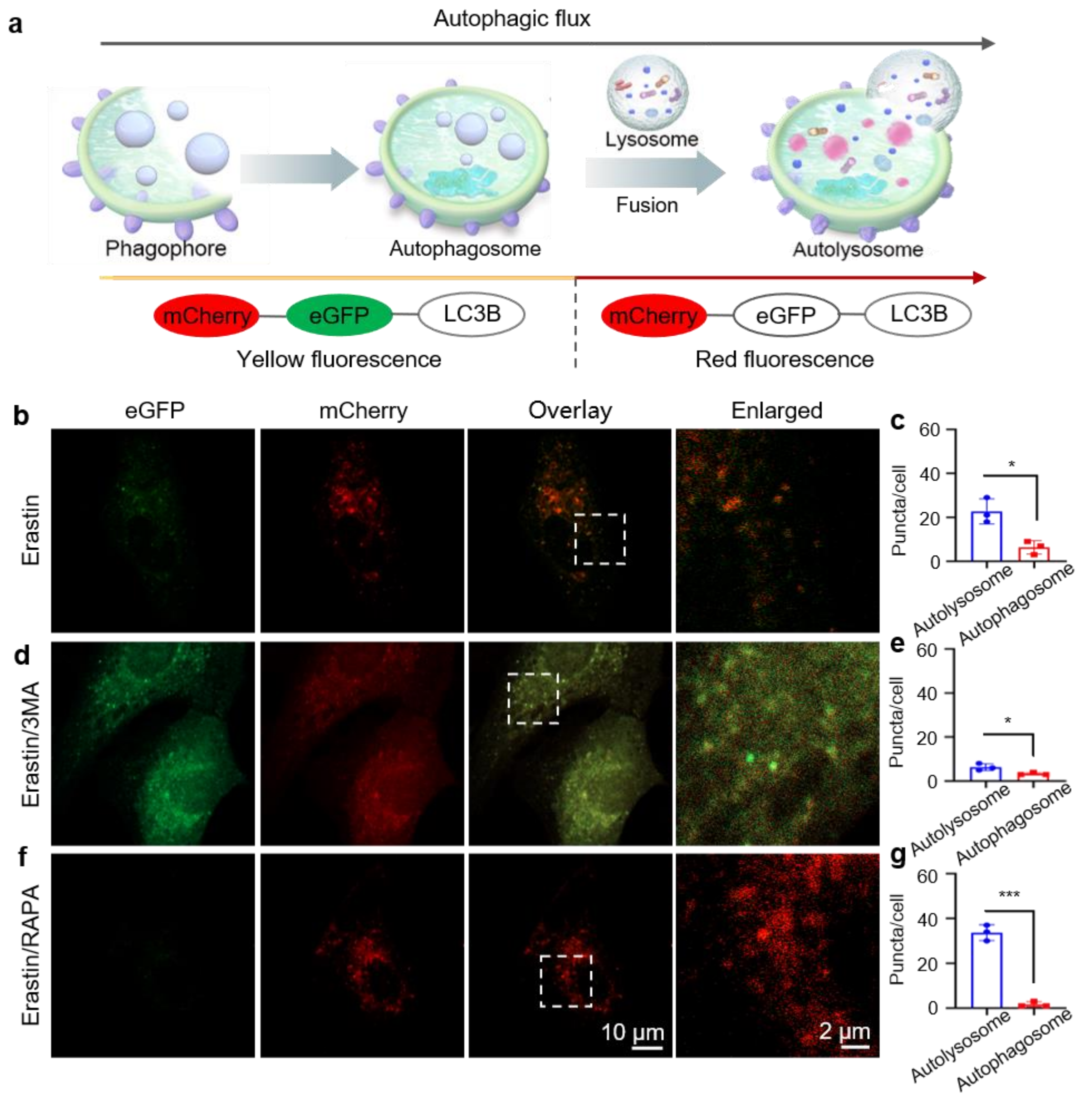

Figure S42. a) Schematic diagram of autophagy flux detection. b-g) Representative confocal images and quantification of autophagosome and autolysosome numbers in H9c2 cells treated with 3-MA (10 mM, 9 h) and RAPA (100 nM, 9 h), respectively. Prior to treatment with small molecular drugs, H9c2 cells were transfected with mCherry-eGFP-LC3 adenovirus and then induced with 5 μM Erastin for 20 h. Scale: 10 μm. Magnified image: 2 μm. n=3; **P* < 0.05; ****P* < 0.001.

Table S3. sgRNA target sequences for knockout ATG5 in H9c2 cell line

sgRNA	Target Sequence (5'-3')
g5	GTGATAGGTTGTGCGGAAGT TGG
g6	AGAAACTTACTCCGTGGTTT AGG

Table S4. Primers for PCR

Gene	Primer Sequence (5'-3')
ATG5	F GTGGTAAAGATAGTAGCAGGCC
	R CGGAACTGCTCAACGGTGC

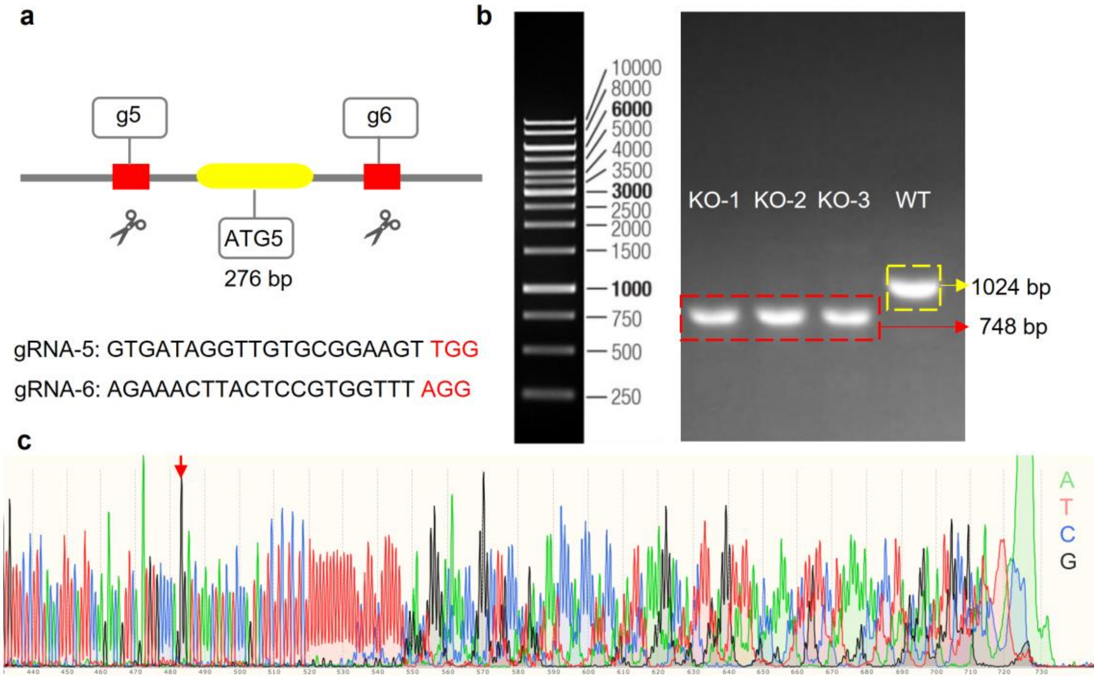

Figure S43. a) Scheme of ATG5 knockout strategy. b) Representative PCR gel with ATG5 knockout (KO). KO-1, KO-2, and KO-3 represent three individual monoclonal samples post gene knockout, while WT denotes the wild-type. Red box: WT was identified by 1024-bp band; yellow box: ATG5 KO was identified by 748-bp band. c) ATG5 KO H9c2 cells were confirmed by Sanger sequencing. Red arrow indicates the starting point of the knockout sequence.

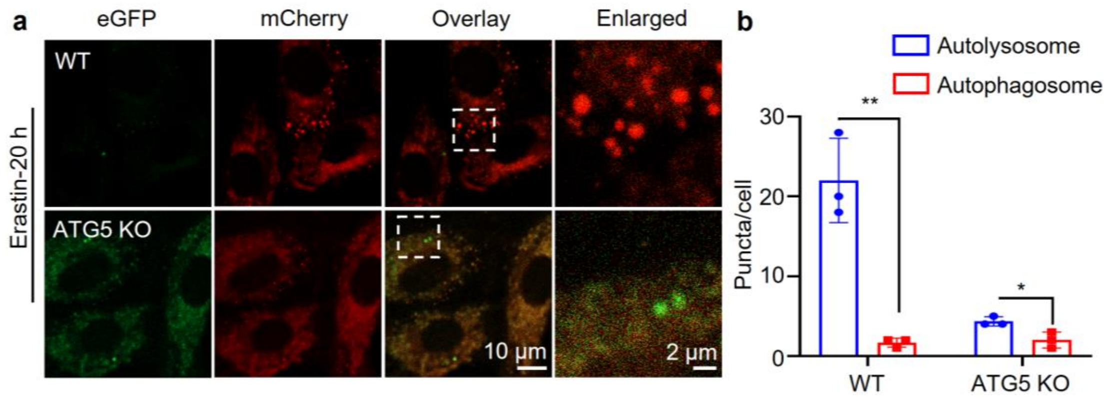

Figure S44. a) CLSM images and b) quantification of autophagosome and autolysosome numbers in wild type (WT) and ATG5 knock out (ATG5 KO) H9c2 cells incubated with 5 μM Erastin for 20 h. Prior to treatment with Erastin, the H9c2 cells were transfected with mCherry-eGFP-LC3 adenovirus. Scale: 10 μm. Magnified image: 2 μm. n=3. Student's t tests were performed to calculate the statistical significance; **P* < 0.05; ***P* < 0.01.

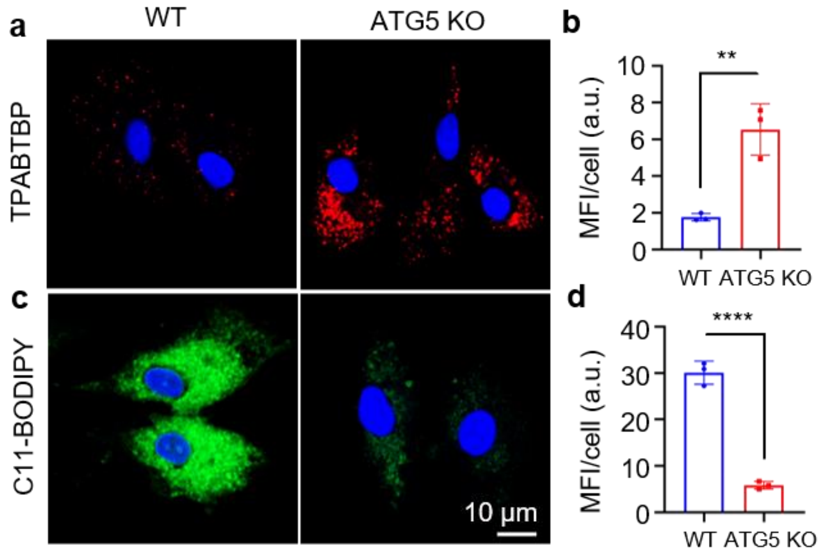

Figure S45. a) Confocal imaging was employed to investigate LDs and c) lipid peroxidation levels in wild-type (WT) and ATG5 knockout (KO) H9c2 cells treated with 5 μM Erastin for 20 h. Scale: 10 μm. Quantitation of the mean fluorescence intensity (MFI) of cells stained by b) **TPABTBP** or d) C11-BODIPY 581/591; n=3. Student's t tests were performed to calculate the statistical significance; ***P* < 0.01; ****P* < 0.001; *****P* < 0.0001.

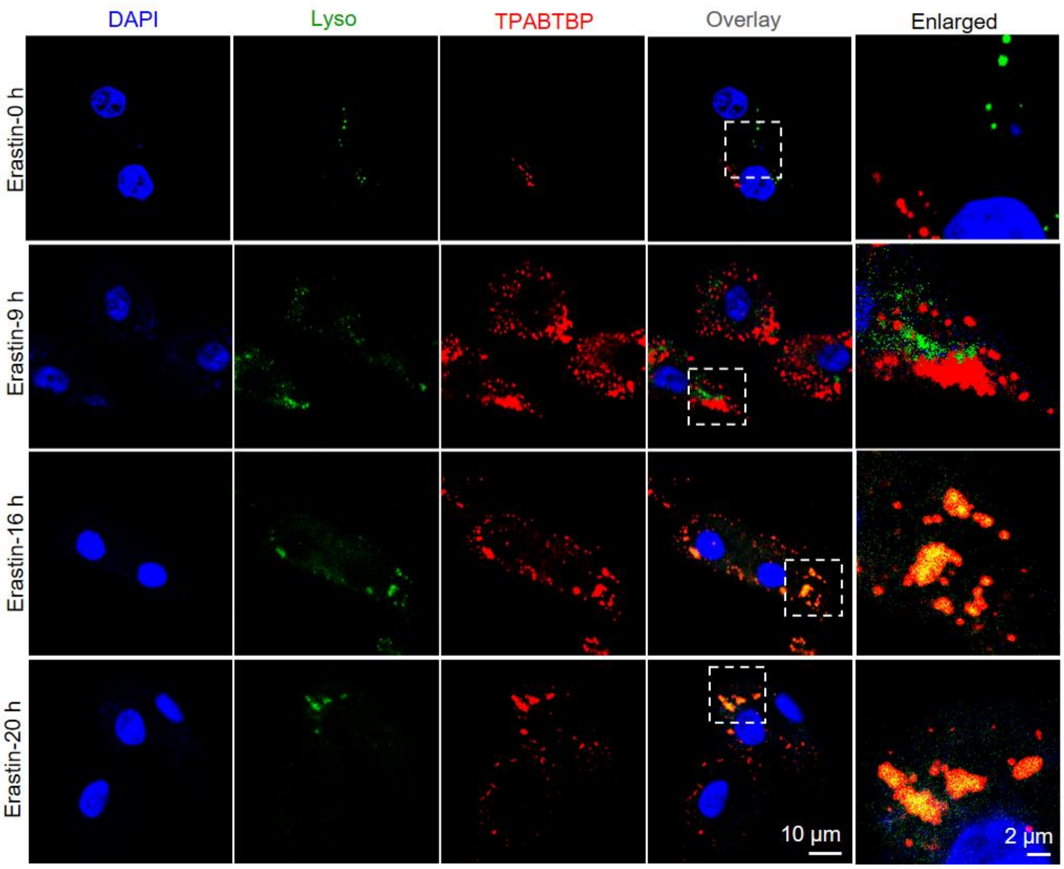

Figure S46. H9c2 cells pre-treated with Erastin (5 μM) at different time nodes, followed by incubation with **TPABTBP** (30 μM) and LysoTracker (75 nM). Scale bar: 10 μm. Magnified image: 2 μm.

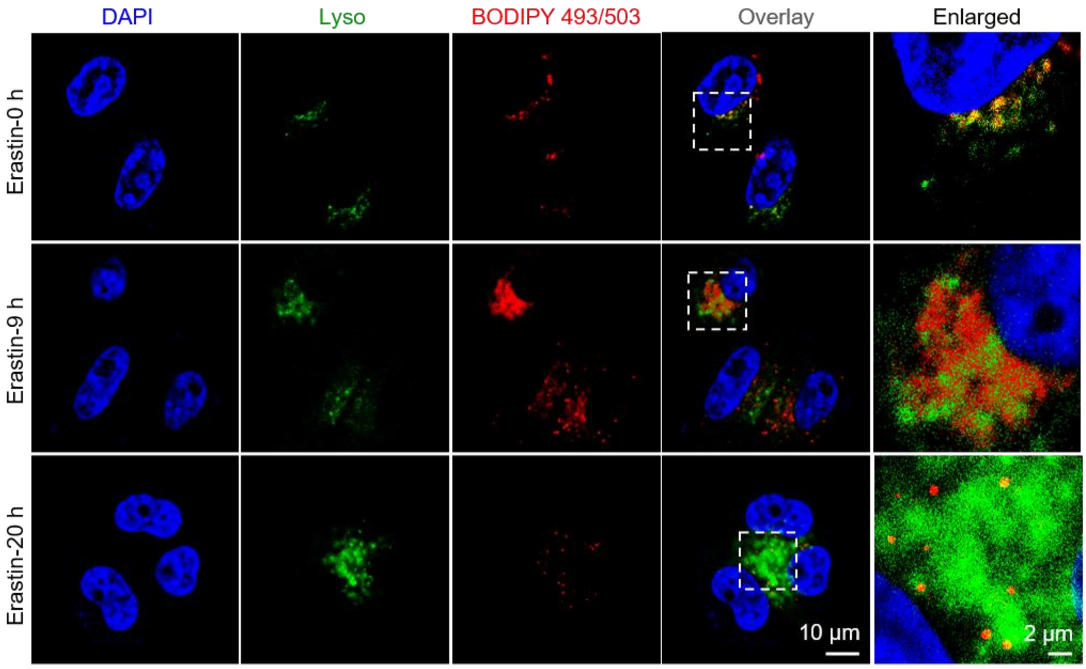

Figure S47. CLSM imaging of H9c2 cells incubated with BODIPY493/503 (5 μM) and LysoTracker (75 nM). Prior to incubation with imaging probes, cells were pre-treated with Erastin (5 μM) at various time points. Scale bar: 10 μm. Magnified image: 2 μm.

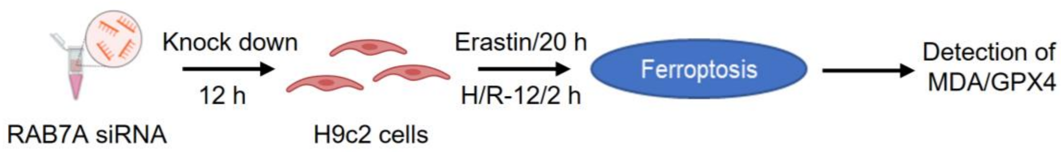

Figure S48. Scheme of the H/R or Erastin during ferroptosis with RAB7A siRNA in cardiomyocyte.

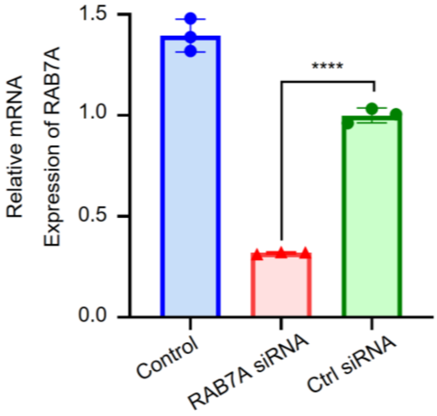

Figure S49. Analysis of relative mRNA expression of RAB7A by PCR. The Ctrl siRNA representing control siRNA. n = 3; Significance was calculated using the Student's t test; ****$P < 0.0001$.

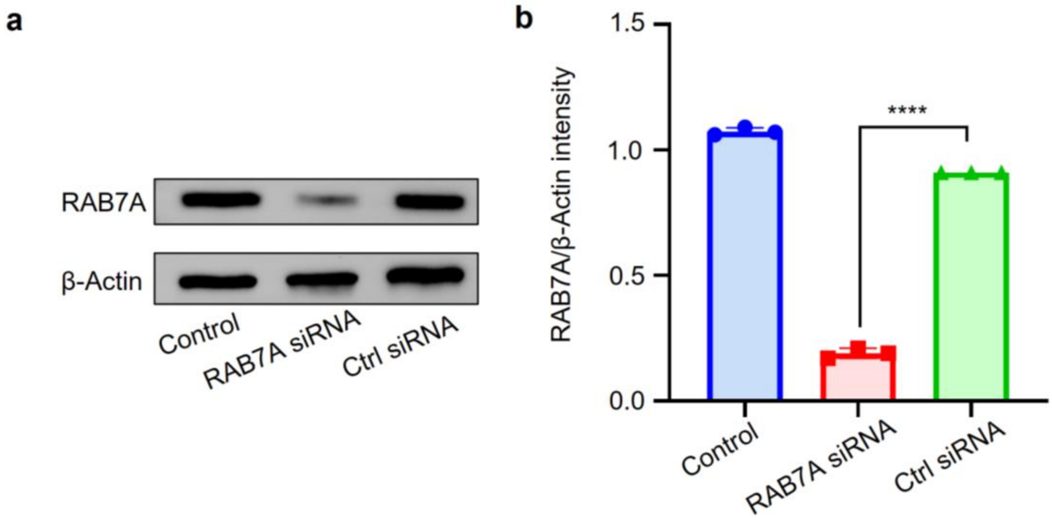

Figure S50. a) Western blot analysis of RAB7A expression and b) the quantification of the ratio of RAB7A to β-Actin. The Ctrl siRNA representing control siRNA. n = 3, Significance was calculated using the Student's t test; ****P < 0.0001.

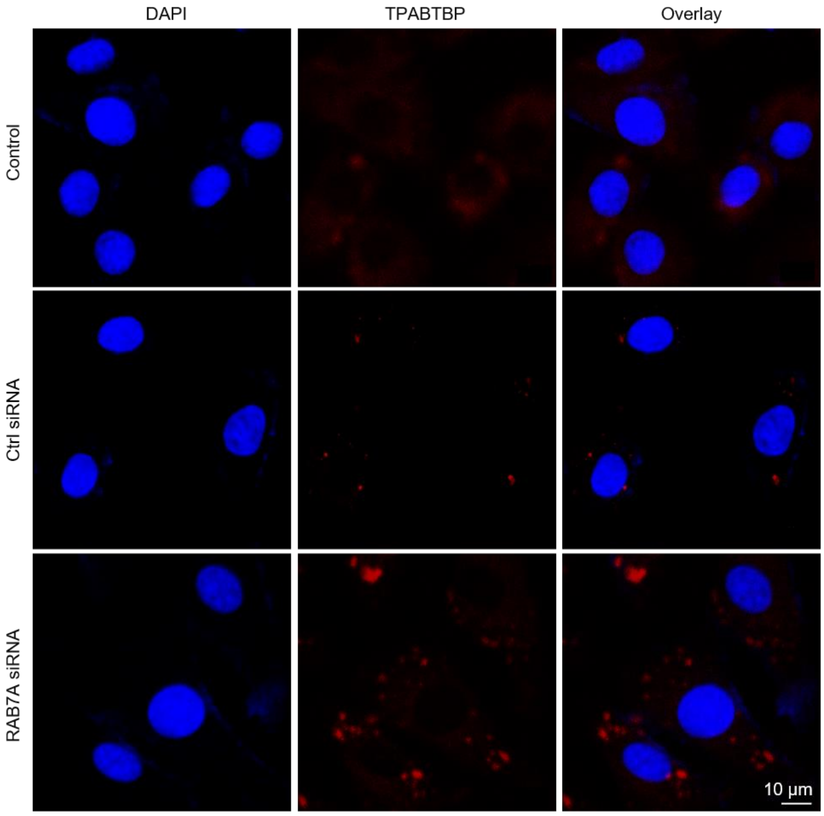

Figure S51. Effect of RNA silencing on H9c2 cells. Scale :10 μm.

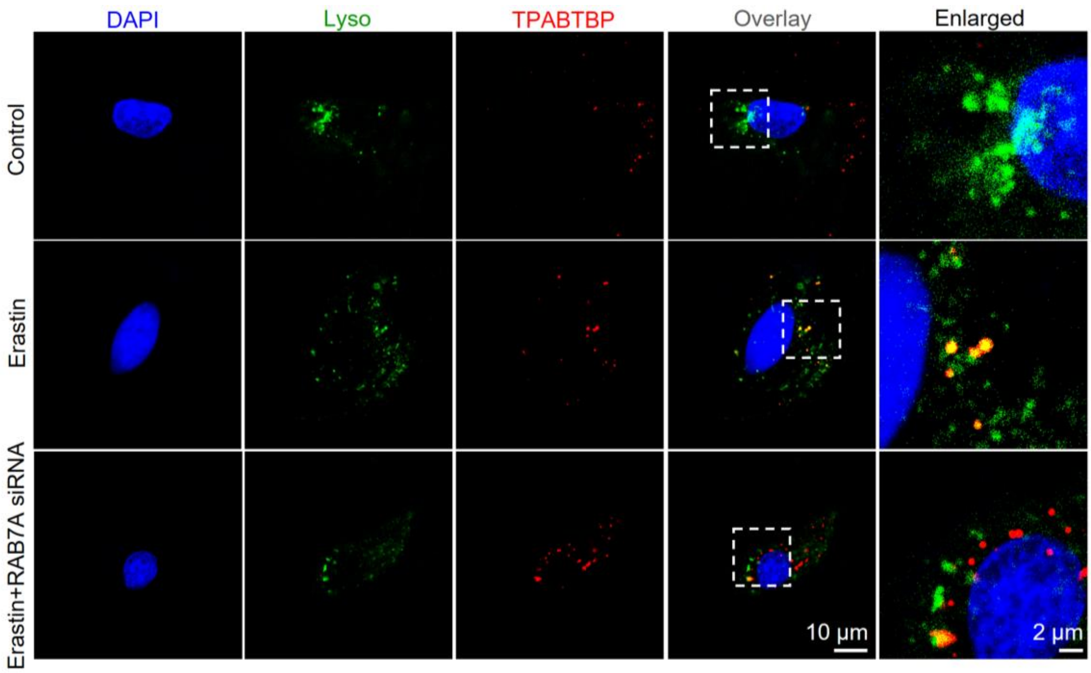

Figure S52. CLSM imaging of normal and RAB7A-knockdown H9c2 cells incubated

with **TPABTBP** (30 μM) and LysoTracker (75 nM). Prior to incubation with imaging probes, cells were pre-treated with Erastin (5 μM) for 20 h. Scale bar: 10 μm; Magnified image: 2 μm.

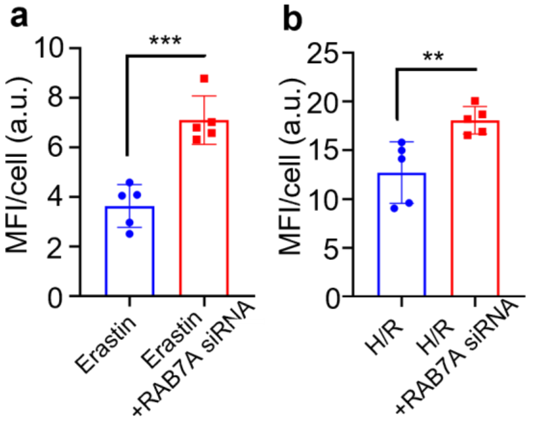

Figure S53. a) Evaluation of mean fluorescence intensity (MFI) in normal and RAB7A knockdown H9c2 cells under Erastin induction. b) Assessment of MFI in normal and RAB7A knockdown H9c2 cells under H/R conditions. n=5. Significance was calculated using the Student's t test; $^{**}P < 0.01$, $^{***}P < 0.001$.

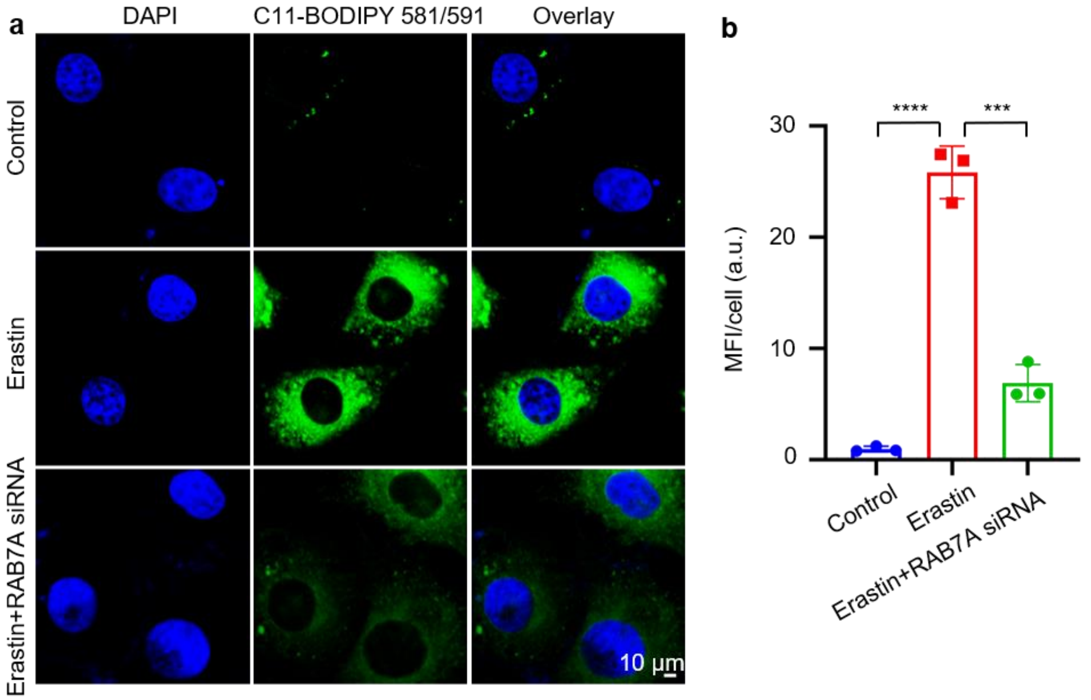

Figure S54. a) The lipid peroxidation levels in two types of H9c2 cells (RAB7A-knockdown H9c2 cells and normal H9c2 cells) were evaluated after induction with 5 μM Erastin for 20 h. b) The fluorescence intensity of C11-BODIPY 581/591 were

quantified. Scale bar: 10 µm; n=5. Significance in b was calculated using the Student's t test; ***$P < 0.001$, ****$P < 0.0001$.

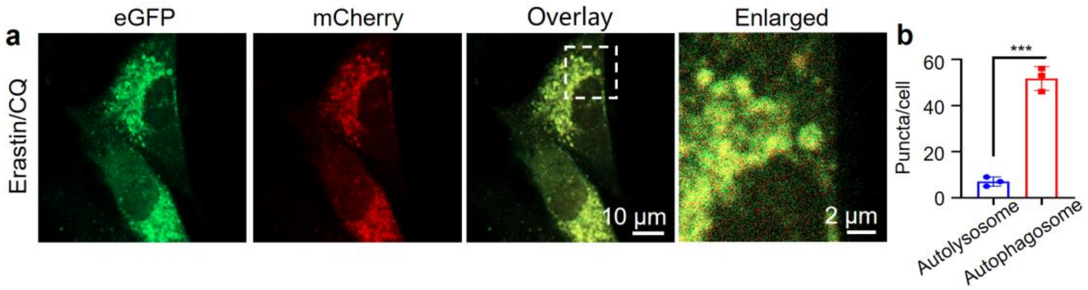

Figure S55. a) Representative confocal images and b) quantification of autophagosome and autolysosome numbers in H9c2 cells treated with CQ (5 µM, 10 h). Prior to treatment with CQ, the H9c2 cells transfected with mCherry-eGFP-LC3 adenovirus were induced with 5 µM Erastin for 20 h; n=3, ***$P < 0.001$. Scale: 10 µm. Magnified image: 2 µm.

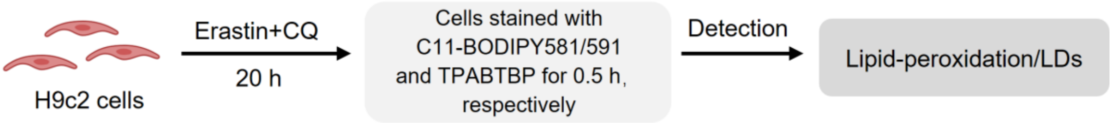

Figure S56. Scheme of the CQ treatment with Erastin during ferroptosis in cardiomyocyte.

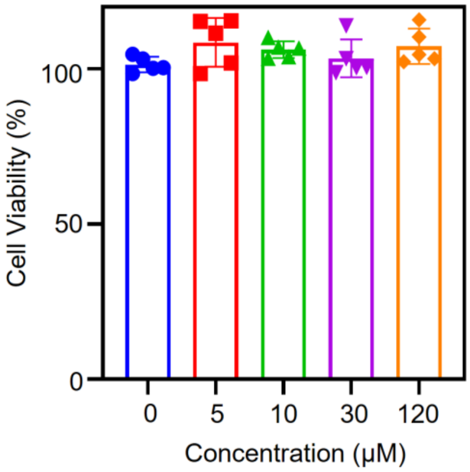

Figure S57. Viabilities of H9c2 cells were measured by CCK-8 after incubated with **TPABTBP** (0, 5, 10, 30, and 120 µM) for 24 h, n=5.

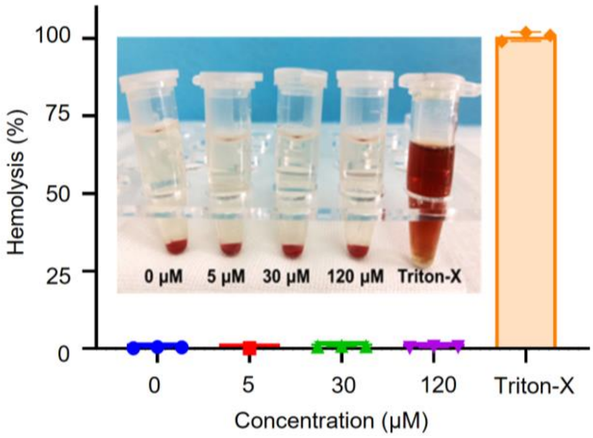

Figure S58. Quantification of hemolysis rate of **TPABTBP** with different concentrations (0, 5, 10, 30, and 120 μM), n=3.

Table S5. Blood routine analysis of PBS and **TPABTBP** group (2 mg/kg).

**Hematologic Parameters**	**PBS**	**TPABTBP**
White blood cell（10^9/L）	2.6±0.8	1.6±1.0
Lymphocytes（10^9/L）	2.1±0.7	0.9±0.5
Monocytes（10^9/L）	0.06±0.05	0.06±0.05
Granulocyte（10^9/L）	0.5±0.12	0.6±0.4
Red blood cell（10^{12}/L）	7.7±1.4	8.0±0.7
Hemoglobin（g/L）	109±23.1	113±10
Hematocrit（%）	36.8±6.7	37.4±2.5
Mean corpuscular volume（fL）	47.8±0.2	46.8±1.2
Mean corpuscular hemoglobin（pg）	14.1±0.4	14.1±0.05
Mean corpuscular hemoglobin concentration（g/L）	294.7±9.3	302±7.8
Red blood cell volume distribution width（%）	14.6±0.1	14.0±0.05
Mean platelet volume（fL）	4.9±0.3	5.3±0.3
Platelet distribution width	16.2±0.3	16.9±0.7

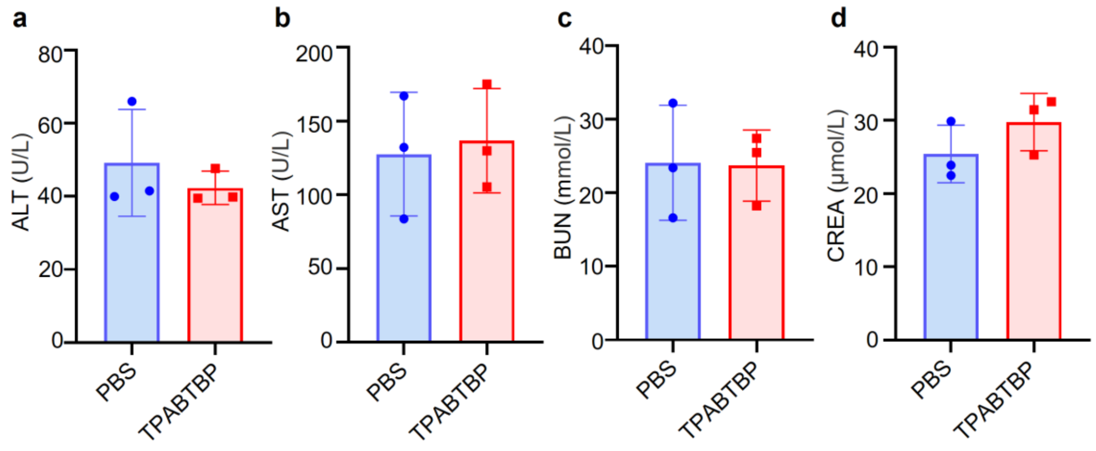

Figure S59. Blood biochemistries of (a) ALT, (b) AST, (c) BUN and (d) CREA were measured after the injection with **TPABTBP** (2 mg/kg), n=3.

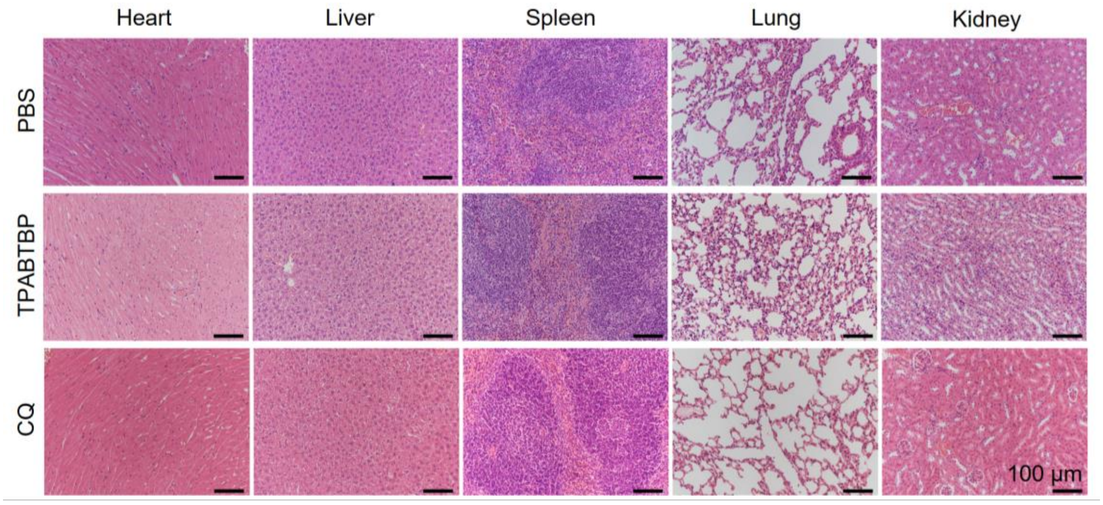

Figure S60. H&E staining of the heart, liver, spleen, lung and kidney of the mouse after injected with **TPABTBP** (2 mg/kg) or CQ (30 mg/kg). Scale :100 μm.

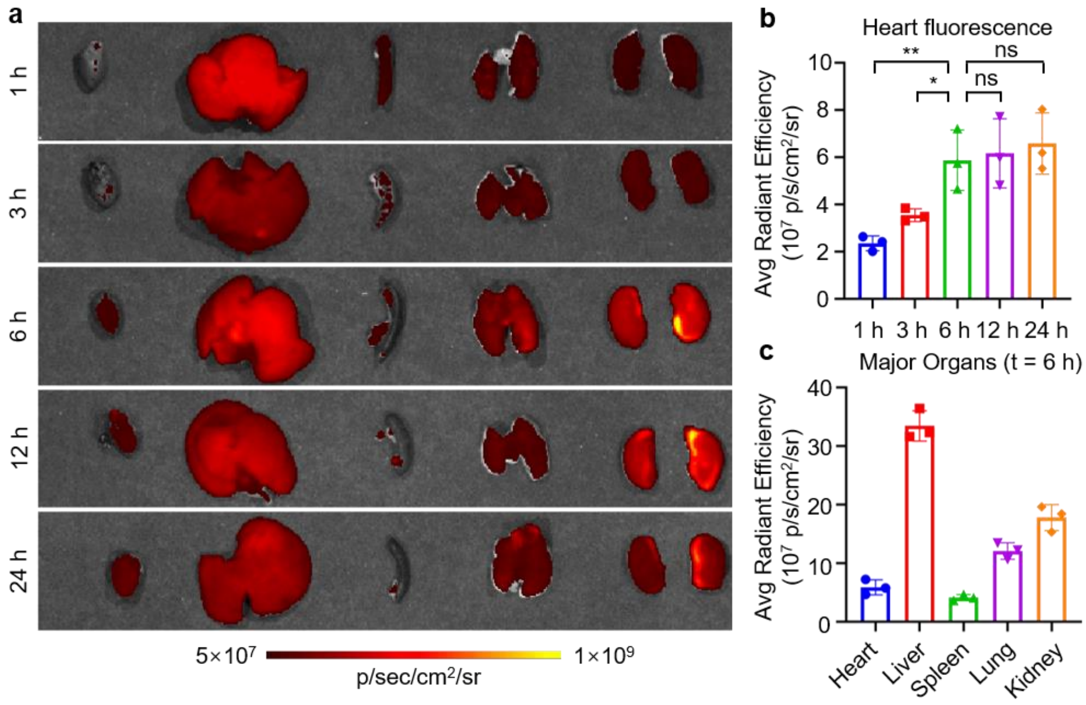

Figure S61. Distribution of **TPABTBP** (2 mg/kg) in main organs following intravenous injection. a) Representative ex vivo fluorescence image of main organs. b) Quantification of fluorescence in the heart at different post-injection time (1 h, 3 h, 6 h, 12 h and 24 h). c) Quantification of fluorescence in main organs in 6 h of administration; n=3; *$P < 0.05$; **$P < 0.01$.

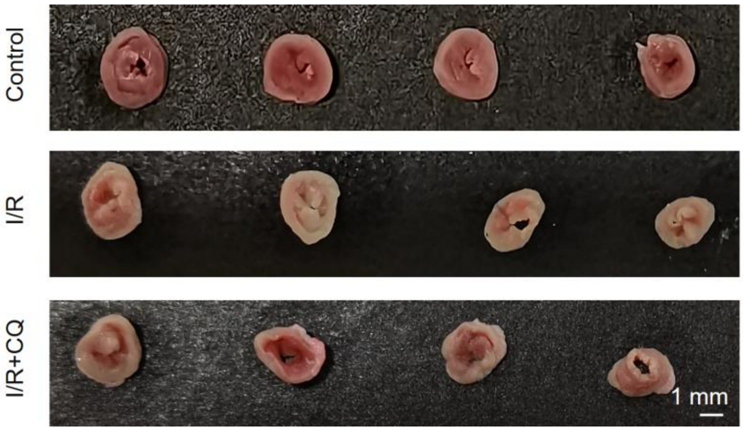

Figure S62. TTC staining of heart sections in control (untreated mice), I/R and CQ treatment groups.

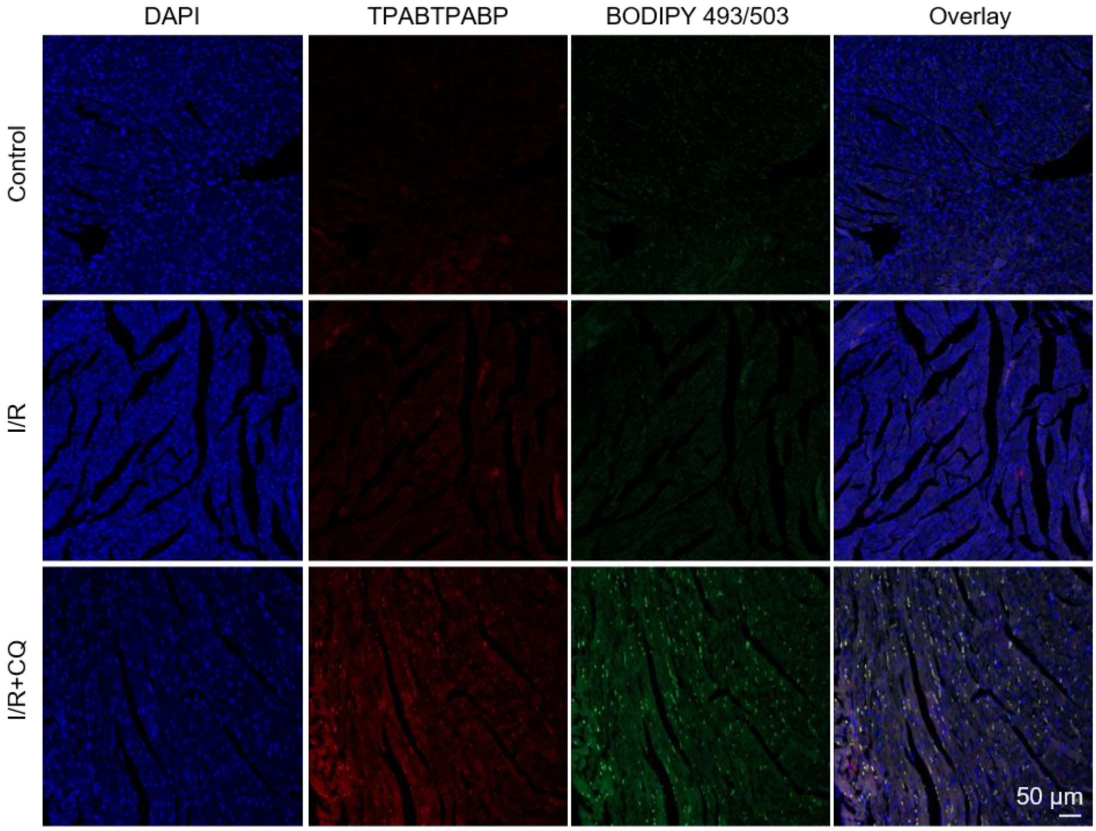

Figure S63. Images of frozen myocardial tissue sections co-incubated with **TPABTBP** (30 μM) and BODIPY 493/503 (5 μM). Scale :50 μm.

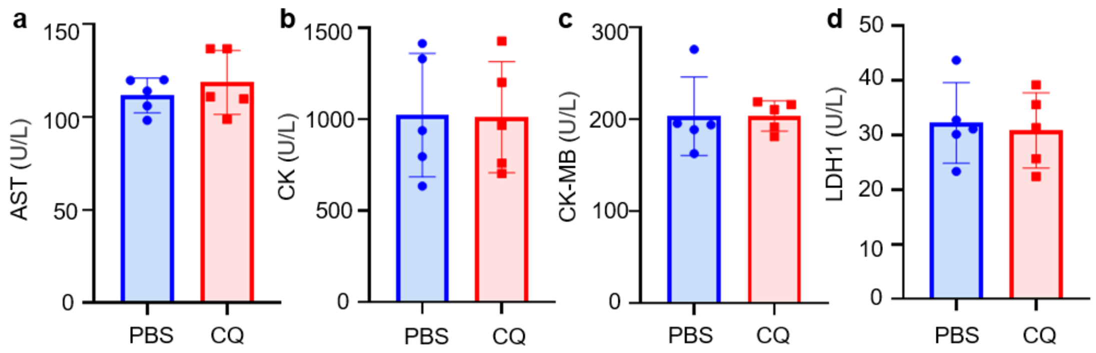

Figure S64. Comparison of the myocardial enzymes of a) AST, b) CK, c) CK-MB, d) LDH1 of mice injected with CQ (30 mg/kg), n=5.

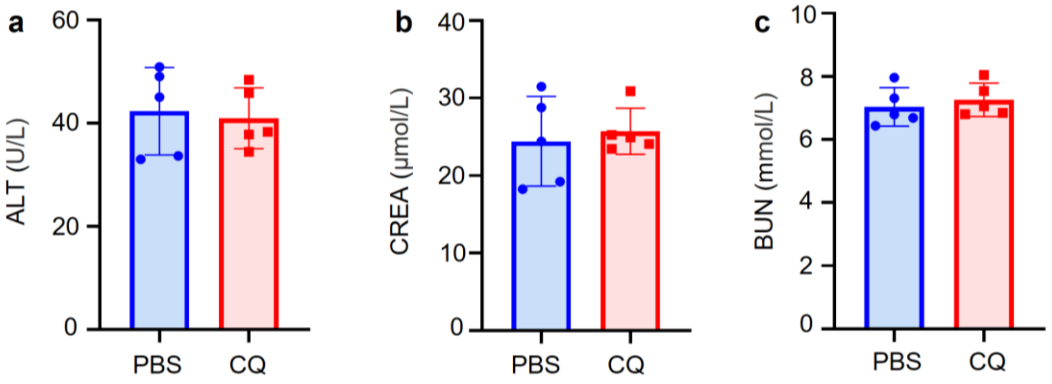

Figure S65. Detection of the myocardial enzymes of (a) ALT, (b) CREA and (c) BUN of mice treated with CQ (30 mg/kg), n=5.

Reference

1. J. Zheng, S. Zhao, Y. Mao, Z. Du, G. Li, M. Sang, *Small* **2022**, 18, 2104471.

2. Ye, Z.; Ji, M.; Wu, K.; Yang, J.; Liu, A. A.; Sun, W.; Ding, D.; Liu, D, *Angew. Chem. Int. Ed.* **2022**, 61, e202204518.

3. M. Sang, B. Cai, S. Qin, S. Zhao, Y. Mao, Y. Wang, X. Yu, J. Zheng, *ACS Appl. Mater. Inter.* **2021**, 13, 58369.

4. S. Li, W. Zhuang, J. Chen, G. Li, C. Li, L. Chen, Y. Liao, M. Chen, Y. Wang, *J. Mater. Chem. B.* **2021**, 9, 4050.

5. Z. Zhan, W. Zhuang, Q. Lei, S. Li, W. Mao, M. Chen, W. Li, *Chem. Commun.* **2022**, 58, 4020.

6. C. Wu, X. Li, T. Zhu, M. Zhao, Z. Song, S. Li, G. Shan, G. Niu, *Anal. Chem.* **2022**, 94, 3881.

7. L. Fan, X. D. Wang, Q. Zan, L. F. Fan, F. Li, Y. M. Yang, C. H. Zhang, S. M. Shuang, C. Dong, *Anal. Chem.* **2021**, 93, 8019

8. P. Tan, W. Zhuang, S. Li, J. Zhang, H. Xu, L. Yang, Y. Liao, M. Chen, Q. Wei, *Chem. Commun.* **2021**, 57, 1046

9. J. Chen, C. Wang, W. Liu, Q. Qiao, H. Qi, W. Zhou, N. Xu, J. Li, H. Piao, D. Tan, X. Liu, Z. Xu, *Angew. Chem., Int. Ed.* **2021**, 25104

10. G. W. Jiang, Y. Jin, M. Li, H. L. Wang, M. Y. Xiong, W. L. Zeng, H. Yuan, C. L. Liu, Z. Q. Ren, C. R. Liu, *Anal. Chem.* **2020**, 92, 10342

11. H. Xu, H. Zhang, G. Liu, L. Kong, X. Zhu, X. Tian, Z. Zhang, R. Zhang, Z. Wu, Y. Tian, H. Zhou, *Anal. Chem.* **2019**, 91, 977

12. C. Y. Zhang, H. R. Shao, J. Zhang, X. Y. Guo, Y. Liu, Z. G. Song, F. Liu, P. X. Ling, L. G. Tang, K. N. Wang, Q. X. Chen, *Theranostics* **2021**, 11, 7767

13. K. Wang, S. Ma, Y. Ma, Y. Zhao, M. Xing, L. Zhou, D. Cao, W. Lin, *Anal. Chem.* **2020**, 92, 6631
